# Total Synthesis of (±)-Aspidospermidine, (±)-Aspidofractinine, (±)-Limaspermidine, and (±)-Vincadifformine via a Cascade and Common Intermediate Strategy

**DOI:** 10.1021/acs.joc.2c02099

**Published:** 2022-10-19

**Authors:** David L. Cain, Niall A. Anderson, David B. Cordes, Alexandra M. Z. Slawin, Allan J. B. Watson

**Affiliations:** † EaStCHEM, School of Chemistry, University of St Andrews, North Haugh, Fife, St AndrewsKY16 9ST, U.K.; ‡ GlaxoSmithKline, Medicines Research Centre, Gunnels Wood Road, StevenageSG1 2NY, U.K.

## Abstract

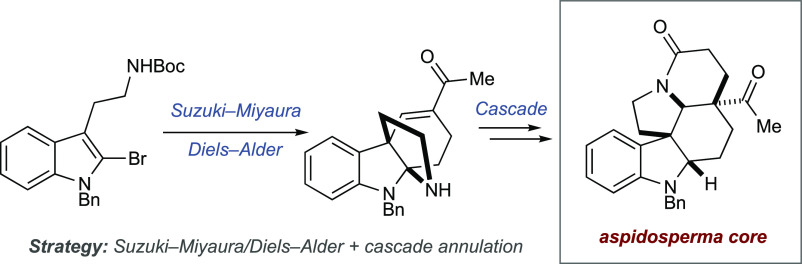

A concise strategy for the total synthesis of several *Aspidosperma* alkaloids is reported. A Suzuki–Miyaura cross-coupling provides access to a 2-vinyl indole that undergoes a Diels–Alder cascade reaction with butyn-2-one to deliver a pyrroloindoline intermediate. This undergoes cascade amidation, reduction, skeletal rearrangement, and intramolecular Michael addition to provide a common intermediate containing the full framework of the *Aspidosperma* alkaloids. The utility of this intermediate is shown in the synthesis of four different natural products.

## Introduction

**Scheme 1:**
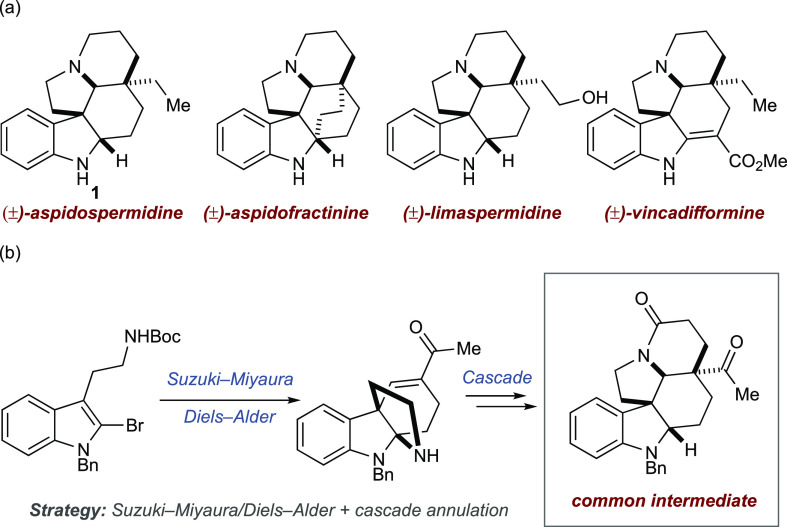
(a) *Aspidospermidine* and Several Related Alkaloids and (b) This Work: Divergent Synthesis via a Cascade and Common Intermediate Strategy

The *Aspidosperma* alkaloids are a class of monoterpenoid indole alkaloids, which have seen significant interest as synthetic targets (Scheme 1a).^1−4^ Their intriguing, densely fused polycyclic structures have inspired numerous strategies and methodologies to enable access to the eponymous aspidospermidine (**1**),^5−8^ as well as many other members of this family. Of the strategies employed in total synthesis, divergent approaches based on a common intermediate are arguably the most powerful.^8a,9^ These approaches allow the synthesis of multiple natural products by late-stage modification of a single common intermediate.

Here, we report a common intermediate strategy for the synthesis of *Aspidosperma* natural products, where the common intermediate is accessed through cascade reactions, and demonstrate the utility of this cascade strategy in the synthesis of (±)-aspidospermidine,^5−9^ (±)-aspidofractinine,^10^ (±)-limaspermidine,^11^ and (±)-vincadifformine (Scheme 1b).^8d,12^

## Results and Discussion

**Scheme 2:**
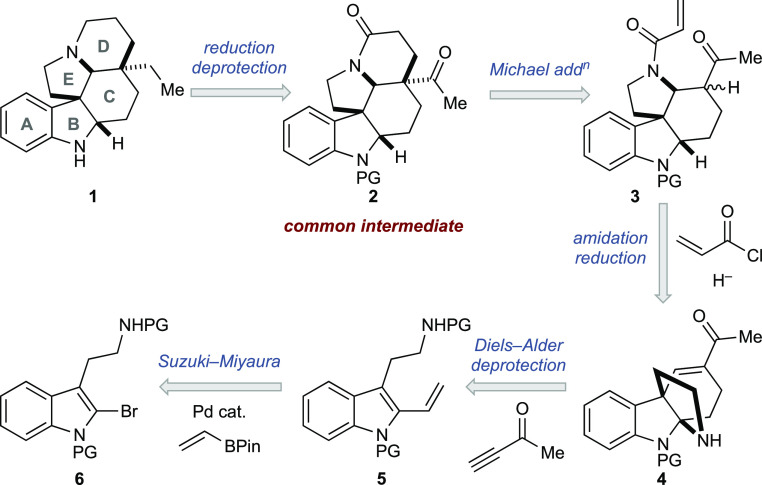
Retrosynthetic Strategy

Our retrosynthetic approach is shown in Scheme 2.

We envisioned that (±)-aspidospermidine (**1**) could be accessed by straightforward reduction and deprotection of **2**, which contained the full carbon skeleton of **1**. Indeed, **2** also aligned with our desired divergent synthesis approach as it could be elaborated in several straightforward ways to allow the synthesis of several other members of this class of natural products. As such, **2** was our targeted common intermediate for this overall synthetic campaign. Inspired by previous work,^8a^ we believed that **2** could be swiftly accessed in cascade amidation, reduction, skeletal rearrangement, and intramolecular Michael addition by treatment of pyrroloindoline **4** with acryloyl chloride, a suitable hydride reagent, and a base. The key intermediate **4** would be accessed from vinyl indole **5** via a cascade using the Diels–Alder reaction and intramolecular trapping of the generated iminium by the pendant tryptamine. Vinyl indole **5** would be prepared via Suzuki–Miyaura coupling of bromoindole **6**. Based on this proposed sequence, it seemed likely that a cascade synthesis of **4** could be realized beginning from **6**.^13,14^

**Scheme 3:**
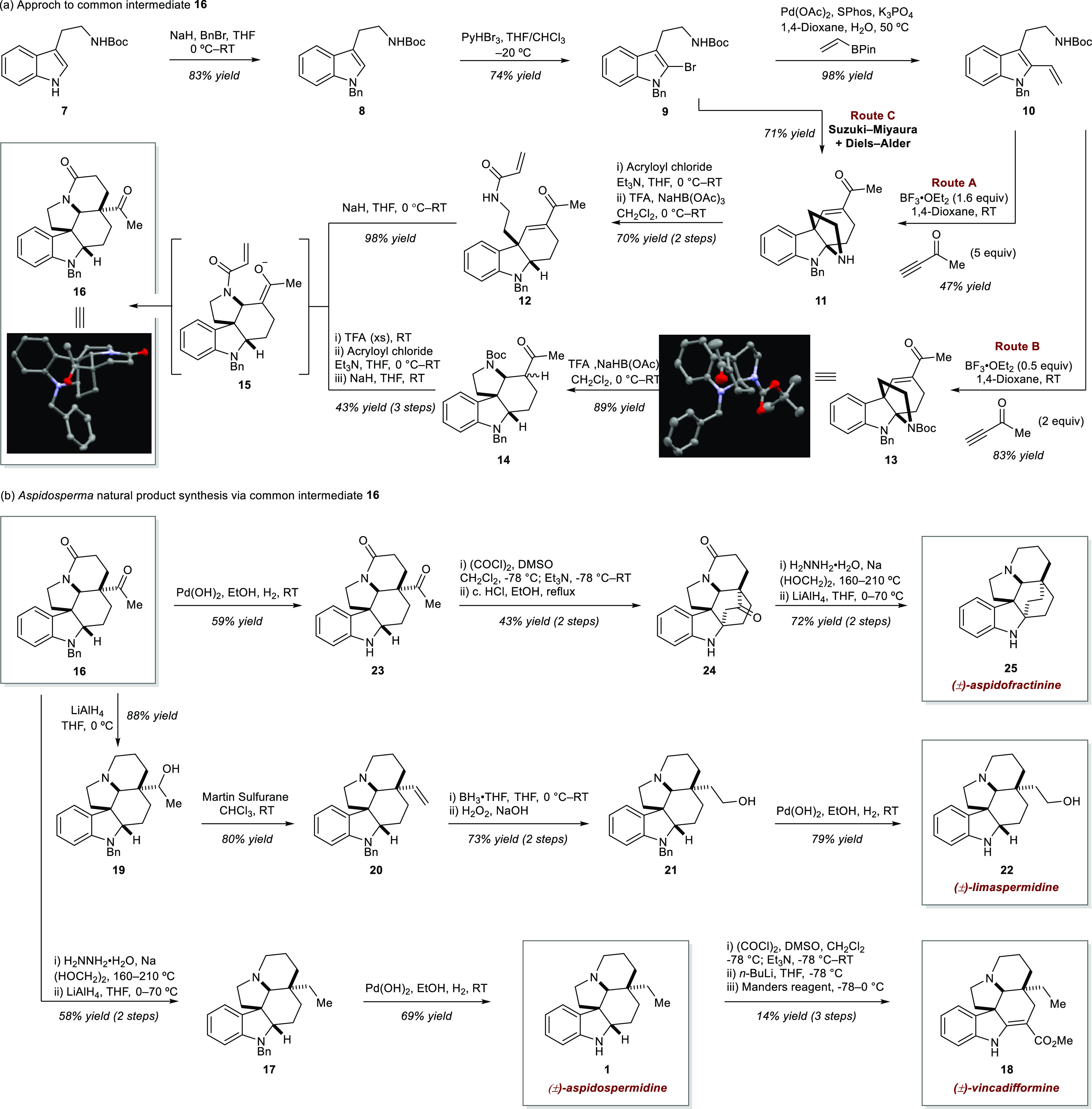
(a) Synthesis of Common Intermediate **16**; (b) *Aspidosperma* Alkaloid Natural Product Syntheses via **16**; Thermal Ellipsoids for Compounds **13** and **16** Shown at 50% Probability, with Hydrogens and Solvent Molecules Removed for Clarity

The forward synthesis (Scheme 3) began with the synthesis of the targeted common intermediate (Scheme 3a). Commercial *N*-Boc tryptamine (**7**) was benzyl-protected (**8**) and brominated to provide **9**. Suzuki–Miyaura cross-coupling of **9** with vinyl BPin delivered vinyl indole **10**. At this stage, two alternative sequences delivered access to the key common intermediate **16**. In route A, Diels–Alder reaction of **10** with butyn-2-one using excess BF_3_·OEt_2_ provided pyrroloindoline **11**, with concomitant Boc deprotection. This reaction proceeded in good yield, considering the overall number of the overall process involved. Treatment of **11** with acryloyl chloride and reduction of the generated iminium with NaHB(OAc)_3_ afforded acrylamide **12** in good yield over two steps.

Treatment of **12** with NaH induced an intramolecular aza-Michael reaction to form the E ring and generate enolate **15**, which underwent an intramolecular Michael addition to the acrylamide, forming the D ring, providing common intermediate **16**, and completing the carbon scaffold of (±)-aspidospermidine in almost quantitative yield.

The alternative route B involved a minor adjustment to the sequence of events for route A. Vinyl indole **10** underwent the same Diels–Alder reaction with butyn-2-one; however, lowering the stoichiometry of BF_3_·OEt_2_ provided pyrroloindoline **13**, which retained the Boc protecting group, and was a more efficient approach to a similar intermediate. Treatment of **13** with TFA and NaHB(OAc)_3_ induced skeletal rearrangement to forge the E ring by elimination of the tryptamine nitrogen, reduction of the generated iminium, and aza-Michael addition of the tryptamine to the C ring enone, affording compound **14** in good yield. Use of **14** in a three-step sequence comprising Boc deprotection using TFA, amidation with acryloyl chloride, and intramolecular Michael addition to forge the D ring delivered **16** in moderate yield. Overall, routes A and B were comparable in overall efficiency in accessing **16**.

Finally, it proved possible to access **11** in a telescoped process from **9** by combining the Suzuki–Miyaura and Diels–Alder events (route C). This delivered intermediate **11** in 71% and decreased the number of isolations required for the overall route. Compound **11** was then advanced to **16** using the same sequence of events as route A.

The Lewis acid-mediated Diels–Alder reaction suggested the possibility of an asymmetric process; however, attempts to induce enantioselectivity using chiral Lewis acids in this cascade were unsuccessful.

With access to **16**, completion of the synthesis of aspidospermidine was straightforward (Scheme 3b). Reduction of the ketone and lactone was achieved by telescoped Wolff–Kishner and LiAlH_4_ reductions, affording **17** in good yield. Hydrogenative debenzylation delivered (±)-aspidospermidine **1** (11 chemical transformations and 7 isolations). This also enabled access to (±)-vincadifformine (**18**) via a three-step process including Swern oxidation, deprotonation, and trapping of the generated enaminate with Mander’s reagent (14 chemical steps and 8 isolations).

In alignment with the design plan, the utility of the common intermediate **16** was then shown by elaboration to several other members of this class of natural products. Treatment of **16** with LiAlH_4_ afforded alcohol **19**, which was dehydrated with Martin sulfurane to afford alkene **20**. This allowed Brown hydroboration/oxidation to afford alcohol **21**, which was debenzylated to afford (±)-limaspermidine **22** (13 chemical steps and 9 isolations). Last, debenzylation of **16** afforded amine **23**, which underwent one-pot Swern oxidation and an intramolecular acid-promoted Mannich reaction to afford **24**. Reduction of the ketone and lactam using the Wolff–Kishner/LiAlH_4_ sequence then provided (±)-aspidofractinine **25** (13 steps and 8 isolations).

## Conclusions

In summary, a concise, divergent route to the *Aspidosperma* alkaloids has been developed based on a cascade and a common intermediate strategy. The main design element is a cascade cross-coupling/Diels–Alder process, which allows construction of the majority of the necessary ring system. A cascade aza-Michael/Michael reaction then completes the carbon skeleton and provides the key common intermediate, which can be readily elaborated to various members of this natural product family.

## Supporting Information Available

Characterization data, copies of ^1^H and ^13^C NMR spectra, and crystal structure data. The research data supporting this publication can be accessed at https://doi.org/10.17630/b4202d38-1f7c-4344-9816-efdb070d7d37 (PDF)

## References

[ref1] Saxton J. E. Chapter 9 Synthesis of the Aspidosperma Alkaloids. In The Alkaloids: Chemistry and Biology; Cordell G. A., Ed.; Academic Press: San Diego, CA, 1998; Vol. 50, pp 343–376.

[ref2] Lopchuk J. M. Recent Advances in the Synthesis of Aspidosperma-Type Alkaloids. In Progress in Heterocyclic Chemistry; GribbleJoule G. W. J. A., Ed.; Elsevier: Oxford, U.K., 2011; Vol. 23, pp 1–25.

[ref3] Saxton J. E. Chemistry of Heterocyclic Compounds; Wiley- Interscience: New York, 1983; Vol. 25, pp 331–437.

[ref4] Saxton J. E. Alkaloids of the Aspidospermine Group. In The Alkaloids: Chemistry and Biology; Cordell G. A., Ed.; Academic Press: San Diego, 1998; Vol. 51, pp 1–197.

[ref5] O’Connor S. E.; Maresh J. J. Chemistry and Biology of Monoterpeneindole Alkaloid Biosynthesis. Nat. Prod. Rep. 2006, 23, 532–547. 10.1039/b512615k. 16874388

[ref6] Biemann K.; Friedmann-Spiteller M.; Spiteller G. An Investigation by Mass Spectrometry of the Alkaloids of Aspidosperma Quebracho-blanco. Tetrahedron Lett. 1961, 2, 485–492. 10.1016/s0040-4039(00)71759-x.

[ref7] Biemann K.; Spiteller-Friedmann M.; Spiteller G. Application of Mass Spectrometry to Structure Problems. X.1 Alkaloids of the Bark of Aspidosperma quebracho blanco. J. Am. Chem. Soc. 1963, 85, 631–638. 10.1021/ja00888a034.

[ref8] aJones S. B.; Simmons B.; Mastracchio A.; MacMillan D. W. C. Collective Synthesis of Natural Products by Means of Organocascade Catalysis. Nature 2011, 475, 183–188. 10.1038/nature10232. 21753848 PMC3439143

[ref9] aWilson R. M.; Danishefsky S. J. Pattern Recognition in Retrosynthetic Analysis: Snapshots in Total Synthesis. J. Org. Chem. 2007, 72, 4293–4305. 10.1021/jo070871s. 17539594

[ref10] aWenkert E.; Liu S. Total Synthesis of (±)-Aspidofractinine and (±)-Aspidospermidine. J. Org. Chem. 1994, 59, 7677–7682. 10.1021/jo00104a023.

[ref11] aWhite K. L.; Movassaghi M. Concise Total Syntheses of (+)-Haplocidine and (+)-Haplocine via Late-Stage Oxidation of (+)-Fendleridine Derivatives. J. Am. Chem. Soc. 2016, 138, 11383–11389. 10.1021/jacs.6b07623. 27510728 PMC5014600

[ref12] aPandey G.; C P. Iminium Ion Cascade Reaction in the Total Synthesis of (+)-Vincadifformine. Org. Lett. 2011, 13, 4672–4675. 10.1021/ol201892j. 21815617

[ref13] Cain D. L.; McLaughlin C.; Molloy J. J.; Carpenter-Warren C.; Anderson N. A.; Watson A. J. B. A Cascade Suzuki-Miyaura/Diels-Alder Protocol: Exploring the Bifunctional Utility of Vinyl Bpin. Synlett 2019, 30, 787–791. 10.1055/s-0037-1611228.

[ref14] Molloy J. J.; Seath C. P.; West M. J.; McLaughlin C.; Fazakerley N. J.; Kennedy A. R.; Nelson D. J.; Watson A. J. B. Interrogating Pd(II) Anion Metathesis Using a Bifunctional Chemical Probe: A Transmetalation Switch. J. Am. Chem. Soc. 2018, 140, 126–130. 10.1021/jacs.7b11180. 29257859

