# Supplementary material for: Total Synthesis of (±)-Aspidospermidine, (±)-Aspidofractinine, (±)-Limaspermidine, and (±)-Vincadifformine via a Cascade and Common Intermediate Strategy
Source: J Org Chem. 2022 Oct 19;87(22):15559–63. doi: 10.1021/acs.joc.2c02099 (PMC9680024; doi:10.1021/acs.joc.2c02099)
Supplement: Supplementary file 1 — jo2c02099_si_001.pdf [file jo2c02099_si_001.pdf]

Total synthesis of (±)-aspidospermidine, (±)-aspidofractinine, (±)-limaspermidine, and (±)-vincadifformine via a cascade and common intermediate strategy

Supporting Information

David L. Cain,[a] Niall A. Anderson,[b] David B. Cordes,[a] Alexandra M. Z. Slawin,[a] and Allan J. B. Watson[a]*

[a] EaStCHEM, School of Chemistry, University of St Andrews, North Haugh, St Andrews, Fife, KY16 9ST, U.K.

[b] GlaxoSmithKline, Medicines Research Centre, Gunnels Wood Road, Stevenage, SG1 2NY, U.K.

*Email: aw260@st-andrews.ac.uk

Contents

1. General Experimental Details
2. Experimental Procedures and Characterization Data
3. X-Ray Crystallography Data
4. References
5. ^{1}H and ^{13}C NMR Spectra

1. General Experimental Details

1.1 Purification of Solvents & Reagents

All reagents and solvents were obtained from commercial suppliers and were used without further purification unless otherwise stated. Purification was carried out according to standard laboratory methods.[1] All solvents used for dry reactions (PhMe, CH_2Cl_2, THF, Et_2O) were obtained from a PureSolv SPS-400-5 solvent purification system and stored in a septum-sealed oven-dried flask over previously activated 4 Å molecular sieves under nitrogen. Dry 1,4-dioxane was obtained by distillation over $LiAlH_4$ and stored in a septum-sealed oven-dried flask over previously activated 4 Å molecular sieves under nitrogen. Dry $CHCl_3$ was obtained by washing out residual amounts of EtOH with water, drying the organics with K_2CO_3 and then subsequently distilling organics over $CaCl_2$. The distilled solvent was stored in a septum-sealed oven-dried flask over previously activated 4 Å molecular sieves under nitrogen. The flask was then covered in aluminium foil. Dry Et_3N was obtained by distillation over CaH and stored in a septum-sealed oven-dried flask over previously activated 4 Å molecular sieves under nitrogen. The flask was then covered in aluminium foil. EtOAc, Et_2O, MeOH, CH_2Cl_2, hexane, and petroleum ether 40–60°C for purification purposes were used as obtained from suppliers without further purification. Inorganic bases were dried in a Heraeus Vacutherm oven at 60 °C under vacuum for a minimum of 24 hours before use.

1.2 Experimental Details

Reactions were carried out using conventional glassware (preparation of intermediates) or in capped 5 or 20 mL microwave vials. Microwave vials were oven-dried (150 °C) and cooled to room temperature under vacuum and backfilled with N_2 prior to use. Reaction mixtures were prepared in a microwave vial before being capped with a septum and purged using N_2/vacuum (three cycles). Room temperature was generally *ca.* 20 ºC. Reactions were carried out at elevated temperatures in a sand bath atop a temperature-regulated hotplate/stirrer. Cooling to 0 °C was achieved using an ice/water bath. Cooling to –78 °C and –60 ºC was achieved using a dry ice/acetone bath.

1.3 Purification of Products

Thin layer chromatography was carried out using Merck silica plates coated with fluorescent indicator UV254. These were analysed under 254 nm UV light and/or developed using potassium permanganate or vanillin solution. Normal phase flash chromatography was carried out using ZEOprep 60 HYD 40-63 µm silica gel.

1.4 Analysis of Products

Fourier Transformed Infra-Red (FTIR) spectra were obtained on a Shimadzu IRAffinity-1 Fourier transform IR spectrophotometer fitted with a Specac Quest ATR accessory at St Andrews University. Spectra were recorded of either thin films or solids, with characteristic absorption wavenumbers (υmax) reported in cm^{-1}. ^{19}F NMR spectra were obtained on either a Bruker AV 400 spectrometer at 376 MHz or Bruker AV 500 at 470 MHz. ^{1}H and ^{13}C NMR spectra were obtained on either a Bruker AV 400 at 400 MHz and 125 MHz, Bruker AV 500 at 500 MHz and 126 MHz, or Bruker AV 700 at 700 MHz and 127 MHz. Chemical shifts are reported in ppm and coupling constants are reported in Hz: $CDCl_3$ referenced at 7.26 (^{1}H) and 77.2 ppm (^{13}C); CD_3CN referenced at 1.94 (^{1}H) and 118.26 ppm (^{13}C). Coupling constants throughout the experimental section were reported as observed in spectra without corrections. High-resolution mass spectra were obtained through analysis at the University of St Andrews mass spectrometry facility.

2. Experimental Procedures and Characterization Data

Compound 1

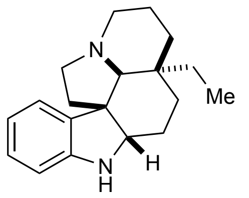

An oven dried round bottom flask was charged with stir bar, Pearlman's catalyst (20% $Pd(OH)_2/C$; 544 mg, 0.77 mmol, 1.85 equiv), and compound **17** (156 mg, 0.42 mmol, 1 equiv). The flask was then sealed and purged with N_2. EtOH (14 mL) was added and the reaction mixture was sparged with H_2. The mixture was then stirred at room temperature for 26 h under balloon of H_2. After the reaction was complete, the vessel was purged with N_2 before filtering the mixture through a pad of Celite®. The filtrate was concentrated to a residue, which was purified by column chromatography (0–10% MeOH in CH_2Cl_2) to afford the product as a colourless wax. The product was further purified by acid-base extraction: The residue was dissolved in 2 M HCl (20 mL) and extracted with CH_2Cl_2 (2 × 20 mL; discarded). The aqueous phase was then basified with 2 M NaOH solution (25 mL) and extracted with CH_2Cl_2 (2 × 20 mL). The combined organics were dried with Na_2SO_4, filtered, and concentrated under reduced pressure, to give a colourless wax (81.9 mg, 69%).

υ_{max} (film): 3318, 3298, 2934, 2828, 1607, 1462 cm^{-1}.

^{1}H NMR (400 MHz, $CDCl_3$): δ 7.08 (dd, *J* = 7.4, 1.3 Hz, 1H), 7.01 (td, *J* = 7.6, 1.3 Hz, 1H), 6.73 (td, *J* = 7.4, 1.0 Hz, 1H), 6.64 (dt, *J* = 7.7, 0.8 Hz, 1H), 3.51 (dd, *J* = 11.1, 6.2 Hz, 1H), 3.16 – 3.09 (m, 1H), 3.06 (ddt, *J* = 10.9, 4.0, 1.8 Hz, 1H), 2.35 – 2.19 (m, 3H), 2.00 – 1.89 (m, 2H), 1.81 – 1.69 (m, 1H), 1.67 – 1.59 (m, 2H), 1.55 – 1.43 (m, 3H), 1.42 – 1.33 (m, 1H), 1.13 (dd, *J* = 13.5, 4.6 Hz, 1H), 1.09 – 1.02 (m, 1H), 0.94 – 0.80 (m, 1H), 0.63 (t, *J* = 7.5 Hz, 3H). Indoline proton not observed.

^{13}C NMR (126 MHz, $CDCl_3$): δ 149.5, 135.9, 127.2, 123.0, 119.1, 110.5, 71.4, 65.8, 54.0, 53.5, 53.2, 39.0, 35.8, 34.6, 30.1, 28.2, 23.1, 21.9, 7.0.

HRMS: Exact mass calculated for $[M+H]^+$ ($C_{19}H_{27}N_2$) requires *m/z* 283.2169, found *m/z* 283.2161.

Data in agreement with that reported in the literature.[2]

Compound 7

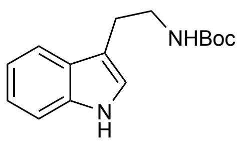

An oven-dried flask with stir bar and N_2 line was purged and sealed with a septum before charging with tryptamine (500 mg, 1 equiv, 3.13 mmol) under N_2 flow, and 1,4-dioxane (5.2 mL) and Et_3N (870 µL, 2 equiv, 6.26 mmol) via syringe. The mixture was stirred for 15 min at room temperature before addition of Boc_2O (750 mg, 1.1 equiv, 3.44 mmol) via syringe. The reaction mixture was stirred for 5 h at room temperature under N_2. After the reaction was complete, the mixture was concentrated under reduced pressure to give a crude residue, which was purified by column chromatography (silica gel, 5–50% EtOAc in petroleum ether 40–60°) to afford the product as light yellow oil, which solidified upon standing (820 mg, >99%).

^{1}H NMR (400 MHz, $CDCl_3$): δ 8.11 (s, 1H), 7.61 (dd, *J* = 7.9, 1.1 Hz, 1H), 7.37 (dt, *J* = 8.1, 0.9 Hz, 1H), 7.21 (ddd, *J* = 8.2, 7.0, 1.2 Hz, 1H), 7.13 (ddd, *J* = 8.0, 7.1, 1.1 Hz, 1H), 7.03 (s, 1H), 4.62 (s, 1H), 3.53 – 3.39 (m, 2H), 2.96 (t, *J* = 6.8 Hz, 2H), 1.44 (s, 9H).

^{13}C NMR (126 MHz, $CDCl_3$): δ 156.1, 136.5, 127.5, 122.3, 122.2, 119.5, 119.0, 113.3, 111.3, 79.3, 40.9, 28.6, 25.9.

Spectroscopic data were in agreement with literature values.[3]

Compound 8

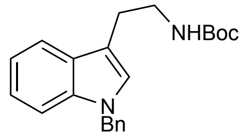

An oven-dried flask with stir bar was charged with NaH (184 mg, 4.61 mmol, 1.2 equiv). The flask was sealed with a septum and N_2 line fitted before addition of THF (7.6 mL) via syringe. The mixture was cooled to 0 °C before slow addition of a solution of compound **7** (1 g, 3.84 mmol, 1 equiv) in THF (7.6 mL) via syringe. The resulting mixture was stirred for 30 min at 0 °C before dropwise addition of benzyl bromide (0.68 mL, 5.76 mmol, 1.5 equiv) via syringe. The mixture was stirred for 5 min then allowed to warm to room temperature where it was stirred for 3 h. After the reaction was complete, water (20 mL) was added, and the organics were extracted with EtOAc (3 × 20 mL). The combined organics were dried with Na_2SO_4, filtered, and concentrated under reduced pressure. The crude product was purified by column chromatography (silica gel, 0–10% EtOAc in petroleum ether 40–60°) to afford the product as pale yellow oil (1.11 g, 83%).

^{1}H NMR (400 MHz, $CDCl_3$): δ 7.61 (dt, *J* = 7.7, 1.0 Hz, 1H), 7.32 – 7.25 (m, 4H), 7.18 (ddd, *J* = 8.2, 6.9, 1.2 Hz, 1H), 7.11 (dddd, *J* = 6.9, 5.2, 3.1, 1.1 Hz, 3H), 6.96 (s, 1H), 5.28 (s, 2H), 4.59 (s, 1H), 3.46 (q, *J* = 6.9 Hz, 2H), 2.96 (t, *J* = 6.9 Hz, 2H), 1.43 (s, 9H).

^{13}C NMR (126 MHz, $CDCl_3$): δ 156.1, 137.7, 136.9, 128.9, 128.2, 127.7, 126.9, 126.3, 122.0, 119.3, 119.2, 112.5, 109.8, 79.2, 50.0, 41.0, 28.5, 25.9.

Spectroscopic data were in agreement with literature values.[4]

Compound 9

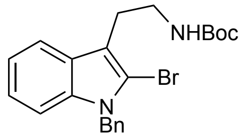

An oven-dried 2-necked flask with stir bar was charged with compound **8** (350 mg, 1 mmol, 1 equiv). The flask was sealed with a septum and fitted with N_2 line before charging with THF:$CHCl_3$ (1:1, 10 mL) via syringe. The solution was cooled to –20 °C and stirred for 15 mins. Pyridinium tribromide (recrystallized; 390 mg, 1.54 mmol, 1.22 equiv) was then added portion-wise under N_2 flow over 1 h at –20 °C. The reaction mixture was stirred for 20 min at –20 ºC then quenched with sat. aq. Na_2SO_3 solution (20 mL), and following a colour change to light yellow, sat. aq. $NaHCO_3$ solution (20 mL) was added. The mixture was extracted with CH_2Cl_2 (3 × 40 mL), and the combined organic phases were dried over Na_2SO_4, filtered, and concentrated under reduced pressure. The crude residue was purified by column chromatography (0–10% EtOAc in petroleum ether) to afford the desired product as a white solid (317 mg, 74%).

υ_{max} (film): 3429, 3350, 2974, 2928, 1695, 1506, 1497, 1452, 1363, 1331, 1159 cm^{-1}.

^{1}H NMR (400 MHz, $CDCl_3$): δ 7.58 (d, *J* = 7.5 Hz, 1H), 7.31 – 7.20 (m, 4H), 7.17 – 7.10 (m, 2H), 7.08 (ddt, *J* = 7.3, 1.5, 0.8 Hz, 2H), 5.41 (s, 2H), 4.59 (s, 1H), 3.41 (t, *J* = 6.0 Hz, 2H), 3.00 (t, *J* = 6.7 Hz, 2H), 1.43 (s, 9H).

^{13}C NMR (126 MHz, $CDCl_3$): δ 156.1, 137.3, 136.8, 128.9, 127.7, 127.6, 126.6, 122.3, 120.2, 118.5, 113.8, 112.5, 110.0, 79.2, 48.5, 40.6, 28.6, 26.2.

HRMS: Exact mass calculated for $[M+Na]^+$ ($C_{22}H_{25}O_2N_2{}^{79}BrNa$) requires *m/z* 451.0992, found *m/z* 451.0987.

Compound 10

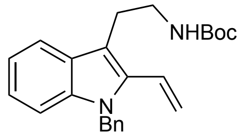

An oven-dried 20 mL microwave vial with stir bar was charged with $Pd(OAc)_2$ (17.9 mg, 0.08 mmol, 4 mol%), SPhos (65.5 mg, 0.16 mmol, 8 mol%), compound **9** (856 mg, 2 mmol, 1 equiv), vinyl BPin (338 mg, 2.19

mmol, 1.1 equiv), and K_3PO_4 (1268 mg, 6 mmol, 3 equiv). The vial was then capped and purged with N_2 fitted before the addition of 1,4-dioxane (8 mL) and H_2O (180 μL, 10 mmol, 5 equiv). The reaction mixture was heated to 50 °C and stirred for 24 h. After the reaction was complete, the reaction mixture was allowed to cool to room temperature, vented, and de-capped. The mixture was diluted in EtOAc (40 mL) and passed through a layer of Celite®. The filtrate was concentrated under reduced pressure to give a crude residue, which was purified by column chromatography (silica gel, 0–10% EtOAc in petroleum ether 40–60°) to afford the desired product as a beige solid (738 mg, 98%).

ν_{max} (film): 3350, 2976, 2931, 1694, 1504, 1496, 1452, 1366, 1263, 1248, 1165 cm^{-1}.

^{1}H NMR (500 MHz, $CDCl_3$): δ 7.63 (d, *J* = 7.8 Hz, 1H), 7.28 (dd, *J* = 8.1, 6.4 Hz, 2H), 7.25 – 7.19 (m, 2H), 7.19 – 7.15 (m, 1H), 7.12 (td, *J* = 7.2, 6.6, 1.4 Hz, 1H), 7.05 – 7.02 (m, 2H), 6.71 (dd, *J* = 17.9, 11.8 Hz, 1H), 5.53 (d, *J* = 17.8 Hz, 1H), 5.43 (dd, *J* = 11.8, 1.2 Hz, 1H), 5.39 (s, 2H), 4.63 (s, 1H), 3.45 (q, *J* = 6.8 Hz, 2H), 3.09 (t, *J* = 7.0 Hz, 2H), 1.45 (s, 9H).

^{13}C NMR (126 MHz, $CDCl_3$): δ 156.1, 138.1, 137.5, 135.2, 128.9, 128.2, 127.4, 126.1, 125.8, 122.7, 119.8, 119.2, 119.0, 111.8, 109.8, 79.2, 47.5, 41.4, 28.6, 25.6.

HRMS: Exact mass calculated for $[M+Na]^+$ ($C_{24}H_{28}O_2N_2Na$) requires *m/z* 399.2043, found *m/z* 399.2036.

Compound 11

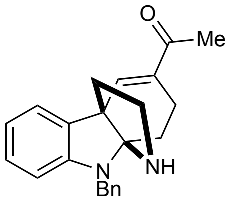

An oven-dried 20 mL MW vial (20 mL) with stir bar was charged with compound **10** (470 mg, 1.25 mmol, 1 equiv). The vial was then capped and purged and N_2 line fitted before addition of 3-butyn-2-one (488 μL, 6.25 mmol, 5 equiv), 1,4-dioxane (5 mL), and BF_3•OEt_2 (246 μL, 2 mmol, 1.6 equiv) sequentially via syringe. The reaction was then stirred at room temperature for 16 h. After the reaction was complete, the vial was vented and de-capped. MeOH (10 mL) was added and the mixture was concentrated under reduced pressure. The residue was treated with sat. aq. $NaHCO_3$ solution (15 mL) and extracted with EtOAc (3 × 15 mL). The combined organics were dried with Na_2SO_4, filtered, and concentrated under reduced pressure to give a crude residue, which was purified by column chromatography (silica gel, 0–30% EtOAc in CH_2Cl_2) to afford the desired product as a white solid (200 mg, 47%).

Cascade process from compound 9: An oven-dried 5 mL microwave vial with stir bar was charged with $Pd(OAc)_2$ (2.2 mg, 0.01 mmol, 4 mol%), SPhos (8.2 mg, 0.02 mmol, 8 mol%), compound **9** (107 mg, 0.25 mmol, 1 equiv), vinyl BPin (42.2 mg, 0.27 mmol, 1.1 equiv), and K_3PO_4 (159 mg, 0.75 mmol, 3 equiv). The vial was then capped and purged with N_2 fitted before the addition of 1,4-dioxane (1 mL) and H_2O (22.5 μL, 1.25 mmol, 5 equiv). The reaction mixture was heated to 50 °C and stirred for 24 h. After the reaction was complete, the reaction mixture was allowed to cool to room temperature, vented, and de-capped. The mixture was filtered to remove solid, eluting with 1,4-dioxane (1 mL). The filtrate was then treated with 3-butyn-2-one (39 μL, 2 equiv) and BF_3•OEt_2 (77 μL, 0.675 mmol, 2.5 equiv). The reaction was stirred for 4 h at room temperature, before analysis by ^{1}H NMR against a known internal standard (1,4-dinitrobenzene), revealing 71% conversion to desired product.

ν_{max} (film): 2928, 2870, 2357, 2342, 1667, 1601, 1489, 1354, 1069, 1030 cm^{-1}.

^{1}H NMR (400 MHz, $CDCl_3$): δ 7.35 – 7.29 (m, 4H), 7.27 – 7.22 (m, 1H), 7.14 (dd, *J* = 7.3, 1.3 Hz, 1H), 7.10 (d, *J* = 1.5 Hz, 1H), 7.00 (td, *J* = 7.7, 1.3 Hz, 1H), 6.68 (td, *J* = 7.4, 1.0 Hz, 1H), 6.22 (dd, 1H), 4.54 (d, *J* = 16.7 Hz, 1H), 4.32 (d, *J* = 16.8 Hz, 1H), 3.09 (ddd, *J* = 11.1, 7.2, 2.1 Hz, 1H), 2.85 (td, *J* = 10.8, 5.7 Hz, 1H), 2.39 (ddd, *J* = 12.0, 5.7, 2.1 Hz, 1H), 2.34 (s, 3H), 2.33 – 2.27 (m, 2H), 2.09 – 1.97 (m, 2H), 1.73 (ddd, *J* = 13.5, 8.2, 5.4 Hz, 1H). Amine proton not observed.

^{13}C NMR (126 MHz, $CDCl_3$): δ 198.8, 150.9, 142.8, 139.6, 137.9, 131.6, 128.7, 128.7, 126.9, 126.8, 123.0, 117.6, 106.9, 90.7, 56.8, 47.3, 44.8, 42.4, 31.0, 25.4, 20.1.

HRMS: Exact mass calculated for $[M+H]^+$ ($C_{23}H_{25}ON_2$) requires *m/z* 345.1961 , found *m/z* 345.1956.

Compound 12

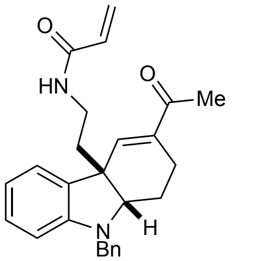

An oven-dried flask with stir bar was sealed and purged and N_2 line fitted before charging with a solution of compound **11** (141 mg, 0.41 mmol, 1 equiv) in THF (8.2 mL) followed by Et_3N (74 µL, 0.53 mmol, 1.3 equiv). The resulting mixture was cooled to 0 °C. Acryloyl chloride (44.5 µL, 0.53 mmol, 1.3 equiv) was then added dropwise *via* syringe. Then reaction mixture was stirred and allowed to warm to room temperature, then stirred for 3 h. The reaction mixture was then cooled to 0 °C, before TFA (94 µL, 1.23 mmol, 3 equiv) was added *via* syringe. After 25 min, under N_2 flow, the flask was opened and $NaHB(OAc)_3$ (347 mg, 1.64 mmol, 4 equiv) was added. The flask was sealed and N_2 line fitted, and the reaction mixture was allowed to warm to room temperature and stirred stir for 24 h. After the reaction was complete, sat. aq. $NaHCO_3$ solution (20 mL) was added, and the mixture was extracted with EtOAc (3 × 20 mL). The combined organics were dried with Na_2SO_4, filtered, and concentrated under reduced pressure. The crude residue was purified by column chromatography (0–30% EtOAc in CH_2Cl_2) to afford the product as a pale yellow oil (114 mg, 70%).

υ_{max} (film): 3281, 3061, 2926, 2855, 2361, 2342, 1655, 1601, 1483, 1263, 957 cm^{-1}.

^{1}H NMR (500 MHz, $CDCl_3$): δ 7.37 – 7.23 (m, 5H), 7.13 (dd, *J* = 7.3, 1.3 Hz, 1H), 7.08 (td, *J* = 7.7, 1.3 Hz, 1H), 6.73 (td, *J* = 7.4, 1.0 Hz, 1H), 6.62 (d, *J* = 1.5 Hz, 1H), 6.44 (d, *J* = 7.8 Hz, 1H), 6.25 (dd, *J* = 16.9, 1.4 Hz, 1H), 5.99 (dd, *J* = 16.9, 10.3 Hz, 1H), 5.64 (dd, *J* = 10.4, 1.4 Hz, 1H), 5.60 (d, *J* = 6.2 Hz, 1H), 4.46 (d, *J* = 16.0 Hz, 1H), 4.21 (d, *J* = 16.0 Hz, 1H), 3.79 (dd, *J* = 4.7, 3.0 Hz, 1H), 3.43 – 3.31 (m, 2H), 2.29 (s, 3H), 2.29 – 2.25 (m, 2H), 2.20 – 1.99 (m, 3H), 1.70 (dddd, *J* = 14.3, 9.4, 6.6, 3.1 Hz).

^{13}C NMR (126 MHz, $CDCl_3$): δ 199.1, 165.6, 151.4, 142.8, 138.6, 138.3, 132.0, 130.7, 128.9, 128.8, 127.4, 127.3, 126.7, 123.2, 118.2, 108.2, 66.0, 50.6, 47.9, 38.0, 36.2, 25.5, 22.3, 18.2.

HRMS: Exact mass calculated for $[M+Na]^+$ ($C_{26}H_{28}O_2N_2Na$) requires *m/z* 423.2043, found *m/z* 423.2034.

Compound 13

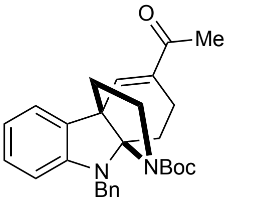

An oven-dried 20 mL MW vial was charged with compound **10** (752 mg, 2 mmol, 1 equiv). The vial was then capped and purged with N_2 line fitted. 1,4-Dioxane (8 mL) was added by syringe before 3-butyn-2-one (0.31 mL, 4 mmol, 2 equiv) and BF_3●OEt_2 (123 µL, 1 mmol, 0.5 equiv) were added sequentially via syringe. The reaction was then stirred at room temperature for 4 h. After the reaction was complete the reaction mixture was vented and de-capped. The mixture was treated with MeOH (25 mL) and concentrated. The residue was then treated with sat. aq. $NaHCO_3$ solution (30 mL) and the organics were extracted with EtOAc (3 × 30 mL). The combined organics were dried with Na_2SO_4, filtered, and concentrated under reduced pressure. The crude residue was purified by column chromatography (silica gel, 0–10% EtOAc in hexane) to afford the desired product as a mixture of rotamers as a white solid (735 mg, 83%).

υ_{max} (film): 2974, 2930, 1690, 1670, 1485, 1366, 1161, 1130 cm^{-1}.

^{1}H NMR (500 MHz, CD_3CN, 75 °C): δ 7.32 – 7.18 (m, 6H), 7.00 (t, *J* = 1.5 Hz, 1H), 6.96 (td, *J* = 7.7, 1.2 Hz, 1H), 6.67 (td, *J* = 7.5, 0.9 Hz, 1H), 6.10 (d, *J* = 7.9 Hz, 1H), 4.99 (d, *J* = 16.9 Hz, 1H), 4.58 (d, *J* = 16.9 Hz,

1H), 3.64 – 3.58 (m, 1H), 3.34 (dt, *J* = 10.8, 7.1 Hz, 1H), 2.56 – 2.45 (m, 2H), 2.35 (dt, *J* = 12.7, 6.4 Hz, 1H), 2.29 (s, 3H), 2.25 – 2.11 (m, 3H), 1.37 (s, 9H).

^{13}C NMR (126 MHz, CD_3CN, 75 °C): δ 199.6, 155.4, 150.1, 141.3, 140.6, 139.7, 132.4, 129.9, 129.7, 127.9, 127.8, 123.8, 119.0, 108.6, 91.2, 81.1, 59.6, 49.0, 48.3, 36.6, 29.0, 27.3, 26.1, 21.4.

HRMS (ESI): Exact mass calculated for $[M+Na]^+$ ($C_{28}H_{32}O_3N_2Na$) requires *m/z* 467.2305, found *m/z* 467.2309.

Compound 14

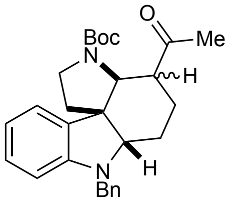

An oven-dried 5 mL MW vial with stir bar was charged with compound **13** (111 mg, 0.25 mmol, 1 equiv) and $NaHB(OAc)_3$ (66.1 mg, 0.31 mmol, 1.25 equiv). The vial was then sealed and purged with N_2 line fitted before addition of CH_2Cl_2 (5 mL). The mixture was cooled to 0 °C and stirred for 15 min before addition of TFA (76.5 µL, 1 mmol, 4 equiv) *via* syringe. The reaction was stirred and allowed to reach room temperature and stir for 6 h. After the reaction was complete, the vial was vented and de-capped. The mixture was then quenched with sat. aq. $NaHCO_3$ solution (30 mL) and extracted with CH_2Cl_2 (3×25 mL). The combined organics were dried with Na_2SO_4, filtered, and concentrated under reduced pressure. The crude residue was purified by column chromatography (silica gel, 0–40% EtOAc in hexane) to yield the product as pale yellow oil (99.3 mg, 89%) as an inconsequential mixture of diastereomers (0.62:0.38 ratio).

υ_{max} (film): 2972, 2361, 1686, 1603, 1391, 1364, 1167, 1126 cm^{-1}.

^{1}H NMR (700 MHz, $CDCl_3$; Major diastereomer assigned unless specified otherwise) δ 7.39 – 7.31 (m, 4H), 7.28 – 7.25 (m, 1H), 7.09 – 7.03 (m, 1H), 6.90 (dd, *J* = 7.4, 1.3 Hz, 0.37H, minor), 6.87 (dd, *J* = 7.3, 1.3 Hz, 0.63H), 6.72 – 6.67 (m, 1H), 6.53 (d, *J* = 7.9 Hz, 0.6H), 6.46 (d, *J* = 7.9 Hz, 0.37H, minor), 4.43 (t, *J* = 17.1 Hz, 1H), 4.16 (d, *J* = 16.2 Hz, 1H), 4.07 (dt, *J* = 11.9, 9.2 Hz, 0.64H), 3.86 (dt, *J* = 11.8, 9.2 Hz, 0.39H, minor), 3.83 (d, *J* = 10.9 Hz, 0.38H, minor), 3.59 – 3.50 (m, 2H), 3.46 (t, *J* = 3.0 Hz, 0.39H, minor), 3.44 (t, *J* = 3.3 Hz, 0.62H), 2.37 (ddd, *J* = 12.7, 10.9, 2.5 Hz, 0.62H), 2.29 – 2.21 (m, 1.12H), 2.19 (s, 1.27H), 2.16 (s, 1.83H), 2.15 – 2.10 (m, 0.56H), 2.10 – 2.04 (m, 1H), 1.88 (qt, *J* = 13.5, 1.9 Hz, 1H), 1.59 – 1.49 (m, 2H), 1.44 (s, 3.37H, minor), 1.31 (s, 5.63H).

^{13}C NMR (126 MHz, $CDCl_3$; Mixture of diastereomers – unassigned): δ 210.6, 210.2, 155.0, 154.5, 151.2, 151.1, 138.7, 138.6, 136.2, 136.1, 128.7, 128.4, 128.3, 127.5, 127.2, 127.2, 121.8, 121.7, 119.2, 119.1, 108.7, 80.6, 79.7, 67.1, 66.8, 65.0, 63.5, 55.2, 52.8, 52.6, 51.8, 51.7, 51.5, 43.8, 43.2, 32.3, 31.8, 31.7, 30.9, 28.5, 28.3, 27.5, 22.7, 22.4, 22.1, 21.8.

HRMS: Exact mass calculated for $[M+H]^+$ ($C_{28}H_{35}O_3N_2$) requires *m/z* 447.2642, found *m/z* 447.2626.

Compound 16

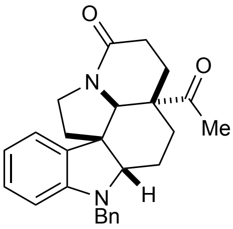

From compound **12**: An oven-dried 5 mL MW vial was charged with compound **12** (15.6 mg, 0.04 mmol, 1 equiv). The vial was sealed with a septum and purged with N_2 line fitted. THF (1 mL) was added, and the mixture cooled to 0 °C. NaH (3.1 mg, 0.08 mmol, 2 equiv) was added under N_2 flow. After 30 min, the reaction

was allowed to warm to room temperature and stirred for a further 1.5 h. After the reaction was complete, the mixture was treated with sat. aq. $NaHCO_3$ solution (20 mL) and extracted with EtOAc (3 × 20 mL). The combined organics were dried with Na_2SO_4, filtered, and concentrated under reduced pressure to yield the product as a yellow solid (15.3 mg, 98%). Data as reported below.

From compound **14**: An oven-dried flask with stir bar was charged with compound **14** (223 mg, 0.5 mmol, 1 equiv). The flask was then sealed and purged with N_2 line fitted before addition of TFA (0.96 mL, 12.5 mmol, 25 equiv) via syringe. The reaction mixture was stirred at room temperature for 1.5 h. The vial was vented and decapped before the mixture was concentrated under reduced pressure and azeotroping with PhMe (3 × 20 mL) in the same flask. The flask was sealed and purged with N_2 line fitted before dissolving the residue in THF (10 mL). Et_3N (0.22 mL. 1.55 mmol, 3.1 equiv) was added and the mixture was cooled to 0 °C and stirred. After 15 min, acryloyl chloride (62.7 µL, 0.75 mmol, 1.5 equiv) was added dropwise via syringe. The reaction was allowed to reach room temperature and was stirred for a further 3 h. After the reaction was complete, sat. aq. $NaHCO_3$ solution (20 mL) was added, and the mixture was extracted with EtOAc (3 × 20 mL). The combined organics were dried with Na_2SO_4, filtered, and concentrated under reduced pressure. The resulting crude residue was carried forward without further purification.

A stir bar was added to the flask containing the residue and the flask was sealed with a septum and purged with N_2 line fitted. The residue was dissolved in THF (5 mL), cooled to 0 °C, and stirred. After 15 min, NaH (200 mg, 5 mmol, 10 equiv) was added portionwise under N_2 flow. The flask was sealed and stirred for 10 min before allowing to warm to room temperature and stir for 14 h. After the reaction was complete, the mixture was cooled to 0 °C and quenched with water (30 mL). The mixture was allowed to reach room temperature then extracted with CH_2Cl_2 (3 × 30 mL). The combined organics were then washed once with brine (30 mL). The organics were dried with Na_2SO_4, filtered, and concentrated under reduced pressure. The crude residue was purified by column chromatography (silica gel, 0–50% MeCN in CH_2Cl_2) to afford the desired product as a beige solid (86.9 mg, 43%).

υ_{max} (film): 3049, 2930, 2872, 2361, 2342, 1701, 1616, 1605, 1479, 1452, 1352 cm^{-1}.

^{1}H NMR (500 MHz, $CDCl_3$): δ 7.37 – 7.27 (m, 5H), 7.14 (dd, *J* = 7.4, 1.2 Hz, 1H), 7.04 (td, *J* = 7.6, 1.3 Hz, 1H), 6.71 (t, *J* = 7.4 Hz, 1H), 6.37 (d, *J* = 7.9 Hz, 1H), 4.73 (s, 1H), 4.45 (d, *J* = 14.9 Hz, 1H), 4.07 (d, *J* = 14.9 Hz, 1H), 3.65 (dd, *J* = 12.6, 9.6 Hz, 1H), 3.48 (td, *J* = 11.9, 7.6 Hz, 1H), 3.14 (dd, *J* = 10.1, 5.1 Hz, 1H), 2.47 (ddd, *J* = 18.2, 7.1, 2.8 Hz, 1H), 2.42 – 2.29 (m, 2H), 2.06 (s, 3H), 1.97 – 1.90 (m, 1H), 1.90 – 1.86 (m, 1H), 1.82 (dtd, *J* = 13.1, 4.9, 2.7 Hz, 1H), 1.76 – 1.67 (m, 2H), 1.42 (td, *J* = 13.6, 2.8 Hz, 1H), 1.30 (ddd, *J* = 13.2, 9.9, 2.9 Hz, 1H).

^{13}C NMR (126 MHz, $CDCl_3$): δ 208.7, 168.4, 150.0, 138.0, 129.4, 128.8, 128.6, 127.7, 127.5, 124.3, 118.1, 107.3, 63.9, 60.4, 54.3, 49.9, 48.6, 43.2, 35.5, 31.1, 27.9, 25.3, 22.5, 22.2.

HRMS: Exact mass calculated for $[M+H]^+$ ($C_{26}H_{29}O_2N_2$) requires *m/z* 401.2224, found *m/z* 401.2216.

Compound 17

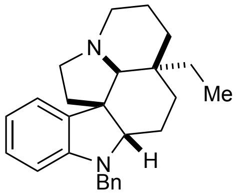

An oven-dried round bottomed flask with stir bar and condenser was purged and sealed with N_2 line fitted before charging with a solution of compound **16** (290 mg, 0.72 mmol, 1 equiv), Na (2.9 g, 126 mmol, 174 equiv), and hydrazine monohydrate (9 mL, 185 mmol, 256 equiv) in ethylene glycol (72.4 mL). The mixture was heated at 160 °C for 1 h, 190 °C for 2.5 h, and 210 °C for 18 h. The resulting mixture was allowed to cool to room temperature before quenching with water (75 mL) and extracted with EtOAc (5 × 50 mL). The combined organic extracts were washed with brine (5 × 50 mL), dried with Na_2SO_4, filtered, and concentrated under reduced pressure, to give a pale yellow oil crude residue, which was carried through to the next step without further purification.

An oven-dried flask with stir bar was sealed and purged with N_2 line fitted and charged with a solution of the crude residue in THF (29 mL) was added and subsequently cooled to 0 °C. $LiAlH_4$ (578 mg, 14.5 mmol, 20

equiv) was then added under N_2 flow. The mixture was stirred at room temperature for 30 min then heated to 70 °C for 2 h. After the reaction was complete, the mixture was cooled to 0 °C and diluted with Et_2O (30 mL). The mixture was then treated sequentially with water (0.6 mL), 15% aq. NaOH solution (0.6 mL), and water (1.8 mL) with intervals of 5 min of stirring between each addition. The mixture was allowed to warm to room temperature before the organics were separated, dried with $MgSO_4$, filtered, and concentrated under reduced pressure. The crude residue was purified by column chromatography (0–40% Et_2O in petroleum ether) to afford the product as a colourless oil (156 mg, 58%).

υ_{max} (film): 3063, 2927, 2859, 2778, 1603, 1479, 1452 cm^{-1}.

^{1}H NMR (400 MHz, $CDCl_3$): δ 7.41 – 7.30 (m, 4H), 7.29 – 7.23 (m, 1H), 7.07 (dd, *J* = 7.3, 1.3 Hz, 1H), 7.05 – 7.01 (m, 1H), 6.66 (td, *J* = 7.4, 1.0 Hz, 1H), 6.37 (d, *J* = 7.7 Hz, 1H), 4.45 (d, *J* = 14.8 Hz, 1H), 4.08 (d, *J* = 14.8 Hz, 1H), 3.39 (dd, *J* = 10.9, 5.7 Hz, 1H), 3.09 (td, *J* = 8.9, 2.8 Hz, 1H), 3.02 (ddt, *J* = 11.0, 4.0, 1.9 Hz, 1H), 2.35 (dt, *J* = 12.8, 8.6 Hz, 1H), 2.29 – 2.19 (m, 2H), 1.95 (ddd, *J* = 12.3, 11.0, 2.9 Hz, 1H), 1.83 – 1.66 (m, 3H), 1.63 – 1.47 (m, 4H), 1.38 – 1.31 (m, 1H), 1.16 – 1.05 (m, 2H), 0.90 – 0.84 (m, 1H), 0.65 (t, *J* = 7.5 Hz, 3H).

^{13}C NMR (101 MHz, $CDCl_3$): δ 150.0, 138.7, 136.9, 128.6, 127.9, 127.3, 127.1, 122.5, 117.4, 106.7, 71.3, 69.2, 53.9, 53.1, 52.6, 48.4, 39.2, 35.6, 34.6, 30.2, 23.1, 22.5, 21.9, 7.0.

HRMS: Exact mass calculated for $[M+H]^+$ ($C_{26}H_{33}N_2$) requires *m/z* 373.2638, found *m/z* 373.2627.

Compound 18

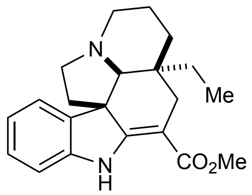

An oven-dried flask with stir bar was sealed and purged with N_2 line fitted then charged with CH_2Cl_2 (2.9 mL). The flask was cooled to −78°C before addition of oxalyl chloride (37 μL, 0.43 mmol, 1.5 equiv) and the mixture was stirred for 30 min. DMSO (83 μL, 1.16 mmol, 4 equiv) dissolved in CH_2Cl_2 (0.3 mL) was then added by syringe and the reaction mixture was stirred for 30 min at −78 °C. A solution of compound **1** (81.9 mg, 0.29 mmol, 1 equiv) in CH_2Cl_2 (2.6 mL) was added dropwise and the mixture was stirred for 1 h at −78 °C. Et_3N (204 μL, 1.45 mmol, 5 equiv) was added dropwise via syringe at −78 °C, before the cooling bath was removed and the reaction mixture was allowed to warm to a room temperature over 1 h. After the reaction was complete, sat. aq. $NaHCO_3$ solution (20 mL) was added and the mixture was extracted with CH_2Cl_2 (3 × 30 mL). The combined organics were then washed once with brine (20 mL) before being dried with Na_2SO_4, filtered, and concentrated under reduced pressure, to give a pale brown oil crude residue, which was carried through to the next step without further purification.

An oven-dried 5 mL MW vial with stir bar was sealed and purged with N_2 line fitted then charged with a solution of the crude residue in THF (2.9 mL) under N_2 atmosphere. The mixture was cooled to −78 °C and stirred for 5 min before dropwise addition of *n*-butyl lithium (2.3 M in hexane, 0.2 mL, 0.46 mmol, 1.6 equiv). The resulting mixture was stirred for 40 min at −78 °C, before dropwise addition of methyl cyanoformate (37 μL, 0.46, 1.6 equiv). The resulting mixture was stirred for 30 min at −78 °C then allowed to warm to room temperature over 1 h. After the reaction was complete, the mixture was concentrated under reduced pressure to give a crude residue, which was purified by column chromatography (0–10% EtOAc in hexane) to afford the product as a colourless oil (14 mg, 14%).

υ_{max} (film): 3366, 2930, 2774, 1672, 1605, 1464, 1435, 1250, 1157, 1111 cm^{-1}.

^{1}H NMR (500 MHz, $CDCl_3$): δ 8.90 (s, 1H), 7.19 (d, *J* = 7.4 Hz, 1H), 7.12 (td, *J* = 7.7, 1.2 Hz, 1H), 6.86 (td, *J* = 7.4, 1.0 Hz, 1H), 6.79 (d, *J* = 7.7, 0.8 Hz, 1H), 3.76 (s, 3H), 3.15 – 3.10 (m, 1H), 2.92 (t, *J* = 7.4 Hz, 1H), 2.72 (d, *J* = 15.1 Hz, 1H), 2.59 – 2.53 (m, 1H), 2.45 (s, 1H), 2.44 – 2.38 (m, 1H), 2.27 (dd, *J* = 15.1, 1.9 Hz, 1H), 2.09 – 2.01 (m, 1H), 1.89 – 1.78 (m, 2H), 1.70 (dd, *J* = 11.7, 4.6 Hz, 1H), 1.57 – 1.51 (m, 1H), 1.29 – 1.21 (m, 1H), 1.01 – 0.94 (m, 1H), 0.67 – 0.54 (m, 4H).

^{13}C NMR (126 MHz, $CDCl_3$): δ 169.3, 167.9, 143.4, 138.1, 127.6, 121.2, 120.7, 109.4, 92.7, 72.8, 55.6, 51.9, 51.2, 50.8, 45.3, 38.2, 33.0, 29.5, 25.7, 22.3, 7.2.

HRMS: Exact mass calculated for $[M+H]^+$ ($C_{21}H_{27}O_2N_2$) requires *m/z* 339.2067, found *m/z* 339.2057. Data in agreement with that reported in the literature.[5]

Compound 19

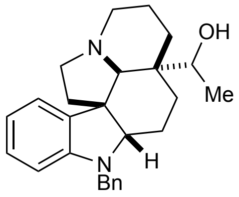

An oven-dried flask with stir bar was charged with compound **16** (676 mg, 1.69 mmol, 1 equiv) then sealed with a septum and purged with N_2 line fitted. THF (17 mL) was added via syringe and the mixture was subsequently cooled to 0 °C. $LiAlH_4$ (1.35 g, 33.8 mmol, 20 equiv) was then added under N_2 flow. The mixture was stirred for 30 min then heated to 70 °C for 2 h. After the reaction was complete, the mixture was cooled to 0 °C and diluted with Et_2O (20 mL). Water (1.35 mL), 15% aq. NaOH solution (1.35 mL), and water (4.05 mL) were then added sequentially with intervals of 5 minutes stirring between each addition. The mixture was allowed to warm room temperature and the organics were separated, dried with $MgSO_4$, filtered, and concentrated under reduced pressure to yield the product as a beige foam solid (578 mg, 88%) as an inconsequential inseparable mixture of diastereomers (3:1 ratio). No further purification was required.

υ_{max} (film): 3379, 2934, 2864, 1603, 1479, 1452, 1265, 1070 cm^{-1}.

^{1}H NMR (700 MHz, $CDCl_3$): δ 7.38 – 7.35 (m, 2H), 7.32 (dd, *J* = 8.3, 6.9 Hz, 2H), 7.27 – 7.24 (m, 1H), 7.02 (td, *J* = 7.6, 1.3 Hz, 1H), 6.99 (dd, *J* = 7.3, 1.3 Hz, 1H), 6.64 (td, *J* = 7.4, 0.9 Hz, 1H), 6.37 (d, *J* = 7.8 Hz, 1H), 4.44 (d, *J* = 14.9 Hz, 1H), 4.08 (dd, *J* = 14.8, 8.7 Hz, 1H), 3.88 (p, *J* = 5.8 Hz, 1H), 3.41 (dt, *J* = 10.6, 6.6 Hz, 1H), 3.09 (td, *J* = 9.0, 3.1 Hz, 1H), 3.00 (ddd, *J* = 11.3, 4.1, 2.0 Hz, 1H), 2.34 – 2.30 (m, 1H), 2.29 (s, 1H), 2.27 – 2.22 (m, 1H), 1.99 – 1.91 (m, 1H), 1.88 (td, *J* = 14.5, 3.7 Hz, 1H), 1.79 (ddt, *J* = 13.6, 10.2, 4.8 Hz, 1H), 1.72 – 1.68 (m, 1H), 1.62 – 1.53 (m, 2H), 1.53 – 1.43 (m, 3H), 1.38 (td, *J* = 13.4, 4.7 Hz, 1H), 1.15 (d, *J* = 6.2 Hz, 3H). Alcohol proton not observed.

^{13}C NMR (126 MHz, $CDCl_3$): δ 150.3, 150.1, 138.6, 138.6, 136.6, 135.6, 128.7, 128.6, 127.9, 127.9, 127.7, 127.7, 127.2, 127.2, 122.3, 121.5, 117.8, 117.5, 106.9, 106.9, 68.7, 67.1, 66.9, 66.1, 63.0, 53.8, 53.6, 53.2, 53.0, 52.8, 52.3, 48.6, 48.4, 39.9, 39.6, 39.2, 39.1, 30.0, 26.6, 26.0, 23.8, 22.3, 22.1, 21.4, 21.3, 18.0, 16.6.

HRMS (ESI): Exact mass calculated for $[M+H]^+$ ($C_{26}H_{33}N_2O$) requires *m/z* 389.2587, found *m/z* 389.2578.

Compound 20

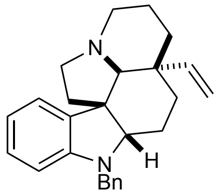

An oven-dried 25 mL MW vial with stir bar was charged with compound **19** (180 mg, 0.46 mmol, 1 equiv) and Martin Sulfurane (649 mg, 0.93 mmol, 2 equiv). The vial was sealed and purged with N_2 line fitted before addition of $CHCl_3$ (9.3 mL) via syringe. The reaction mixture was then stirred at room temperature for 3 h. After the reaction was complete, the reaction mixture was concentrated under reduced pressure to give a crude residue, which was purified by column chromatography (silica gel, 0–10% EtOAc in hexane with 1% Et_3N additive) to afford the desired product as a pale yellow solid (138 mg, 80%).

υ_{max} (film): 2930, 2783, 2357, 1481, 1454, 905 cm^{-1}.

^{1}H NMR (500 MHz, $CDCl_3$): δ 7.39 – 7.36 (m, 2H), 7.34 – 7.30 (m, 2H), 7.28 – 7.24 (m, 1H), 7.05 (dd, *J* = 7.3, 1.3 Hz, 1H), 7.02 (td, *J* = 7.6, 1.3 Hz, 1H), 6.66 (td, *J* = 7.4, 1.0 Hz, 1H), 6.35 (d, *J* = 7.8 Hz, 1H), 5.95 – 5.79 (m, 1H), 4.88 – 4.82 (m, 1H), 4.77 (d, *J* = 11.0 Hz, 1H), 4.42 (d, *J* = 14.7 Hz, 1H), 4.06 (d, *J* = 14.7 Hz, 1H), 3.39 (dd, *J* = 11.3, 5.6 Hz, 1H), 3.16 – 3.07 (m, 1H), 3.03 (d, *J* = 11.0 Hz, 1H), 2.37 (d, *J* = 10.4 Hz, 2H),

2.27 (q, J = 8.3, 7.5 Hz, 1H), 2.04 – 1.94 (m, 2H), 1.77 – 1.68 (m, 2H), 1.58 – 1.39 (m, 5H), 1.28 – 1.22 (m, 1H).

^{13}C NMR (126 MHz, $CDCl_3$) δ 150.3, 145.7, 138.7, 135.5, 128.6, 127.9, 127.4, 127.1, 122.9, 117.4, 111.5, 106.8, 70.5, 68.7, 53.7, 52.9, 52.7, 48.5, 39.1, 38.2, 36.6, 25.3, 22.8, 21.9.

HRMS (ESI): Exact mass calculated for $[M+H]^+$ ($C_{26}H_{31}N_2$) requires *m/z* 371.2482, found *m/z* 371.2470.

Data in agreement with that reported in the literature.[5]

Compound 21

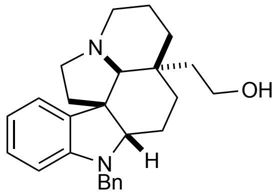

An oven-dried 25 mL MW vial with stir bar was sealed and purged with N_2 line fitted then charged with compound **20** (151 mg, 0.41 mmol, 1 equiv) as a solution in THF (3.4 mL) via syringe. The mixture was then cooled to 0 °C and stirred for 5 min before addition of 9-BBN in THF (0.5 M, 1.63 mL, 0.82 mmol, 2 equiv) dropwise via syringe. The reaction mixture was stirred and allowed to warm to room temperature then heated to 60 °C for 18 h. The mixture was cooled to 0 °C and stirred for 15 min. H_2O_2 (aq. 30% w/v, 0.83 mL, 8.15 mmol, 20 equiv) and 2 M NaOH solution (0.82 mL, 1.63 mmol, 4 equiv) were added sequentially via syringe. The reaction was stirred, allowed to reach room temperature, and stirred for 1 h. After the reaction was complete, the mixture was cooled to 0 ºC and stirred before carefully quenching with sat. aq. $Na_2S_2O_3$ solution and stirring until effervescence ceased. The via was vented and uncapped and the mixture was treated with sat. aq. NH_4Cl solution (20 mL). The mixture was extracted with EtOAc (3 × 20 mL). The combined organics were washed with brine (15 mL), dried over Na_2SO_4, filtered, and concentrated under reduced pressure. The crude residue was purified by column chromatography (silica gel, 0–30% EtOAc in hexane + 1% Et_3N additive) to afford the desired product as a beige solid (115 mg, 73%).

υ_{max} (film): 3337, 2928, 2781, 2359, 1603, 1481, 1452, 1265 cm^{-1}.

^{1}H NMR (700 MHz, $CDCl_3$): δ 7.37 (d, J = 7.2 Hz, 2H), 7.32 (dd, J = 8.4, 6.8 Hz, 2H), 7.26 (dt, J = 14.5, 1.5 Hz, 1H), 7.06 (dd, J = 7.3, 1.3 Hz, 1H), 7.01 (td, J = 7.6, 1.3 Hz, 1H), 6.66 (td, J = 7.4, 1.0 Hz, 1H), 6.36 (d, J = 7.8 Hz, 1H), 4.44 (d, J = 14.7 Hz, 1H), 4.08 (d, J = 14.7 Hz, 1H), 3.59 (td, J = 10.1, 5.5 Hz, 1H), 3.51 (td, J = 10.0, 6.2 Hz, 1H), 3.41 – 3.36 (m, 1H), 3.07 (t, J = 9.5 Hz, 1H), 3.00 (d, J = 10.8 Hz, 1H), 2.34 (q, J = 10.0 Hz, 1H), 2.29 – 2.19 (m, 2H), 1.96 (t, J = 11.6 Hz, 1H), 1.88 (t, J = 13.7 Hz, 1H), 1.82 – 1.74 (m, 2H), 1.74 – 1.66 (m, 1H), 1.62 (d, J = 13.7 Hz, 1H), 1.59 – 1.52 (m, 1H), 1.49 (d, J = 12.5 Hz, 1H), 1.38 (tdd, J = 14.0, 11.0, 3.6 Hz, 1H), 1.29 – 1.23 (m, 1H), 1.22 – 1.16 (m, 1H), 1.04 (d, J = 13.7 Hz, 1H). Alcohol proton not observed.

^{13}C NMR (176 MHz, $CDCl_3$): δ 150.0, 138.6, 136.4, 128.6, 127.9, 127.5, 127.2, 122.4, 117.6, 106.9, 70.8, 68.8, 58.7, 53.8, 52.9, 52.6, 48.5, 40.7, 38.9, 35.5, 35.4, 24.3, 22.6, 21.8.

HRMS (ESI): Exact mass calculated for $[M+H]^+$ ($C_{26}H_{33}ON_2$) requires *m/z* 389.2587, found *m/z* 389.2579.

Data in agreement with that reported in the literature.[6]

Compound 22

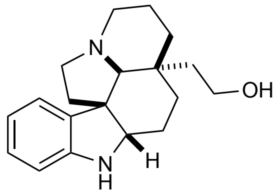

A round bottom flask was charged with stir bar, compound **21** (115 mg, 0.3 mmol, 1 equiv), and Pearlman's catalyst (20% $Pd(OH)_2/C$; 384 mg, 0.55 mmol, 1.85 equiv). The flask was then sealed with a septum and

purged with N_2. EtOH (9.9 mL) was added and the reaction mixture was sparged with H_2. The resulting mixture was stirred at room temperature for 26 h under H_2 atmosphere. After the reaction was complete, the vial was purged with N_2, decapped, and the mixture was filtered through a pad of Celite®. The filtrate was concentrated to a crude residue, which was purified by column chromatography (silica gel, 0–100% EtOAc in hexane + 1% Et_3N additive) to afford the product as a white solid (70.2 mg, 79%).

υ_{max} (film): 3310 (br), 2928, 1462, 1258, 1043, 741 cm^{-1}.

^{1}H NMR (700 MHz, $CDCl_3$): δ 7.08 (d, J = 7.4 Hz, 1H), 7.01 (t, J = 7.6 Hz, 1H), 6.73 (t, J = 7.4 Hz, 1H), 6.63 (d, J = 7.7 Hz, 1H), 3.62 (td, J = 10.1, 5.4 Hz, 1H), 3.52 (dp, J = 16.5, 6.1 Hz, 2H), 3.11 (dt, J = 9.0, 5.5 Hz, 1H), 3.04 (d, J = 11.0 Hz, 1H), 2.32 – 2.19 (m, 3H), 2.04 (td, J = 13.9, 3.4 Hz, 1H), 2.00 – 1.94 (m, 1H), 1.75 (ddq, J = 18.2, 8.5, 5.3, 4.4 Hz, 2H), 1.72 – 1.67 (m, 1H), 1.64 (d, J = 13.7 Hz, 1H), 1.48 (dddd, J = 24.7, 17.5, 10.6, 3.8 Hz, 3H), 1.26 (td, J = 13.5, 4.6 Hz, 1H), 1.18 (ddd, J = 14.6, 9.5, 5.7 Hz, 1H), 1.02 (dd, J = 13.2, 4.0 Hz, 1H). Alcohol and indoline protons not observed.

^{13}C NMR (176 MHz, $CDCl_3$): δ 149.6, 135.4, 127.5, 122.9, 119.3, 110.6, 70.9, 65.5, 58.7, 53.9, 53.6, 53.0, 40.6, 38.7, 35.6, 35.5, 28.3, 24.4, 21.8.

HRMS (ESI): Exact mass calculated for $[M+H]^+$ ($C_{19}H_{27}N_2O$) requires *m/z* 299.2118, found *m/z* 299.2111.

Data in agreement with that reported in the literature.[7]

Compound 23

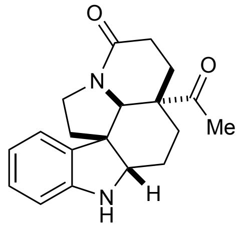

An oven-dried MW vial was charged with stir bar, Pearlman's catalyst (20% $Pd(OH)_2/C$; 324 mg, 0.46 mmol, 1.85 equiv), and compound **16** (100 mg, 0.097 mmol, 1 equiv). The vial was then capped and purged. EtOH (8.3 mL) was added and the vial was sparged with H_2. The resulting mixture was stirred at room temperature for 26 h under H_2 atmosphere. After the reaction was complete, the vial was purged with N_2, decapped, and the mixture was filtered through a pad of Celite®. The filtrate was concentrated to a crude residue, which was purified by column chromatography (0–10% MeOH in CH_2Cl_2) to afford the product as a pale yellow solid (45.9 mg, 59%).

υ_{max} (film): 3302, 2936, 2887, 1701, 1620, 1464, 1416 cm^{-1}.

^{1}H NMR (500 MHz, $CDCl_3$): δ 7.16 (dd, J = 7.6, 1.3 Hz, 1H), 7.03 (td, J = 7.6, 1.3 Hz, 1H), 6.77 (td, J = 7.5, 1.0 Hz, 1H), 6.59 (d, J = 7.8 Hz, 1H), 4.73 (s, 1H), 3.68 (dd, J = 12.6, 9.7 Hz, 1H), 3.57 (td, J = 11.9, 7.6 Hz, 1H), 3.28 (dd, J = 10.4, 5.5 Hz, 1H), 2.53 – 2.47 (m, 1H), 2.39 (ddd, J = 18.1, 11.1, 7.0 Hz, 1H), 2.29 (ddd, J = 13.2, 7.7, 1.3 Hz, 1H), 2.06 (s, 3H), 1.97 – 1.84 (m, 2H), 1.76 (dddt, J = 13.8, 10.2, 7.0, 3.0 Hz, 2H), 1.67 (ddd, J = 13.1, 11.3, 9.7 Hz, 1H), 1.56 (td, J = 13.8, 2.9 Hz, 1H), 1.35 (tdd, J = 13.4, 10.4, 3.0 Hz, 1H). Indoline proton not observed.

^{13}C NMR (126 MHz, $CDCl_3$): δ 208.7, 168.3, 149.4, 128.5, 128.4, 124.6, 119.3, 110.4, 60.3, 60.0, 55.1, 50.1, 43.3, 35.3, 31.0, 28.0, 27.9, 25.2, 22.4.

HRMS: Exact mass calculated for $[M+Na]^+$ ($C_{19}H_{22}N_2O_2Na$) requires *m/z* 333.1573, found *m/z* 333.1564.

Compound 24

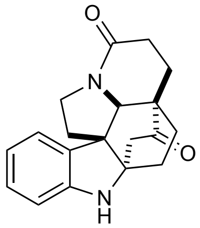

An oven-dried, purged, and capped MW vial equipped with stir bar with N_2 line fitted was charged with CH_2Cl_2 (11.5 mL). The vessel was cooled to –60 °C, before oxalyl chloride (39 µL, 0.46 mmol, 1.2 equiv) was added. A solution of DMSO (58 µL, 0.81 mmol, 2.1 equiv) in CH_2Cl_2 (3 mL) was added, and the mixture was stirred for 15 min. A solution of compound **23** (120 mg, 0.39 mmol, 1 equiv) in CH_2Cl_2 (3 mL, 0.018 M) was then added *via* syringe and the mixture was stirred for 30 min at –60 °C. Et_3N (269 µL, 0.34 mmol, 5 equiv) was then added dropwise and the reaction mixture was allowed to warm to room temperature over 45 min. After the reaction was complete, water (20 mL) was added, and the organics were extracted with CH_2Cl_2 (3 × 25 mL). The combined organics were then washed with brine (20 mL), dried with Na_2SO_4, filtered, and concentrated under reduced pressure, to give a pale yellow oil, which was carried through to the next step without further purification.

In a round bottom flask, the crude residue was dissolved in ethanol (60 mL, 0.007 M), and conc. HCl (5 N, 6 mL) was added. The reaction mixture was then refluxed for 18 h. The mixture was concentrated under reduced pressure to remove the volatile organics. The mixture was then treated with 5% aq. Na_2CO_3 solution (100 mL) and extracted with CH_2Cl_2 (3 × 50 mL). The combined organics were dried with Na_2SO_4, filtered, and concentrated under reduced pressure. The crude residue was purified by column chromatography (0–10% MeOH in EtOAc) to afford the product as a beige solid (51.1 mg, 43%).

υ_{max} (film): 3294, 2934, 2897, 1726, 1612, 1601, 1460, 1449, 1410 cm^{-1}.

^{1}H NMR (500 MHz, $CDCl_3$): δ 7.13 (t, *J* = 7.7 Hz, 1H), 7.09 (d, *J* = 7.3 Hz, 1H), 6.82 (t, *J* = 7.4 Hz, 1H), 6.75 (d, *J* = 7.8 Hz, 1H), 4.34 (dd, *J* = 11.8, 7.8 Hz, 1H), 3.80 (s, 1H), 3.77 (s, 1H), 3.29 (td, *J* = 12.0, 5.7 Hz, 1H), 2.71 (dd, *J* = 18.4, 3.6 Hz, 1H), 2.51 (ddd, *J* = 14.4, 8.2, 6.1 Hz, 1H), 2.42 – 2.36 (m, 2H), 2.32 (td, *J* = 12.4, 8.0 Hz, 2H), 2.11 – 1.99 (m, 2H), 1.85 (ddd, *J* = 12.8, 10.9, 7.4 Hz, 1H), 1.60 (dd, *J* = 12.7, 5.7 Hz, 1H), 1.43 (dt, *J* = 14.9, 7.6 Hz, 1H), 1.37 – 1.29 (m, 1H).

^{13}C NMR (126 MHz, $CDCl_3$): δ 209.6, 169.4, 148.9, 135.3, 128.7, 121.7, 120.5, 111.8, 67.0, 61.1, 57.7, 48.0, 47.9, 43.8, 33.8, 29.6, 26.8, 23.7, 22.0.

HRMS: Exact mass calculated for $[M+Na]^+$ ($C_{19}H_{20}N_2O_2Na$) requires *m/z* 331.1417, found *m/z* 331.1417.

Compound 25

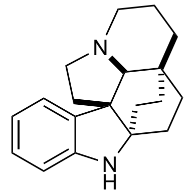

An oven-dried round bottomed flask with stir bar and condenser was purged and sealed with N_2 line fitted before charging with a solution of compound **24** (50 mg, 0.162 mmol, 1 equiv), Na (650 mg, 28.2 mmol, 174 equiv), and hydrazine monohydrate (1.95 mL, 40.2 mmol, 248 equiv) in ethylene glycol (16.2 mL). The mixture was heated at 160 °C for 1 h, 190 °C for 2.5 h, and 210 °C for 18 h. The resulting mixture was allowed to cool to room temperature before quenching with water (25 mL) and extracted with EtOAc (3 × 30 mL). The combined organic extracts were washed with brine (5 × 50 mL), dried with Na_2SO_4, filtered, and concentrated under reduced pressure, to give a pale yellow oil crude residue, which was carried through to the next step without further purification.

An oven-dried flask with stir bar was sealed and purged with N_2 line fitted and charged with a solution of the crude residue in THF (6.5 mL) was added and subsequently cooled to 0 °C. $LiAlH_4$ (129 mg, 3.24 mmol, 20 equiv) was then added under N_2 flow. The mixture was stirred at room temperature for 30 min then heated to 70 °C for 2 h. After the reaction was complete, the mixture was cooled to 0 °C and diluted with Et_2O (20 mL).

The mixture was then treated sequentially with water (0.2 mL), 15% aq. NaOH solution (0.2 mL), and water (0.6 mL) with intervals of 5 min of stirring between each addition. The mixture was allowed to warm to room temperature before the organics were separated, dried with $MgSO_4$, filtered, and concentrated under reduced pressure. The organics were separated and dried with $MgSO_4$, filtered, and concentrated under reduced pressure. The crude residue was purified by column chromatography (0–10% MeOH in CH_2Cl_2) to afford the product as a pale yellow oil. The product was further purified by acid-base extraction: the material was treated with 2 M HCl (20 mL) and extracted with CH_2Cl_2 (2 × 20 mL; discarded). The aqueous phase was then basified with 2 M aq. NaOH solution (25 mL) and extracted with CH_2Cl_2 (2 × 20 mL). The combined organics were dried with Na_2SO_4, filtered, and concentrated under reduced pressure to give the product as a yellow oil (32.9 mg, 72%).

υ_{max} (film): 3343, 2926, 2855, 1609, 1460 cm^{-1}.

^{1}H NMR (700 MHz, $CDCl_3$): δ 7.31 (d, *J* = 7.2 Hz, 1H), 7.00 (td, *J* = 7.6, 1.3 Hz, 1H), 6.78 (td, *J* = 7.4, 1.0 Hz, 1H), 6.64 (dd, *J* = 7.8, 0.8 Hz, 1H), 3.23 (q, *J* = 8.4 Hz, 1H), 3.11 (ddt, *J* = 13.5, 4.0, 1.5 Hz, 1H), 3.08 (s, 1H), 3.04 (td, *J* = 9.0, 3.7 Hz, 1H), 3.02 – 2.97 (m, 1H), 2.70 (ddd, *J* = 13.8, 8.4, 3.5 Hz, 1H), 2.24 – 2.14 (m, 2H), 1.85 – 1.77 (m, 2H), 1.73 (td, *J* = 12.2, 11.7, 5.7 Hz, 1H), 1.65 (dt, *J* = 13.9, 8.3 Hz, 1H), 1.52 – 1.49 (m, 1H), 1.40 (ddd, *J* = 12.6, 11.3, 6.8 Hz, 1H), 1.30 – 1.24 (m, 3H), 1.23 – 1.17 (m, 2H). Indoline proton not observed.

^{13}C NMR (101 MHz, $CDCl_3$): δ 150.1, 140.2, 126.8, 122.3, 119.9, 110.9, 69.1, 64.6, 57.1, 50.8, 47.9, 36.1, 35.2, 34.8, 31.5, 31.5, 29.3, 26.6, 17.2.

HRMS: Exact mass calculated for $[M+H]^+$ ($C_{19}H_{25}N_2$) requires *m/z* 281.2012, found *m/z* 281.2010.

Data in agreement with that reported in the literature.[8]

Compound S1

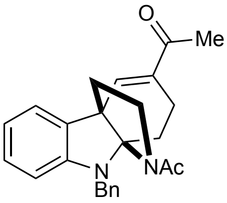

An oven-dried 5 mL MW vial with stir bar was charged *N*-(2-(1-benzyl-2-vinyl-1*H*-indol-3-yl)ethyl)acetamide (79.5 mg, 0.25 mmol, 1 equiv). The vial was capped and purged before sequential addition of addition of 3-butyn-2-one (98 µL, 1.25 mmol, 5 equiv), 1,4-dioxane (1 mL) and BF_3•OEt_2 (49 µL, 0.5 mmol, 1.6 equiv) via syringe. The reaction was then stirred at 25 °C for 4 h. After the reaction was complete the reaction mixture was vented and de-capped. The reaction mixture was treated with MeOH (5 mL) and concenyrated under reduced pressure. The residue was treated with sat. aq. $NaHCO_3$ solution (20 mL) and extracted with EtOAc (3 × 20 mL). The combined organics were dried with Na_2SO_4, filtered, and concentrated under reduced pressure. The crude residue was purified by column chromatography (silica gel, 0–20% EtOAc in CH_2Cl_2) to afford the product as a pale yellow solid (149 mg, 77%). Crystal growth by solvent diffusion using CH_2Cl_2/pentane.

υ_{max} (film): 3005, 2970, 2936, 2359, 1645, 1603, 1485, 1398, 1383, 1233, 1217 cm^{-1}.

^{1}H NMR (500 MHz, $CDCl_3$): δ 7.23 (tt, *J* = 7.1, 1.0 Hz, 2H), 7.16 (qd, *J* = 6.6, 5.9, 3.2 Hz, 4H), 6.99 (td, *J* = 7.7, 1.3 Hz, 1H), 6.83 (d, *J* = 1.5 Hz, 1H), 6.71 (td, *J* = 7.4, 1.0 Hz, 1H), 6.13 (d, *J* = 7.8 Hz, 1H), 5.09 (d, *J* = 17.4 Hz, 1H), 4.75 (d, *J* = 17.4 Hz, 1H), 3.64 – 3.54 (m, 1H), 3.42 (td, *J* = 9.7, 6.4 Hz, 1H), 3.18 – 3.09 (m, 1H), 2.50 (ddd, *J* = 12.4, 6.4, 3.3 Hz, 1H), 2.35 – 2.25 (m, 3H), 2.24 – 2.15 (m, 3H), 1.99 (s, 3H).

^{13}C NMR (126 MHz, $CDCl_3$): δ 198.7, 170.6, 149.0, 139.1, 139.1, 138.1, 129.5, 129.1, 128.4, 126.4, 126.3, 122.2, 117.9, 108.4, 90.9, 57.1, 48.2, 48.0, 34.8, 25.6, 25.6, 24.7, 20.7.

HRMS: Exact mass calculated for $[M+Na]^+$ ($C_{25}H_{26}O_2N_2Na$) requires *m/z* 409.1886, found *m/z* 409.1878.

3. X-Ray Crystallography Data

CCDC 2191599 (compound **13**), 2191600 (compound **16**), and 2191601 (compound **S1**) contain the supplementary crystallographic datafor this study. The data can be obtained free of charge from the Cambridge Crystallographic Data Centre via www.ccdc.cam.ac.uk/structures.

Compound 13: X-ray quality crystals isolated by recrystallisation from MeCN.

Compound 16: X-ray quality crystals isolated by recrystallisation from MeCN.

Compound S1: X-ray quality crystals isolated by solvent diffusion from CH_2Cl_2/pentane.

X-ray diffraction data for compounds **16** and **S1** were collected at 93 K (**16**) or 173 K (**S1**) using a Rigaku FR-X Ultrahigh Brilliance Microfocus RA generator/confocal optics with XtaLAB P200 diffractometer [Mo Kα radiation (λ = 0.71075 Å)]. Diffraction data for compound **13** were collected at 173 K using a Rigaku MM-007HF High Brilliance RA generator/confocal optics with XtaLAB P100 diffractometer [Cu Kα radiation (λ = 1.54187 Å)]. Data for all compounds were collected using CrystalClear[9] and processed (including correction for Lorentz, polarization and absorption) using either CrystalClear[9] or CrysAlisPro.[10] Structures were solved by charge-flipping (Superflip[11]) or dual-space (SHELXT[12]) methods, and refined by full-matrix least-squares against F^2 (SHELXL-2018/3[13]). Non-hydrogen atoms were refined anisotropically, and hydrogen atoms were refined using a riding model. All calculations were performed using the CrystalStructure[14] or Olex2[15] interface.

Compound 13

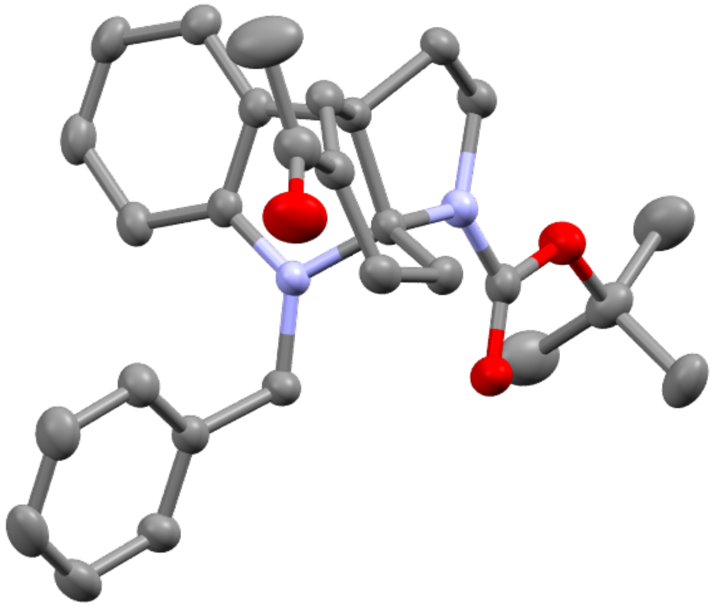

Compound 16

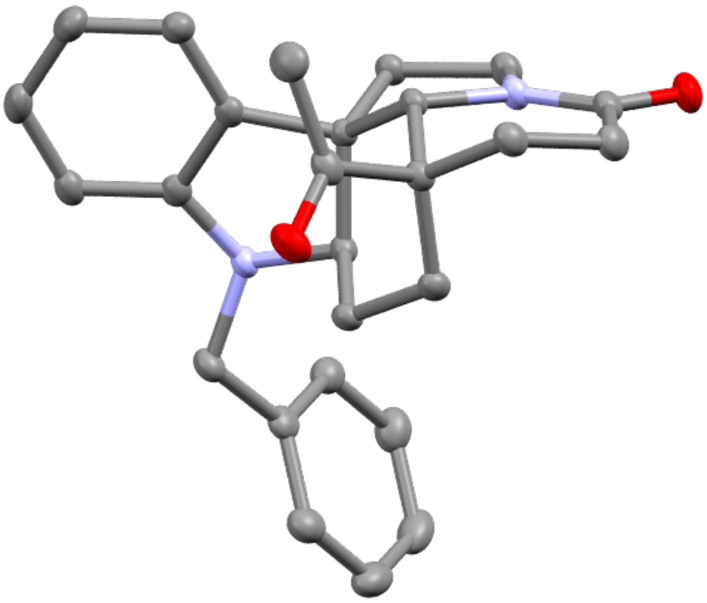

Compound S1

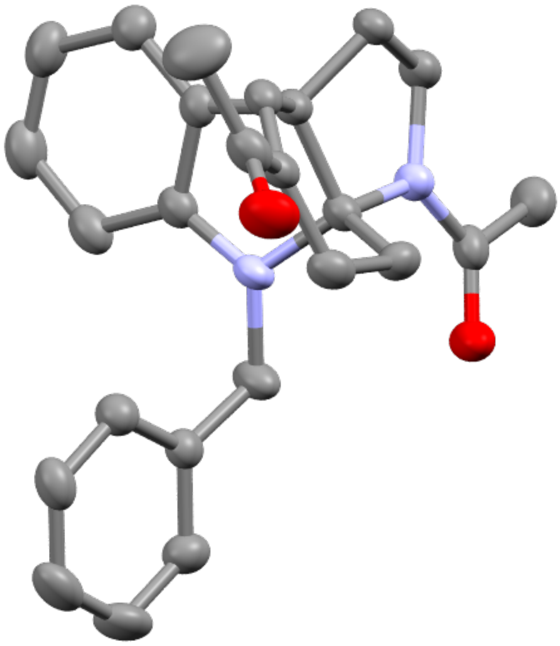

4. References

1. Armarego, W. L. F. Purification of Laboratory Chemicals, 8th Ed., Elsevier: Amsterdam, 2017.

2. M. Kawano, T. Kiuchi, S. Negishi, H. Tanaka, T. Hoshikawa, J. Matsuo and H. Ishibashi, *Angew. Chem. Int. Ed.*, 2013, **52**, 906–910.

3. S. Roy, S. Haque and G. W. Gribble, *Synthesis,* 2006, **23**, 3948–3954.

4. S. C. Benson, L. Lee, L. Yang and J. K. Snyder, *Tetrahedron*, 2000, **56**, 1165–1180.

5. S. B. Jones, B. Simmons, A. Mastracchio and D. W. C. MacMillan, *Nature*, 2011, **475**, 183–188

6. J.-Y. Du, C. Zeng, X.-J. Han, H. Qu, X.-H. Zhao, X.-T. An and C.-A. Fan, *J. Am. Chem. Soc.*, 2015, **137**, 4267–4273.

7. G. Martin, P. Angyal, O. Egyed, S. Varga and T. Soós, *Org. Lett.*, 2020, **20**, 4675–4679.

8. S. Varga, P. Angyal, G. Martin, O. Egyed, T. Holczbauer and T. Soós, *Angew. Chem. Int. Ed.*, 2020, **50**, 13547–13551.

9. *CrystalClear-SM Exper*t v2.1. Rigaku Americas, *The Woodlands, Texas, USA*, and Rigaku Corporation, *Tokyo, Japan*, 2015.

10. *CrysAlisPro* v1.171.38.46. Rigaku Oxford Diffraction, Rigaku Corporation, *Oxford, U.K.*, 2015.

11. Palatinus, L. and Chapuis, G. *J. Appl. Cryst*. 2007, **40**, 786–790.

12. Sheldrick, G. M. *Acta Crystallogr. Sect. A.*, 2015, **71**, 3–8.

13. Sheldrick, G. M. *Acta Crystallogr. Sect. C.*, 2015, **71**, 3–8.

14. *CrystalStructure* v4.3.0. Rigaku Americas, *The Woodlands, Texas, USA*, and Rigaku Corporation, *Tokyo, Japan*, 2018.

15. Dolomanov, O. V.; Bourhis, L. J.; Gildea, R. J.; Howard, J. A. K.; Puschmann, H. *J. Appl. Crystallogr.*, 2009, **42**, 339–341.

5. ¹H and ¹³C NMR Spectra

Compound 1

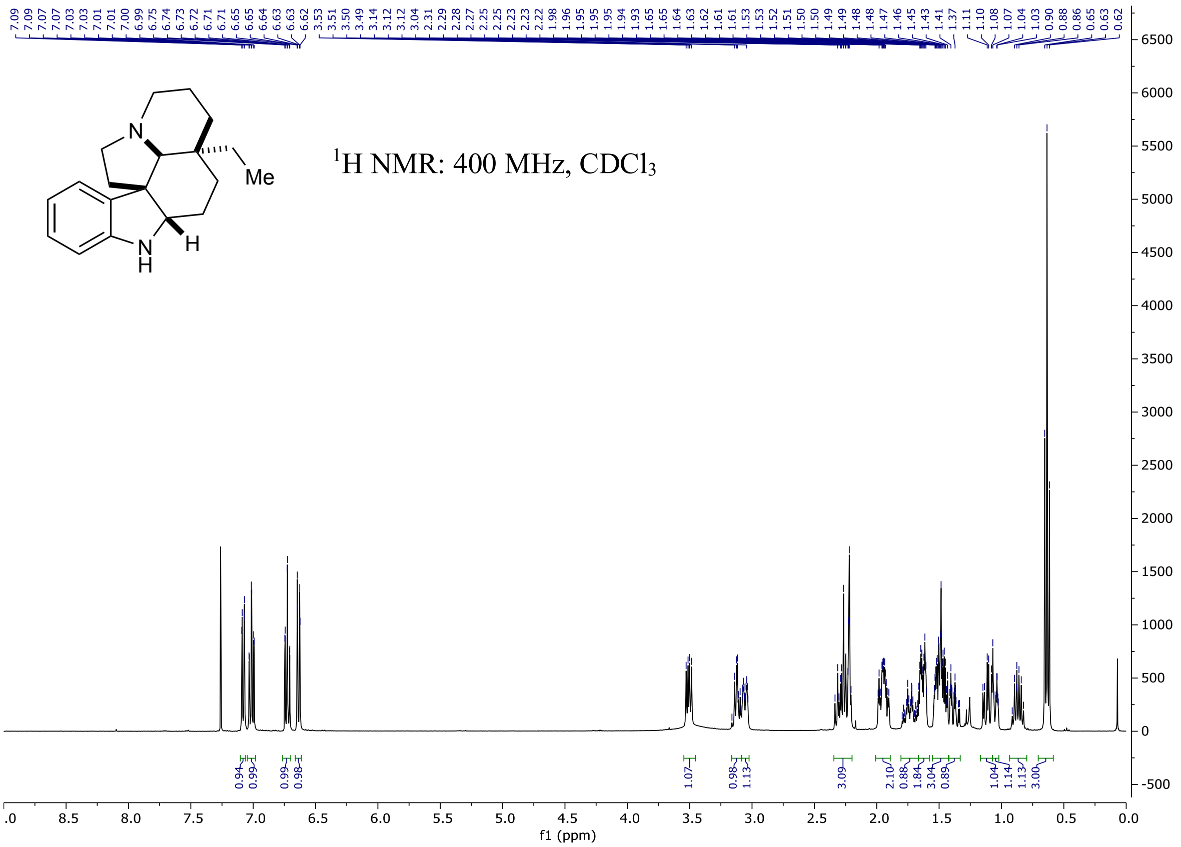

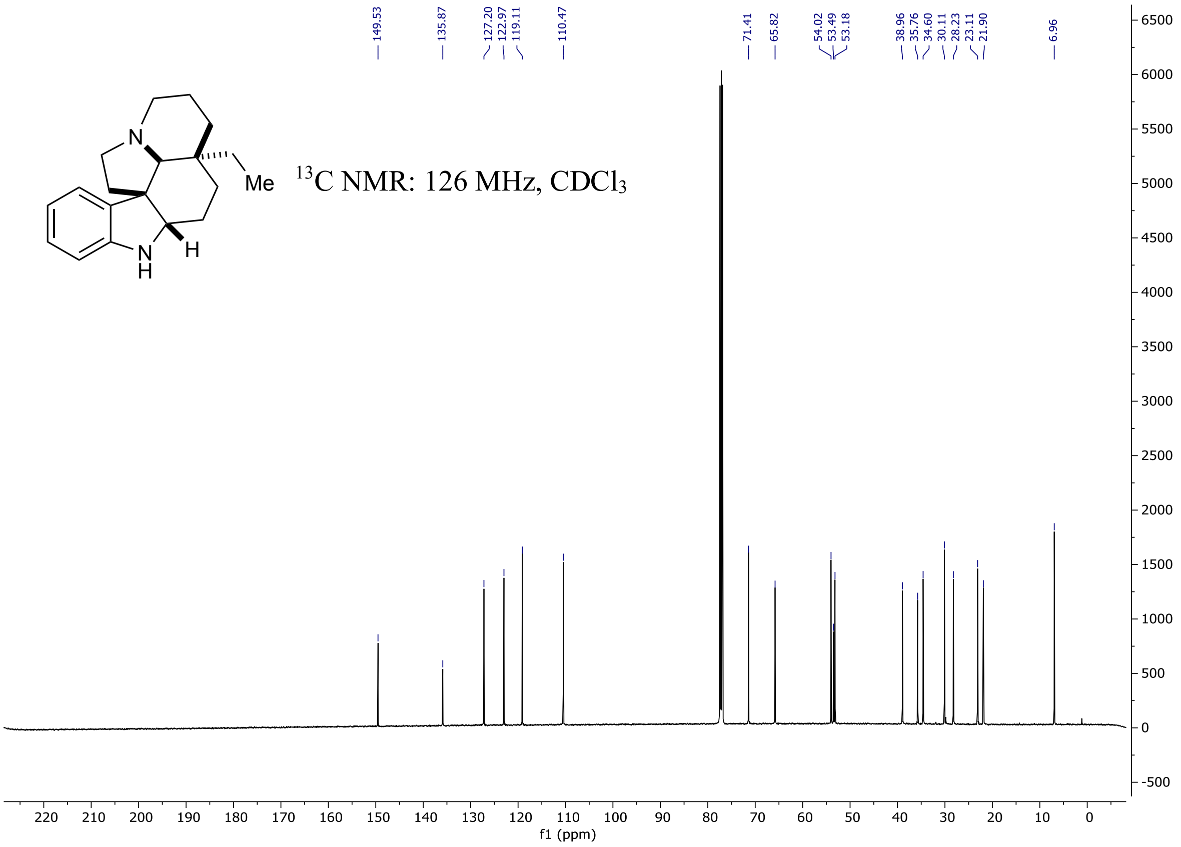

Compound 7

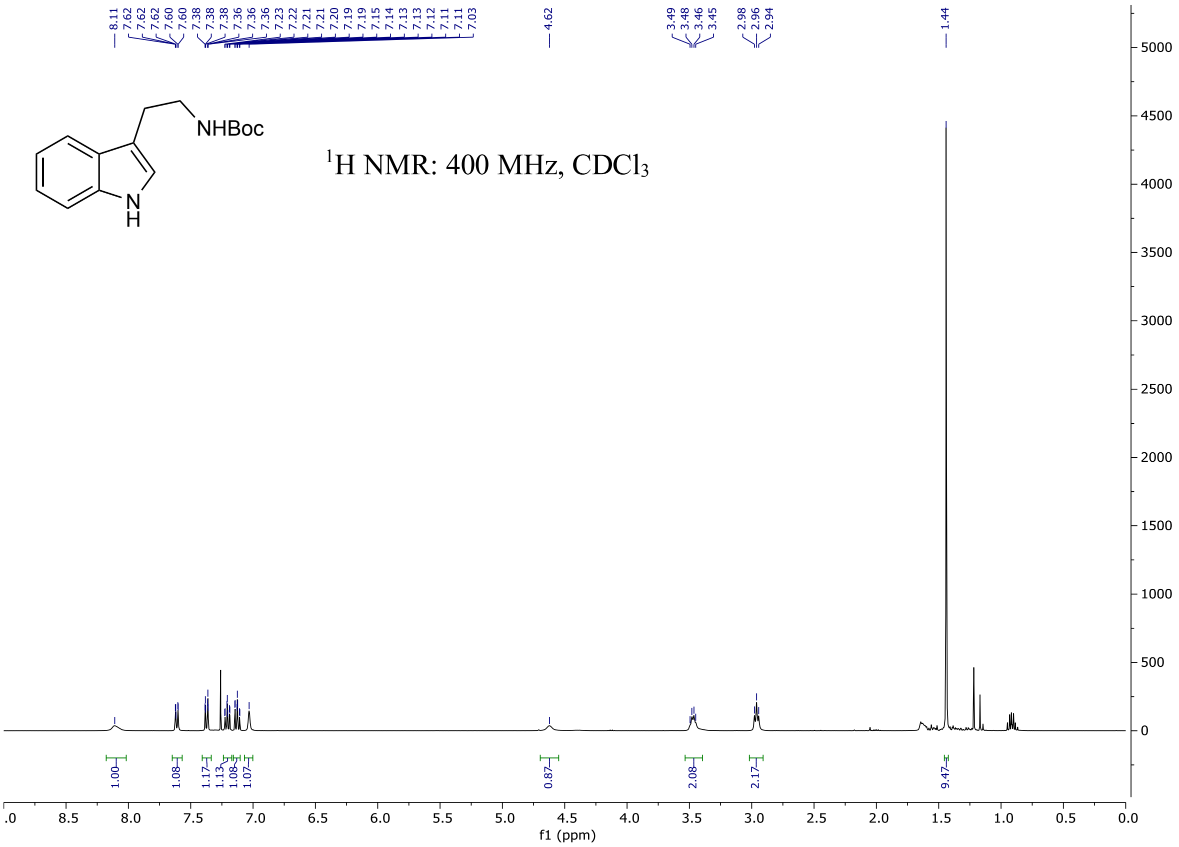
NHBoc
N
H
^{1}H NMR: 400 MHz, CDCl$_3$
8.11 7.62 7.62 7.62 7.60 7.38 7.38 7.38 7.36 7.36 7.36 7.23 7.22 7.21 7.21 7.20 7.19 7.19 7.15 7.14 7.13 7.13 7.12 7.11 7.11 7.03
4.62
3.49 3.48 3.46 3.45
2.98 2.96 2.94
1.44
1.00 1.08 1.17 1.13 1.08 1.07 0.87 2.08 2.17 9.47
f1 (ppm)

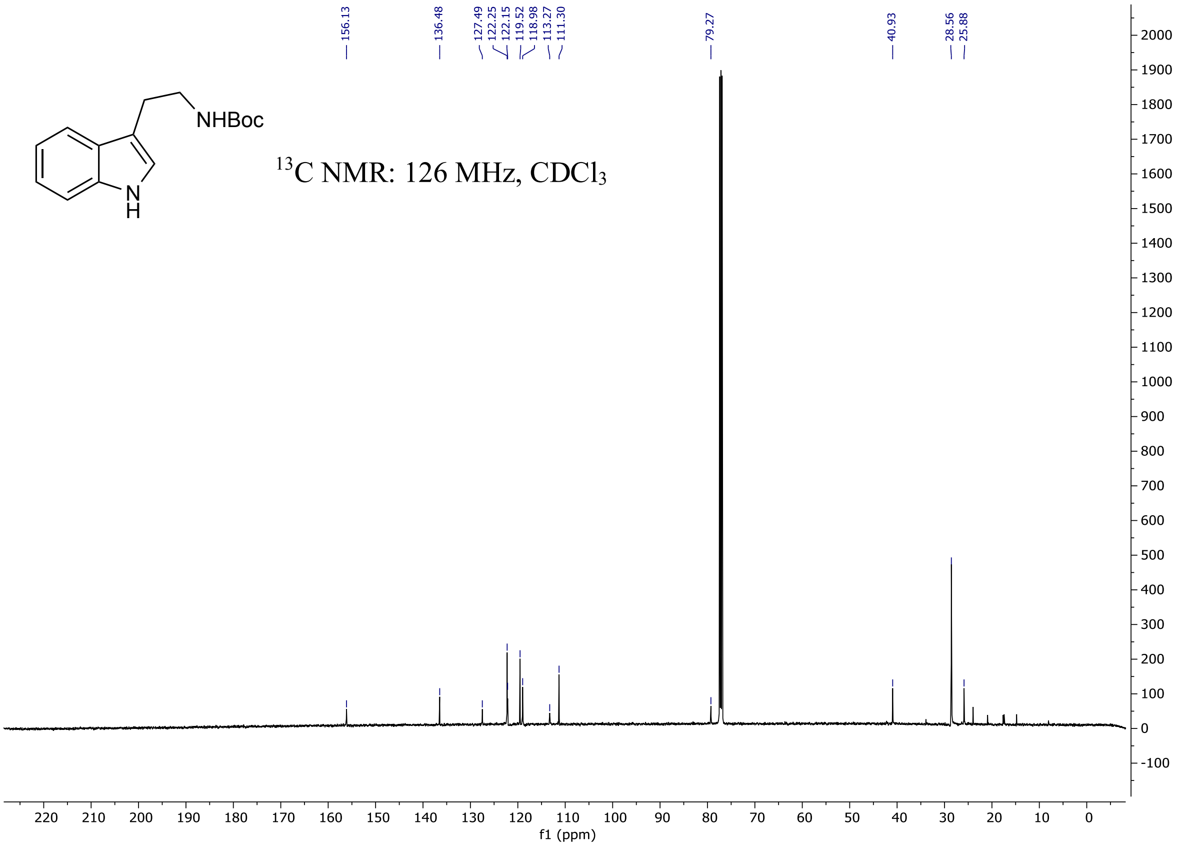
NHBoc
N
H
^{13}C NMR: 126 MHz, CDCl$_3$
156.13
136.48
127.49 122.25 122.15 119.52 118.98 113.27 111.30
79.27
40.93
28.56 25.88
f1 (ppm)

Compound 8

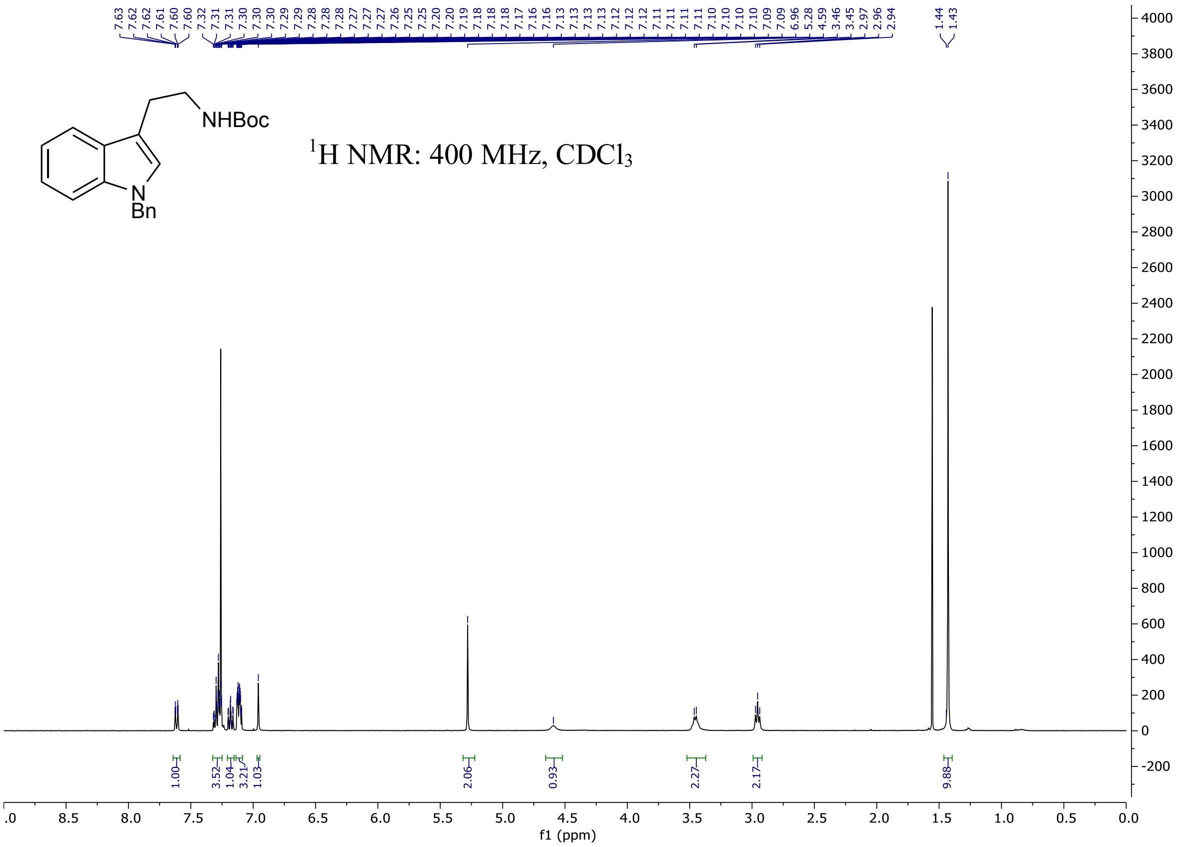
NHBoc
N
Bn
^{1}H NMR: 400 MHz, CDCl$_3$
f1 (ppm)

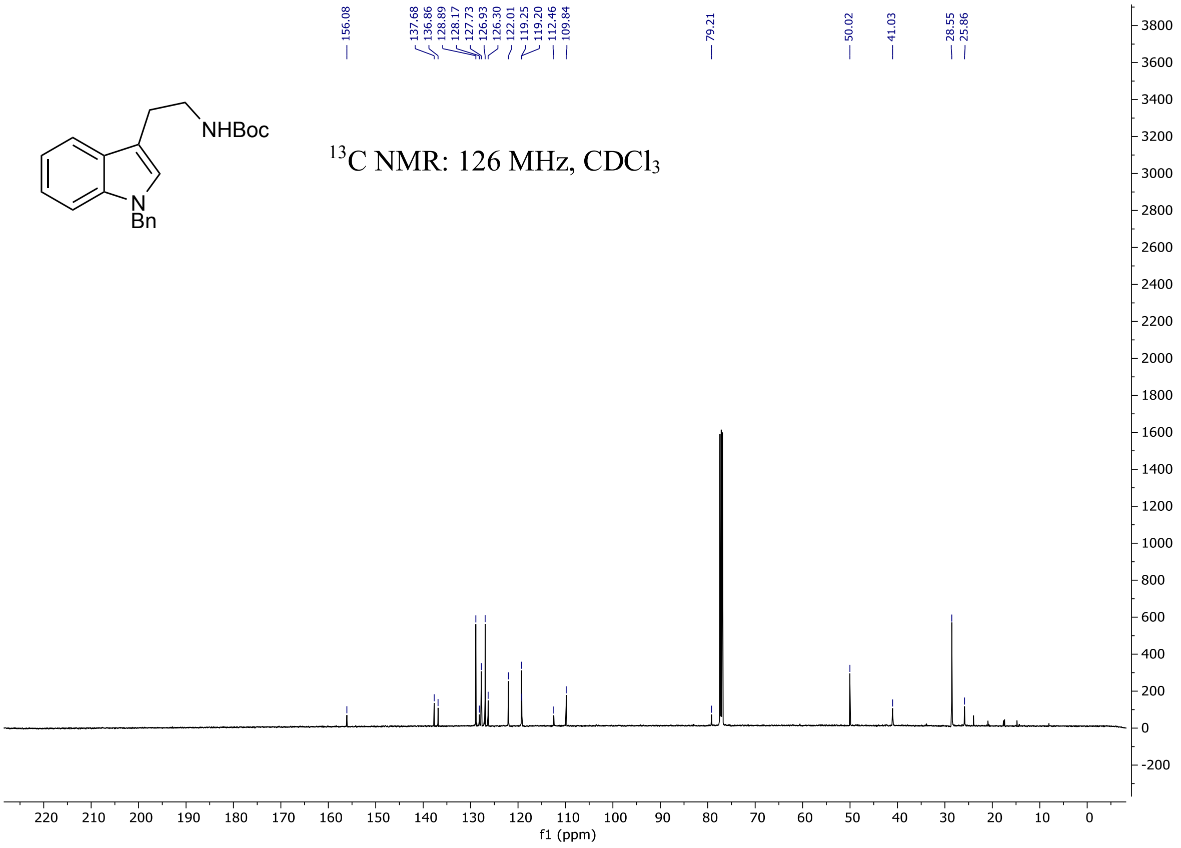
NHBoc
N
Bn
^{13}C NMR: 126 MHz, CDCl$_3$
f1 (ppm)

Compound 9

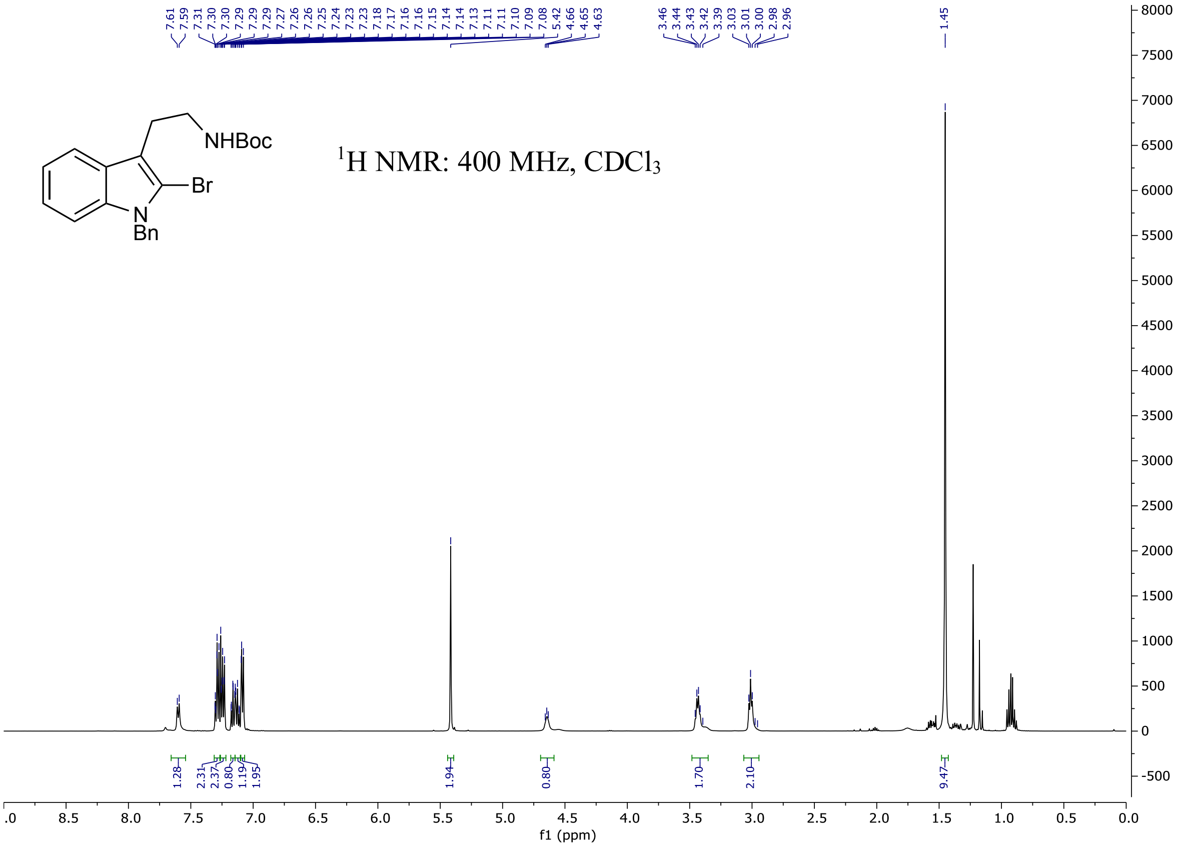

NHBoc
Br
N
Bn
^{1}H NMR: 400 MHz, CDCl$_3$
7.61 7.59 7.31 7.30 7.30 7.29 7.29 7.29 7.27 7.26 7.26 7.25 7.24 7.23 7.23 7.18 7.17 7.16 7.16 7.15 7.14 7.14 7.13 7.11 7.11 7.10 7.09 7.08 5.42 4.66 4.65 4.63
3.46 3.44 3.43 3.42 3.39 3.03 3.01 3.00 2.98 2.96
1.45
1.28 2.31 2.37 0.80 1.19 1.95 1.94 0.80 1.70 2.10 9.47
f1 (ppm)

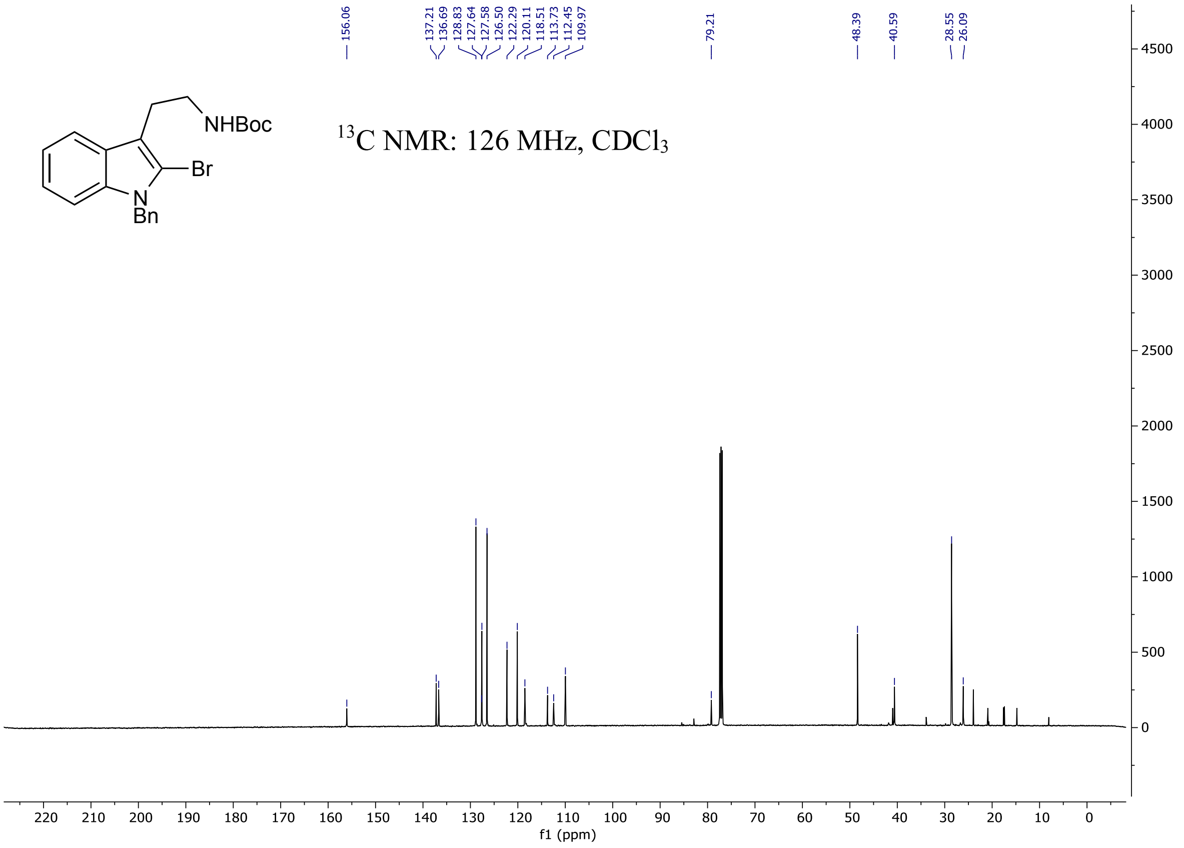

NHBoc
Br
N
Bn
^{13}C NMR: 126 MHz, CDCl$_3$
156.06
137.21 136.69 128.83 127.64 127.58 126.50 122.29 120.11 118.51 113.73 112.45 109.97
79.21
48.39 40.59 28.55 26.09
f1 (ppm)

Compound 10

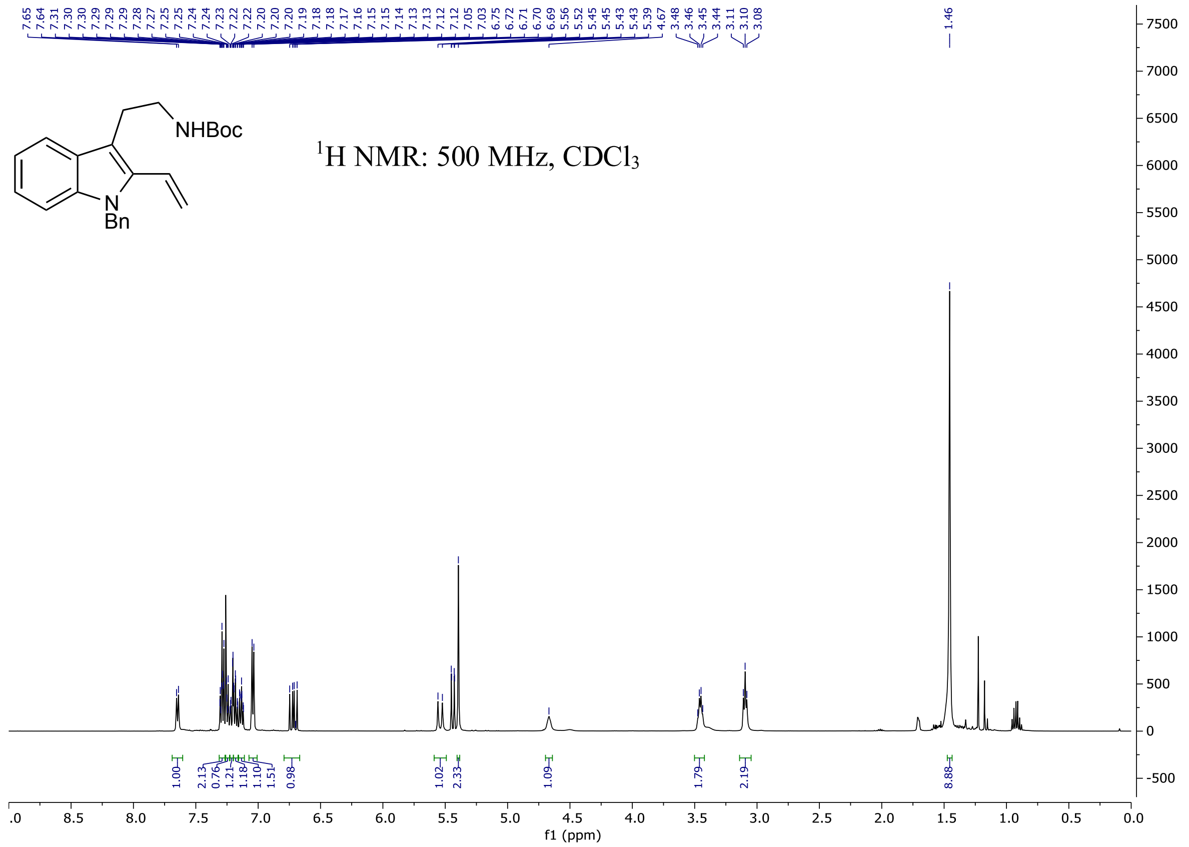
NHBoc
N
Bn
^{1}H NMR: 500 MHz, CDCl$_3$
f1 (ppm)

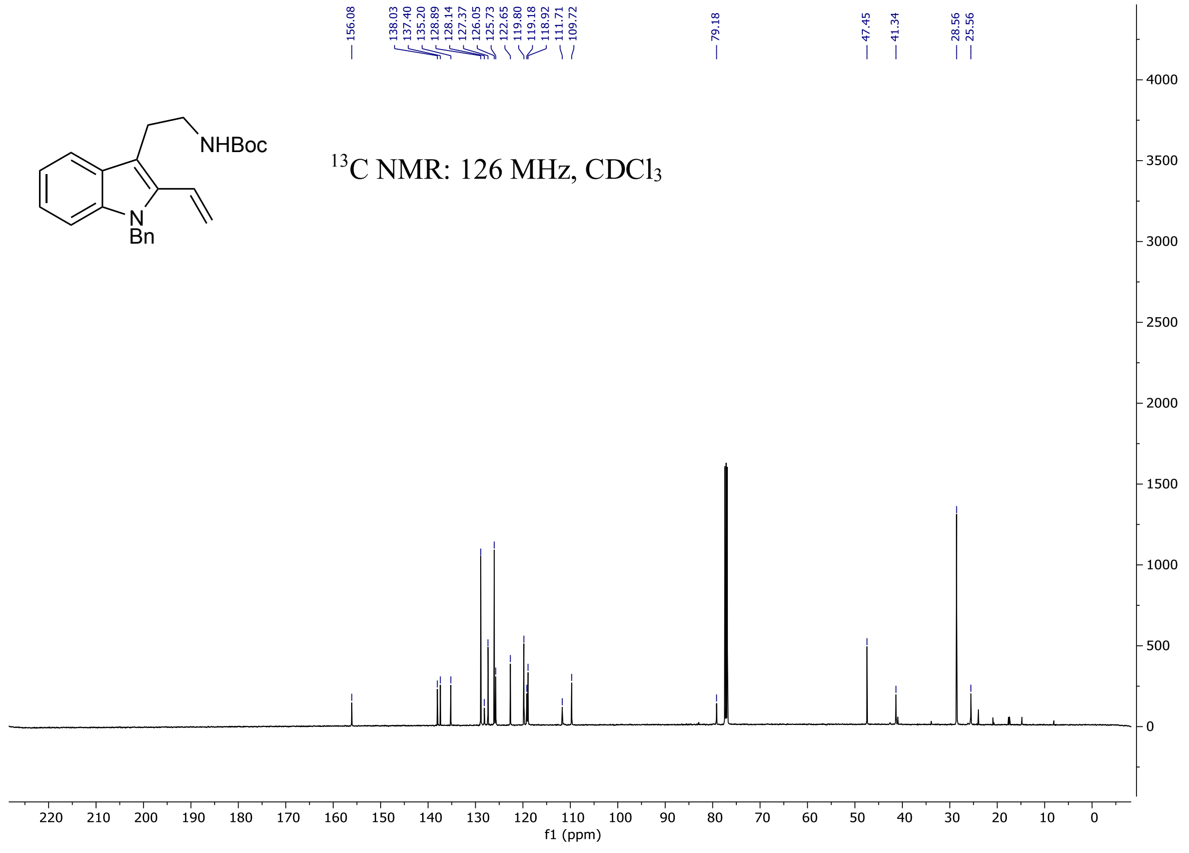
NHBoc
N
Bn
^{13}C NMR: 126 MHz, CDCl$_3$
f1 (ppm)

Compound 11

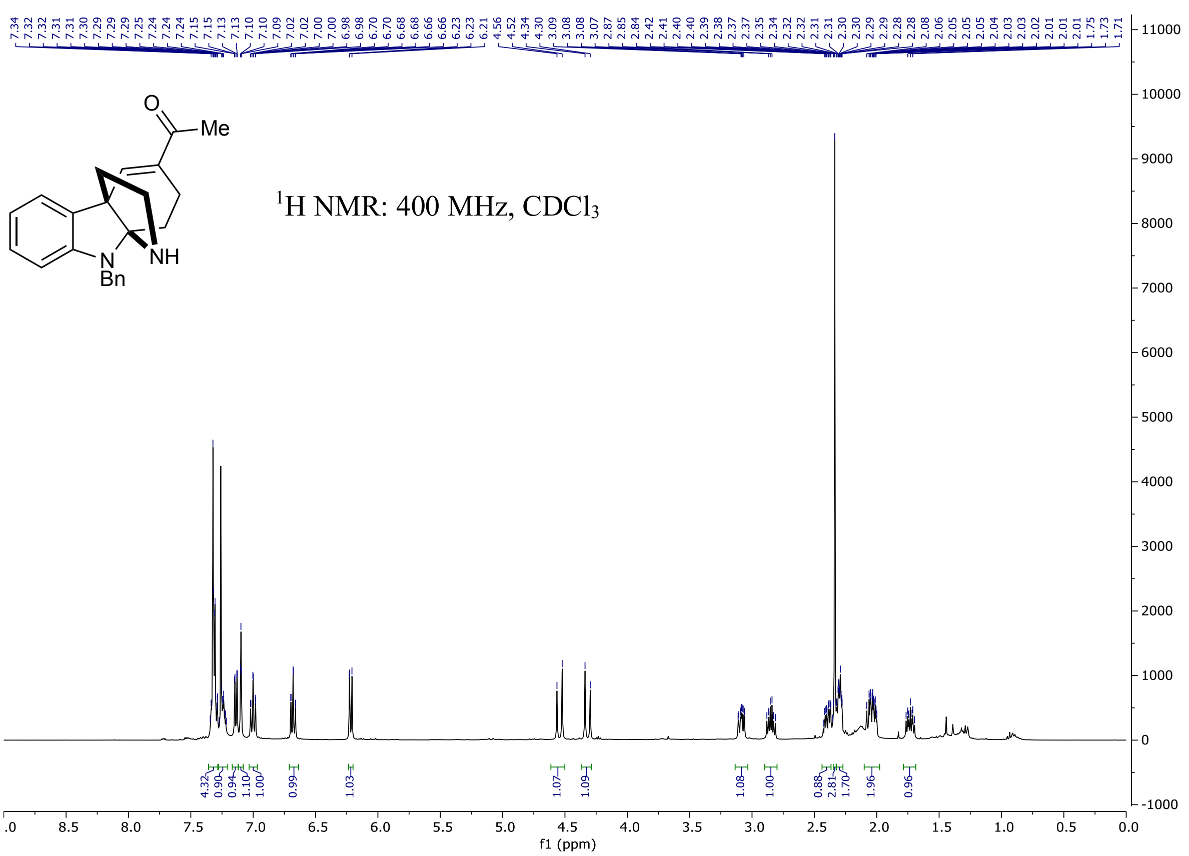

7.34 7.32 7.32 7.31 7.31 7.30 7.29 7.29 7.29 7.25 7.24 7.24 7.24 7.15 7.15 7.13 7.13 7.10 7.09 7.02 7.02 7.00 7.00 6.98 6.98 6.70 6.70 6.68 6.68 6.66 6.66 6.23 6.21 4.56 4.52 4.34 4.30 3.09 3.08 3.08 3.07 2.87 2.85 2.84 2.42 2.41 2.40 2.40 2.39 2.38 2.37 2.37 2.35 2.34 2.32 2.32 2.31 2.31 2.30 2.30 2.29 2.29 2.28 2.28 2.08 2.06 2.05 2.05 2.05 2.04 2.03 2.03 2.02 2.01 2.01 2.01 1.75 1.73 1.71
O
Me
N
NH
Bn
^{1}H NMR: 400 MHz, CDCl$_3$
4.32 0.90 0.94 1.10 1.00 0.99 1.03 1.07 1.09 1.08 1.00 0.88 2.81 1.70 1.96 0.96
f1 (ppm)

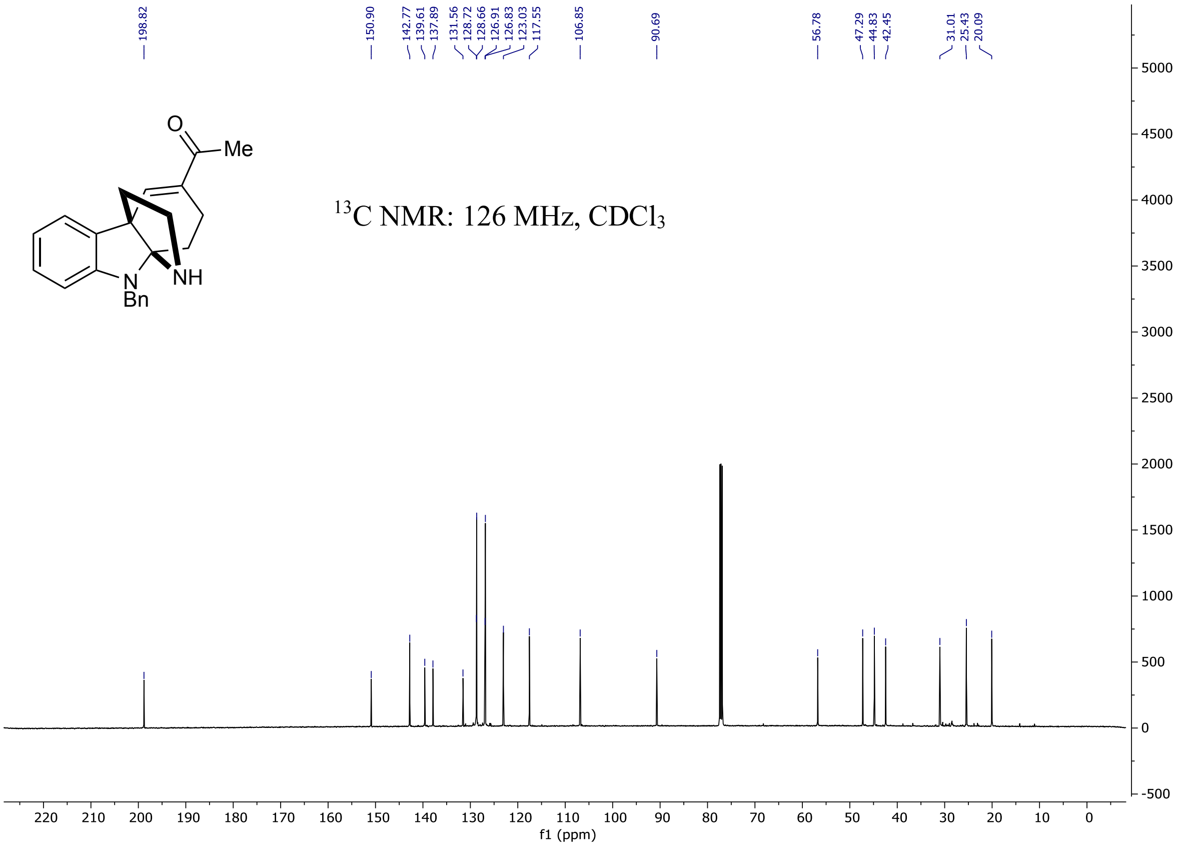

198.82 150.90 142.77 139.61 137.89 131.56 128.72 128.66 126.91 126.83 123.03 117.55 106.85 90.69 56.78 47.29 44.83 42.45 31.01 25.43 20.09
O
Me
N
NH
Bn
^{13}C NMR: 126 MHz, CDCl$_3$
f1 (ppm)

Compound 12

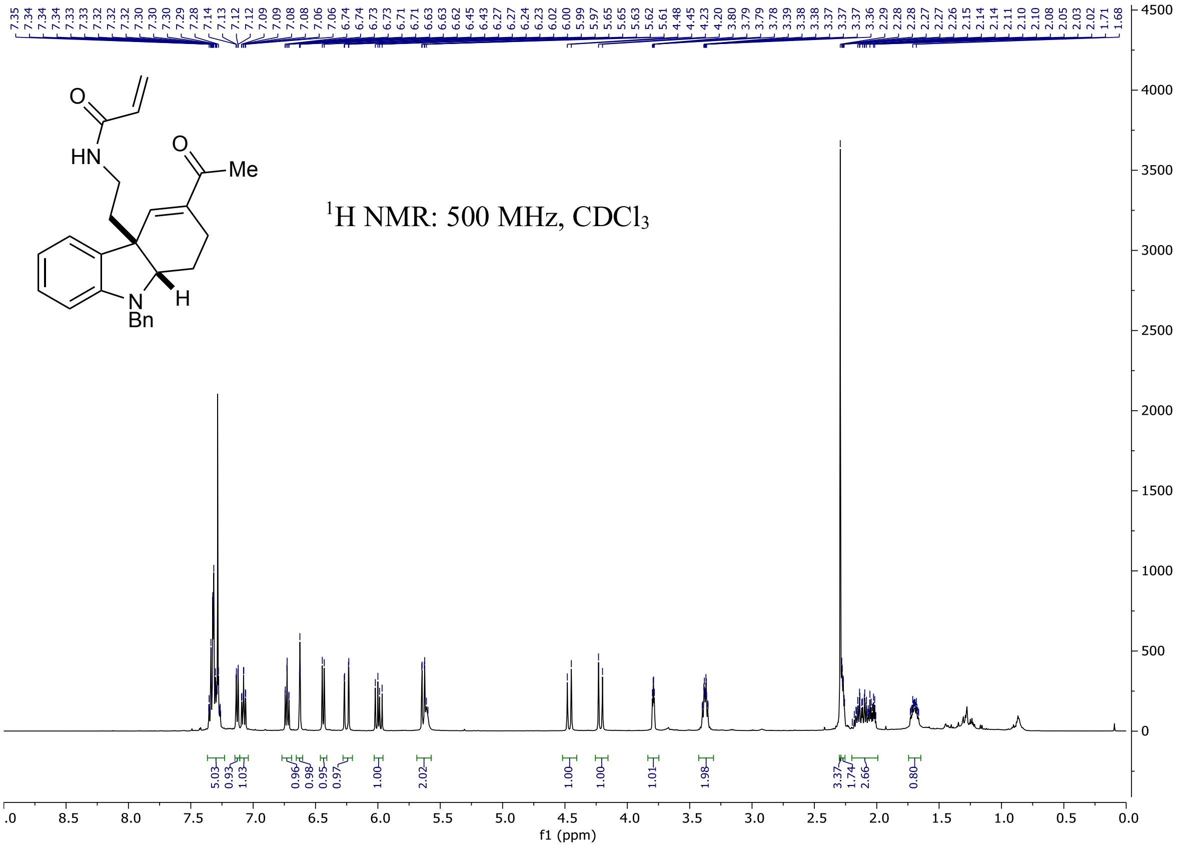

HN
O
O
Me
N
Bn
H
^{1}H NMR: 500 MHz, CDCl$_3$
7.35 7.34 7.34 7.34 7.33 7.33 7.32 7.32 7.32 7.30 7.30 7.30 7.29 7.28 7.14 7.13 7.12 7.12 7.09 7.09 7.08 7.08 7.06 7.06 6.74 6.74 6.73 6.73 6.71 6.71 6.63 6.63 6.62 6.45 6.43 6.27 6.27 6.24 6.23 6.02 6.00 5.99 5.97 5.65 5.65 5.63 5.62 5.61 4.48 4.45 4.23 4.20 3.80 3.79 3.79 3.78 3.39 3.38 3.38 3.37 3.37 3.37 3.36 2.29 2.28 2.28 2.27 2.27 2.26 2.15 2.14 2.14 2.11 2.10 2.10 2.08 2.05 2.03 2.02 1.71 1.68
5.03 0.93 1.03 0.96 0.98 0.95 0.97 1.00 2.02 1.00 1.00 1.01 1.98 3.37 1.74 2.66 0.80
f1 (ppm)

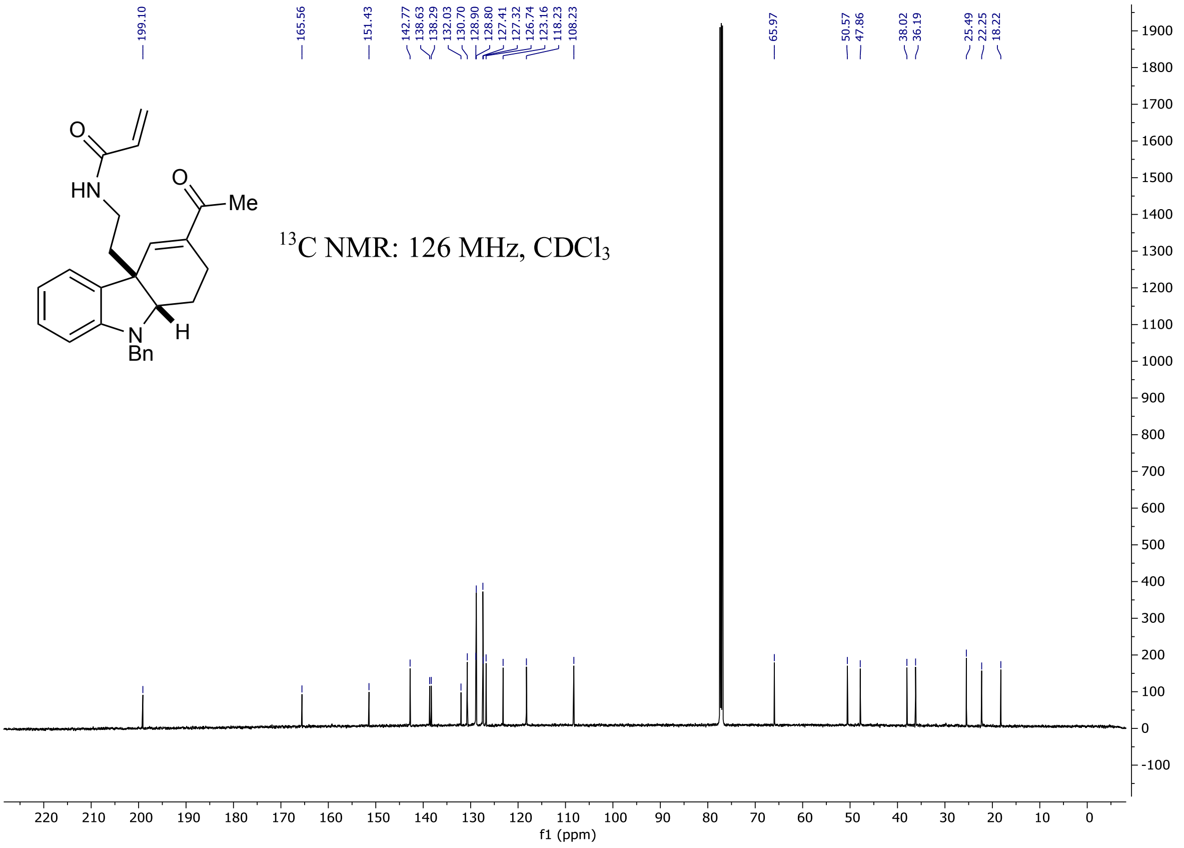

HN
O
O
Me
N
Bn
H
^{13}C NMR: 126 MHz, CDCl$_3$
199.10 165.56 151.43 142.77 138.63 138.29 132.03 130.70 128.90 128.80 127.41 127.32 126.74 123.16 118.23 108.23 65.97 50.57 47.86 38.02 36.19 25.49 22.25 18.22
f1 (ppm)

Compound 13

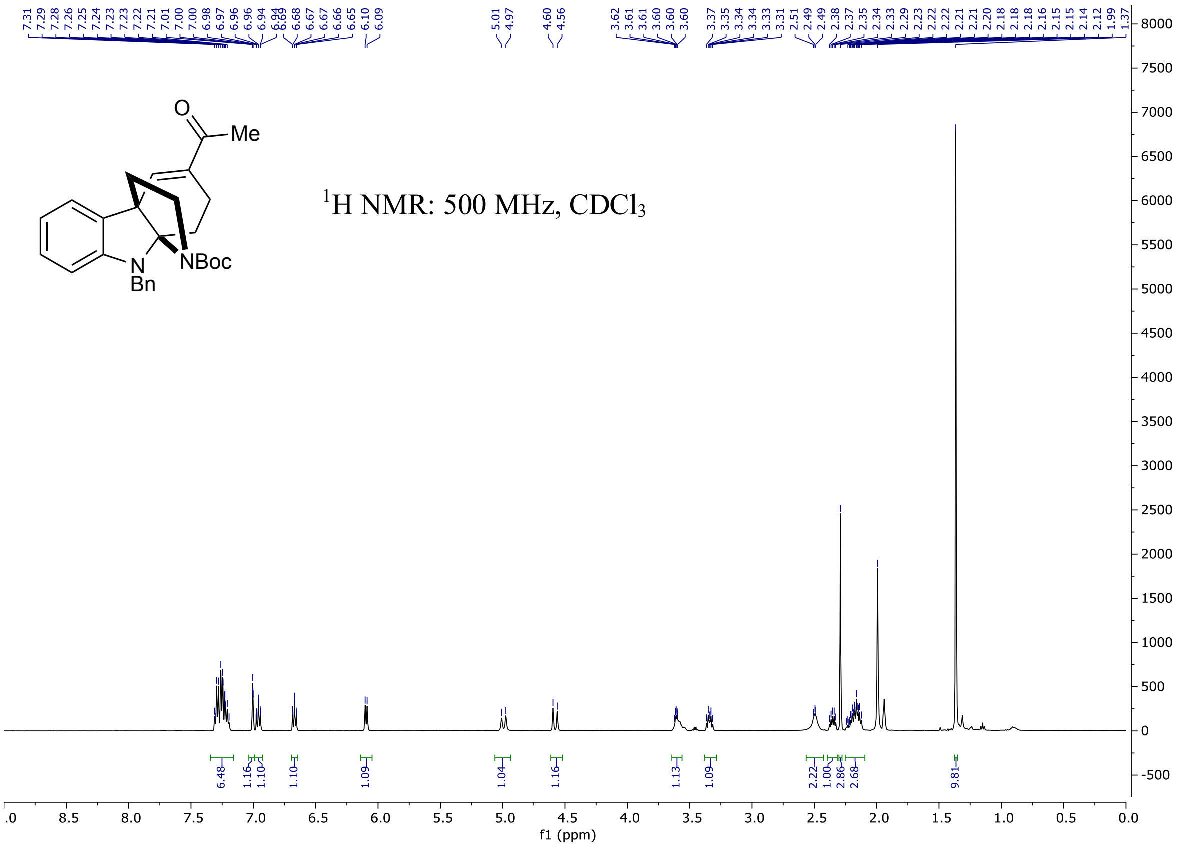
O
Me
N
Bn
NBoc
^{1}H NMR: 500 MHz, CDCl$_3$
f1 (ppm)

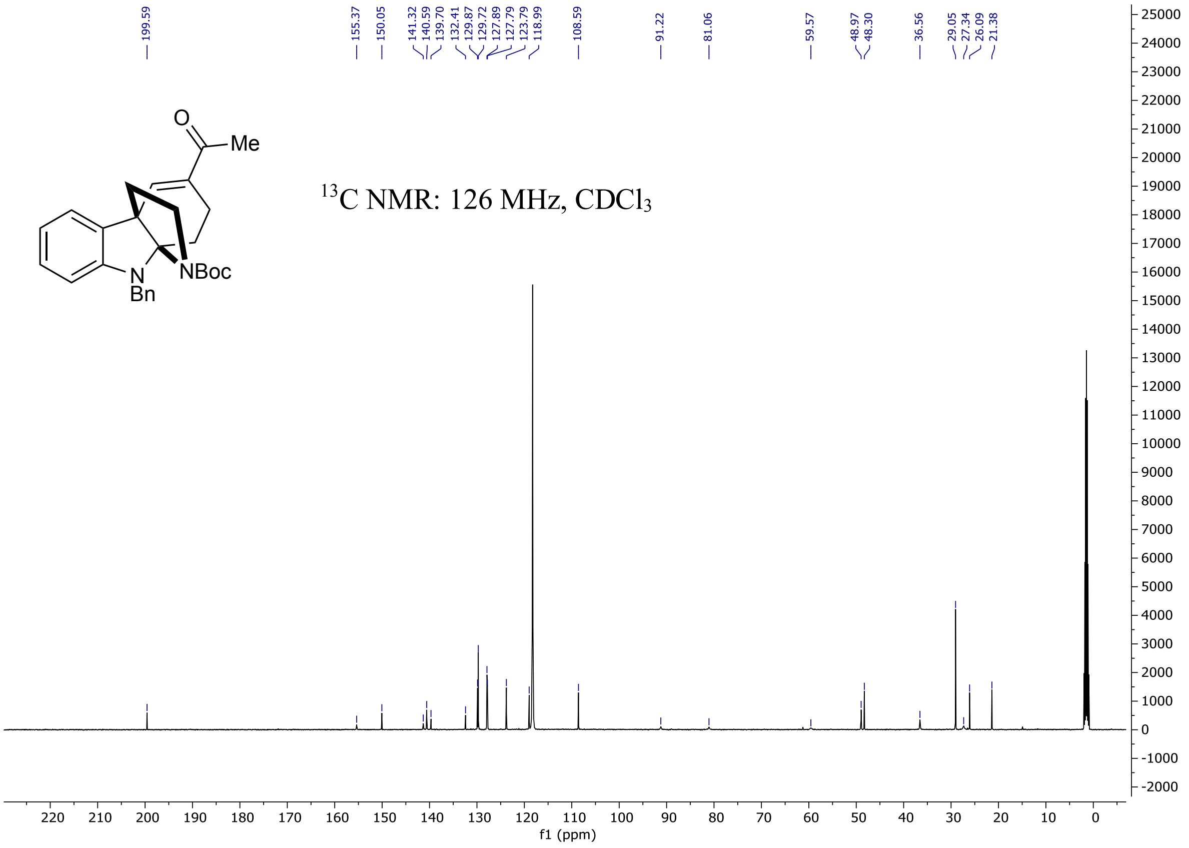
O
Me
N
Bn
NBoc
^{13}C NMR: 126 MHz, CDCl$_3$
f1 (ppm)

Compound 14

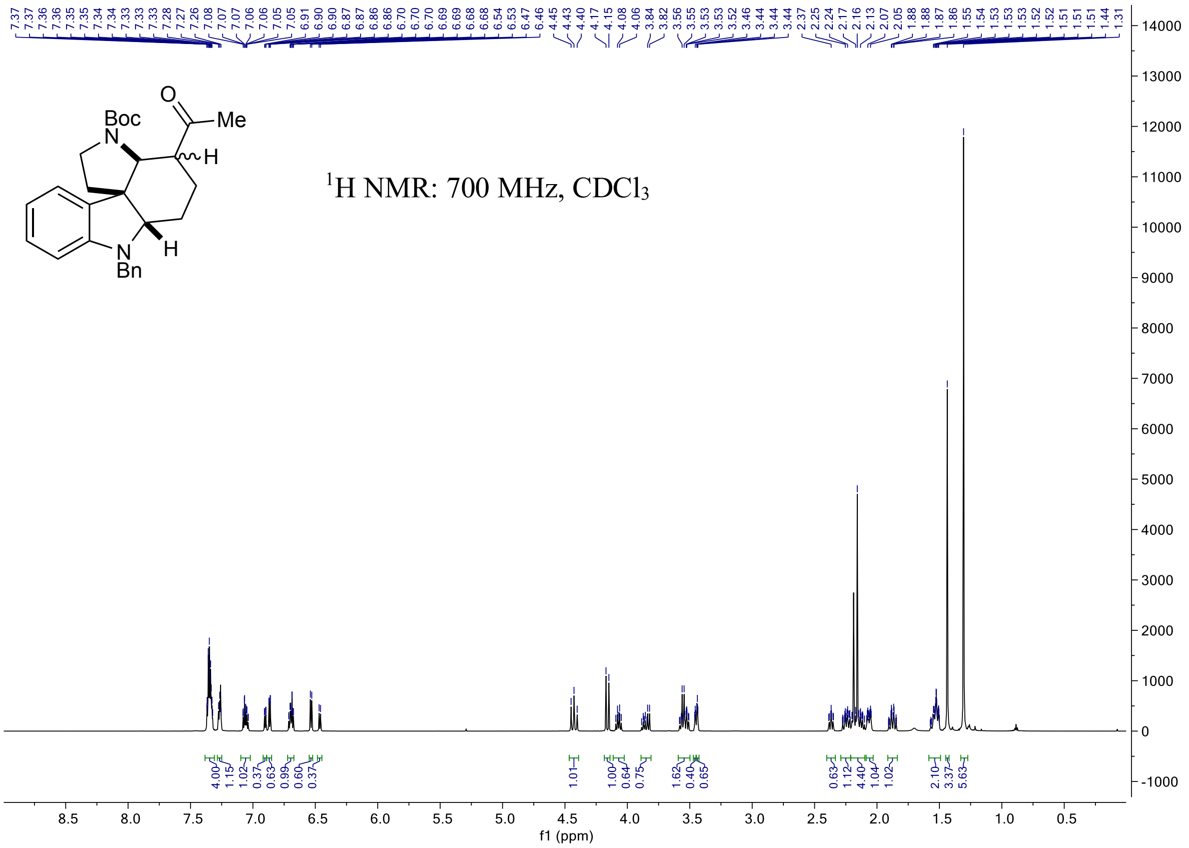

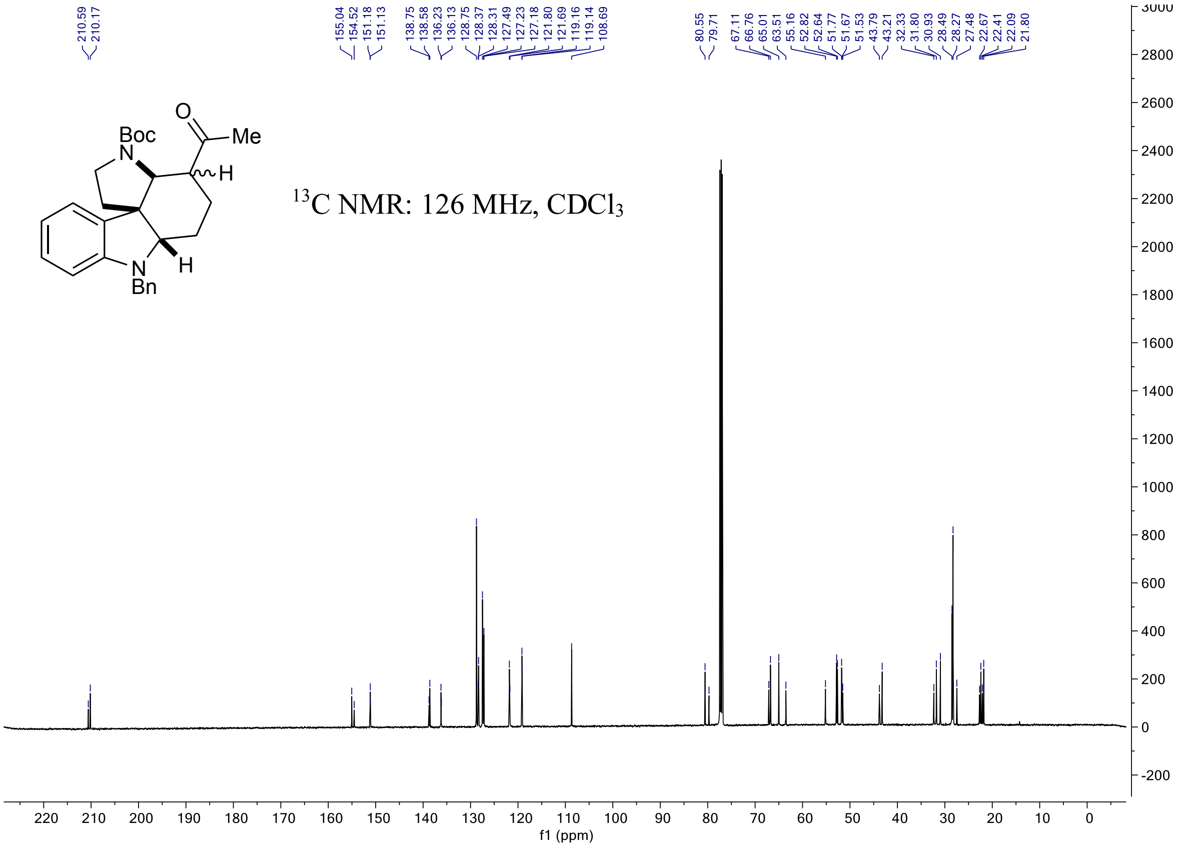

Compound 16

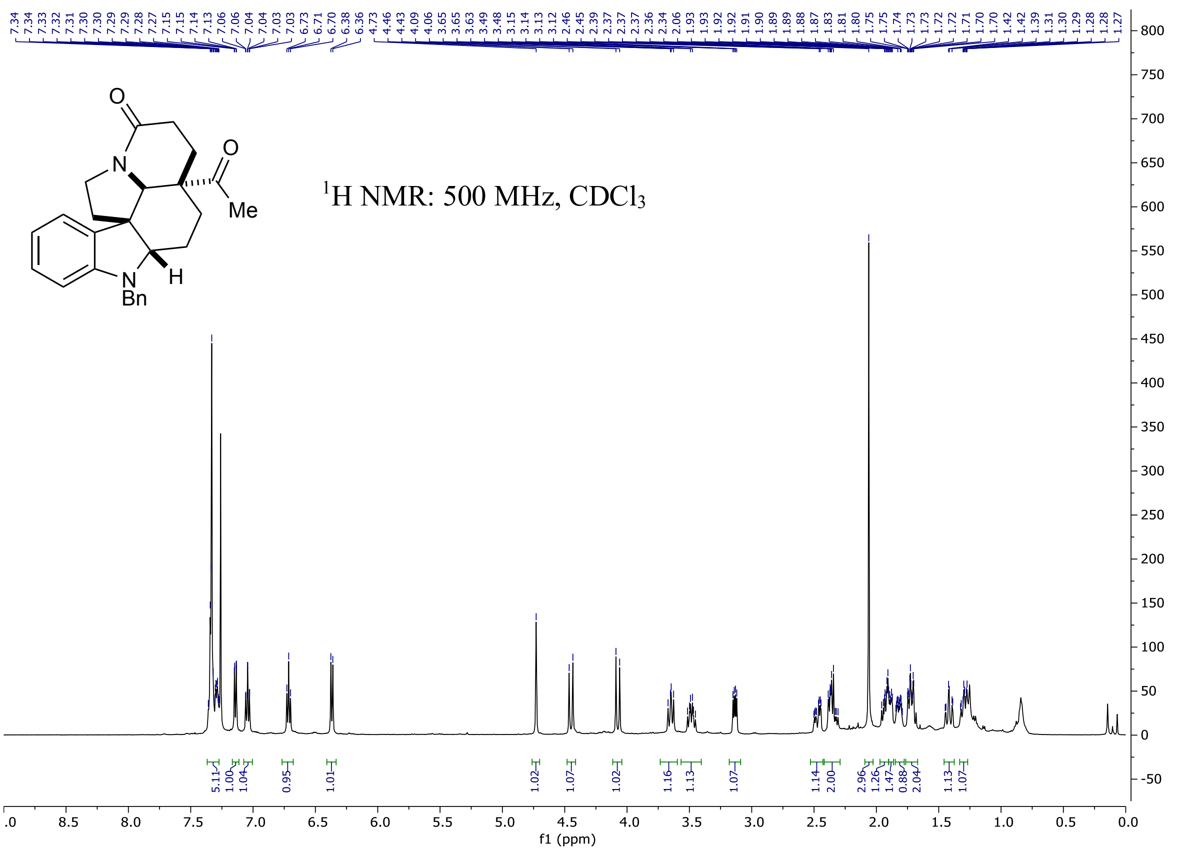

O
N
O
Me
N
Bn
H
^{1}H NMR: 500 MHz, CDCl$_3$
f1 (ppm)

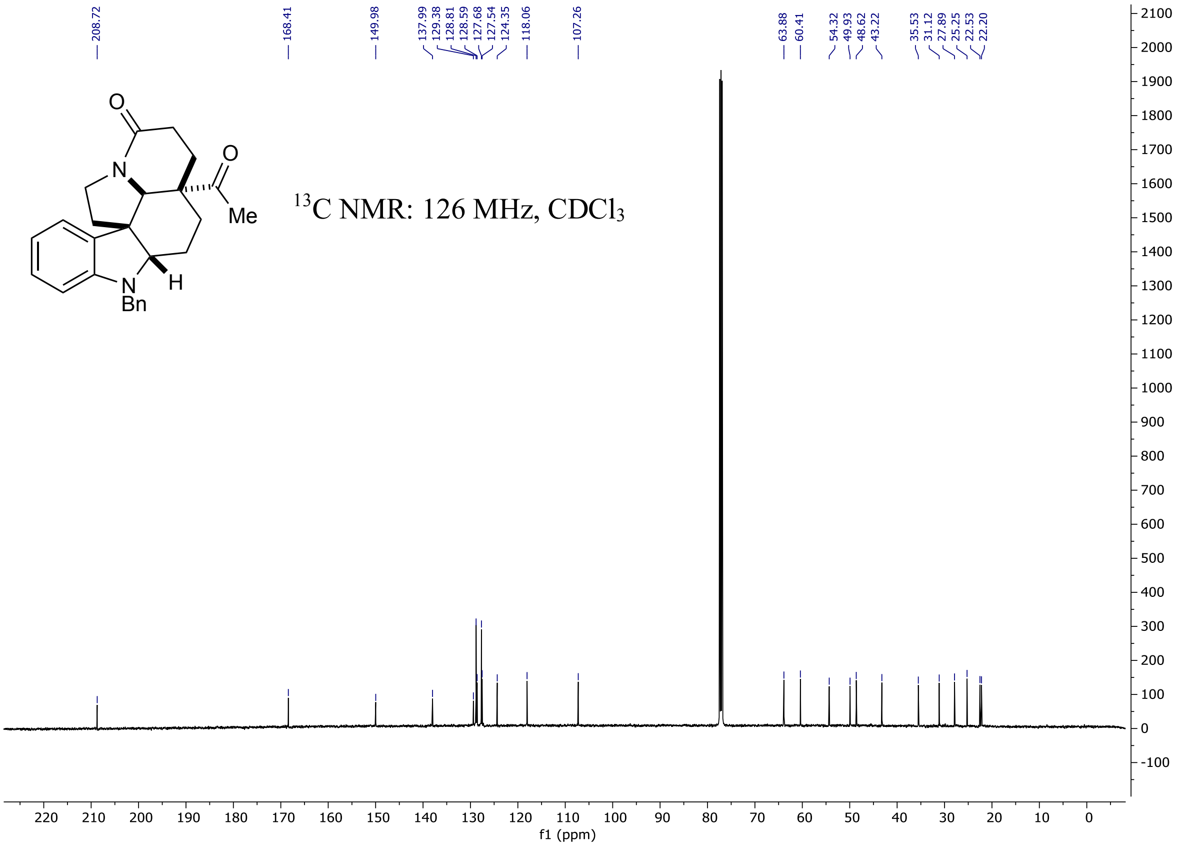

O
N
O
Me
N
Bn
H
^{13}C NMR: 126 MHz, CDCl$_3$
208.72
168.41
149.98
137.99
129.38
128.81
128.59
127.68
127.54
124.35
118.06
107.26
63.88
60.41
54.32
49.93
48.62
43.22
35.53
31.12
27.89
25.25
22.53
22.20
f1 (ppm)

Compound 17

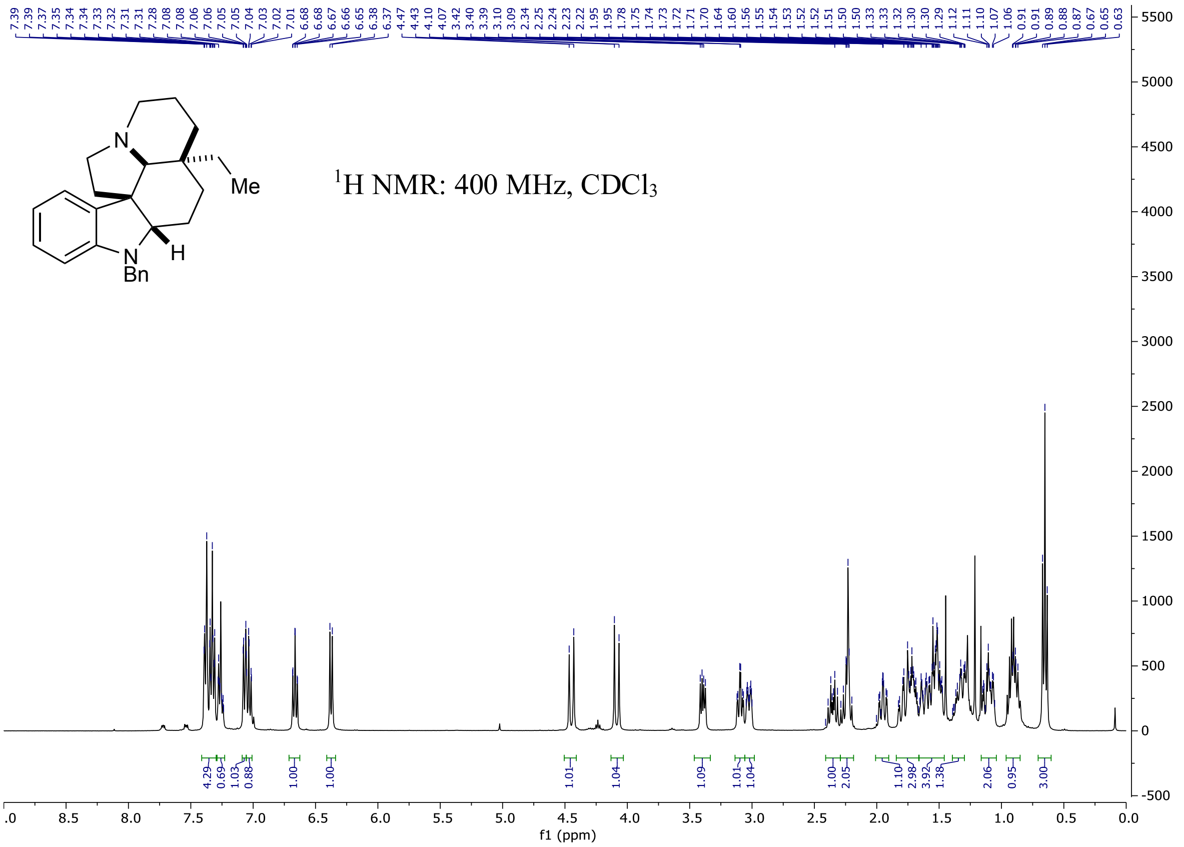
^{1}H NMR: 400 MHz, CDCl$_3$
Me
N
Bn
H
f1 (ppm)

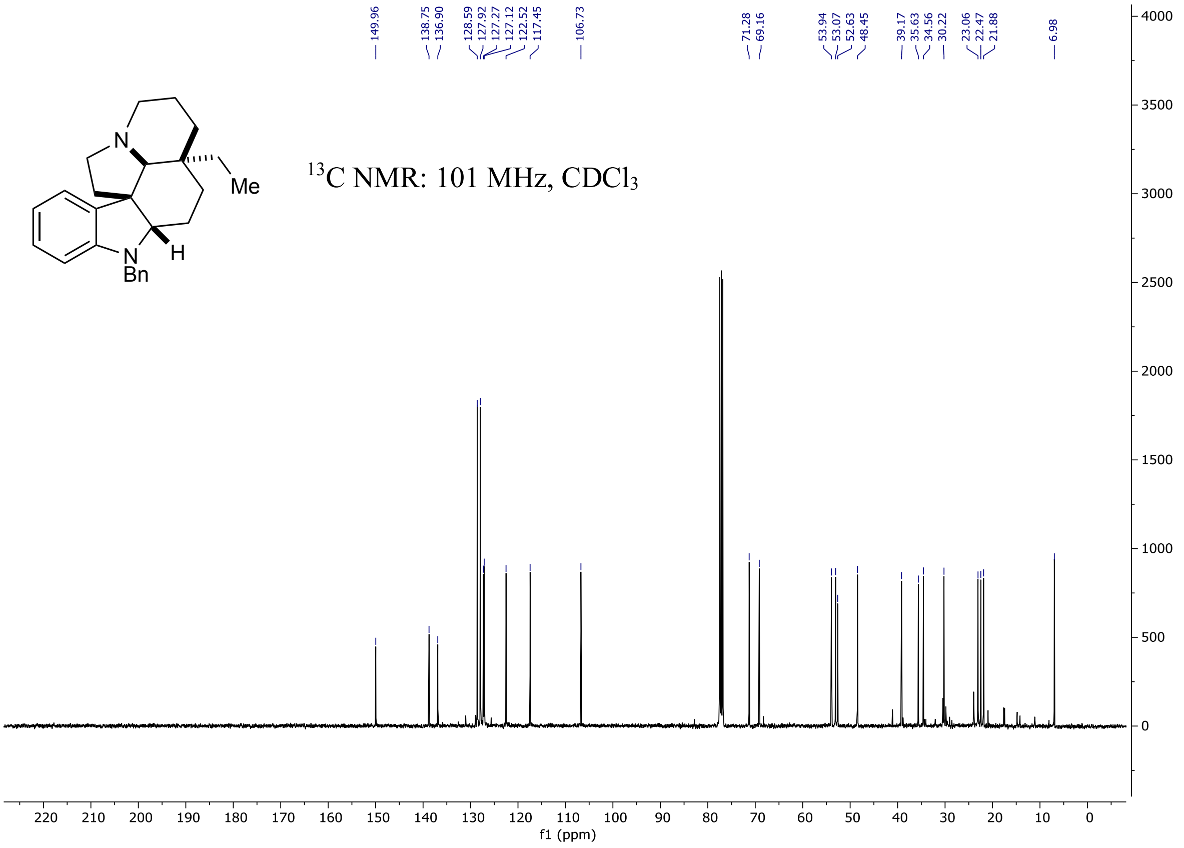
^{13}C NMR: 101 MHz, CDCl$_3$
Me
N
Bn
H
f1 (ppm)

Compound 18

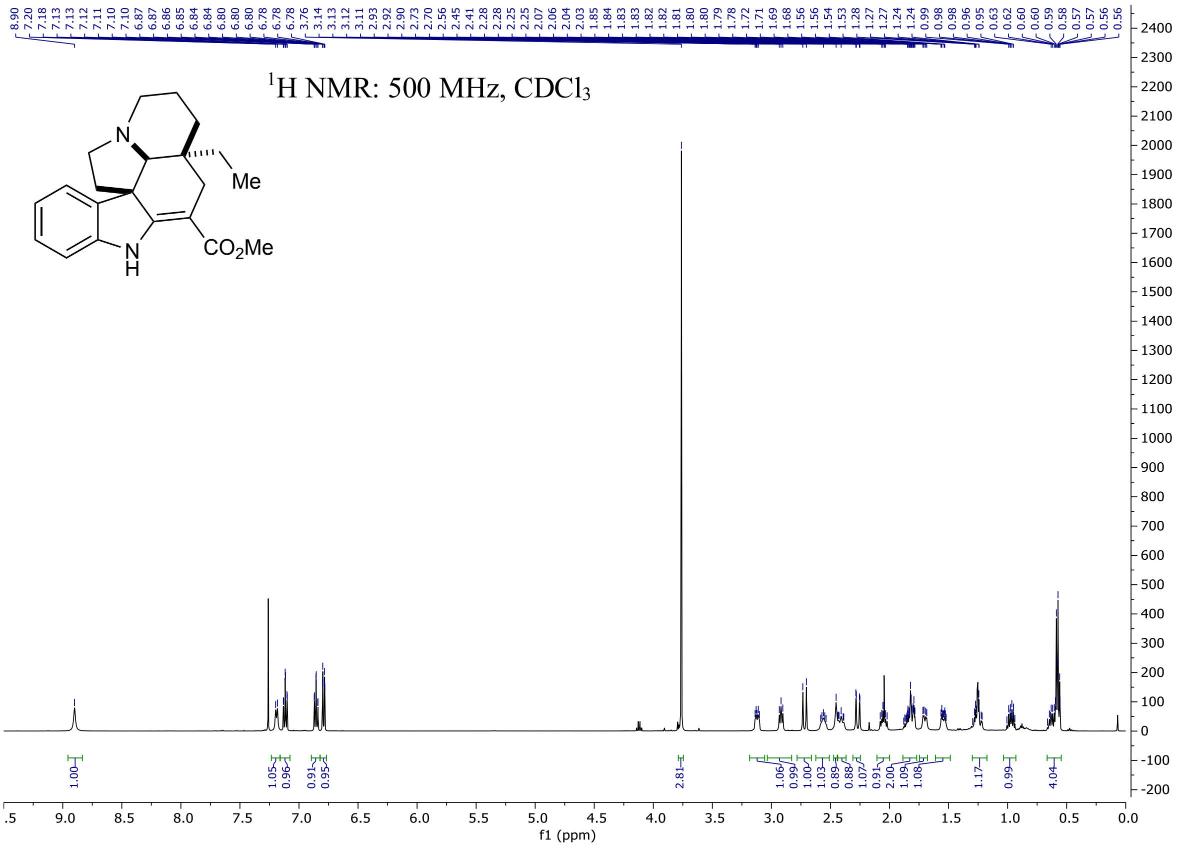

^{1}H NMR: 500 MHz, CDCl$_3$
Me
N
N
H
CO$_2$Me
f1 (ppm)

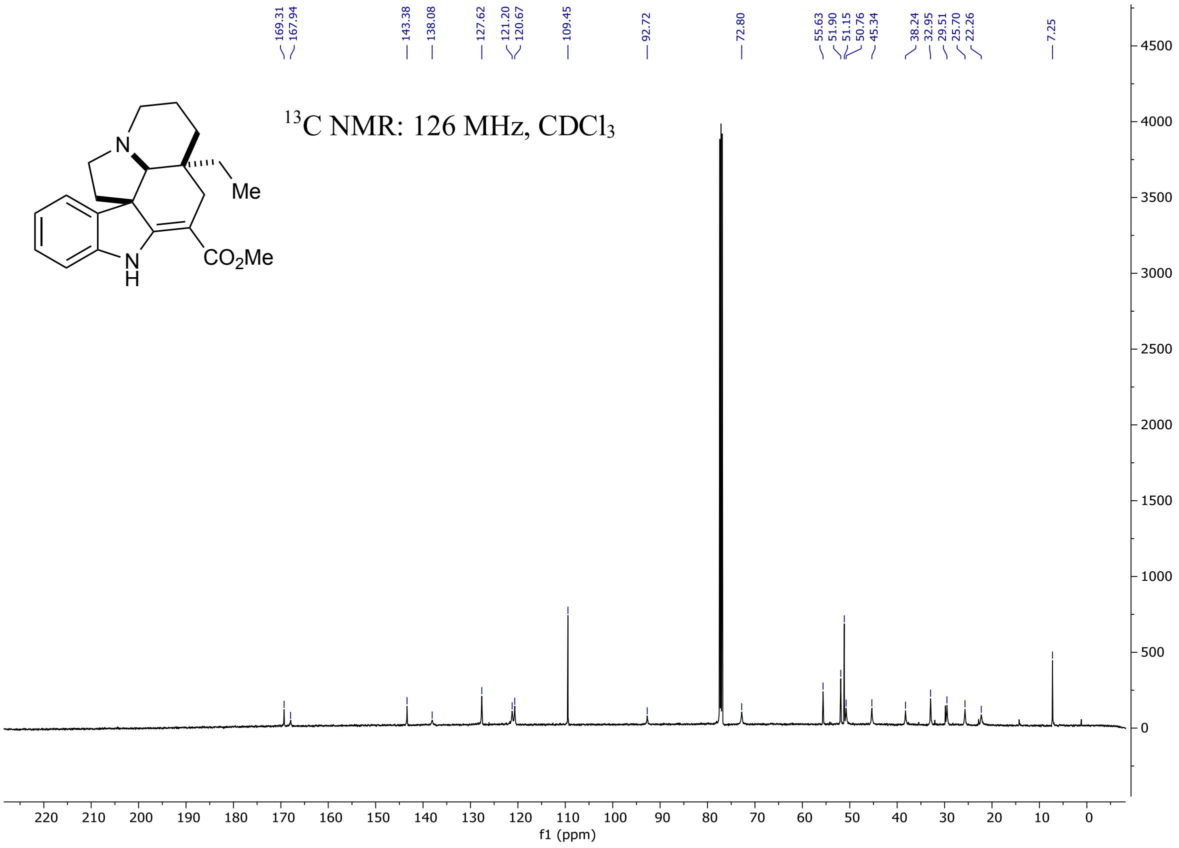

^{13}C NMR: 126 MHz, CDCl$_3$
Me
N
N
H
CO$_2$Me
f1 (ppm)

Compound 19

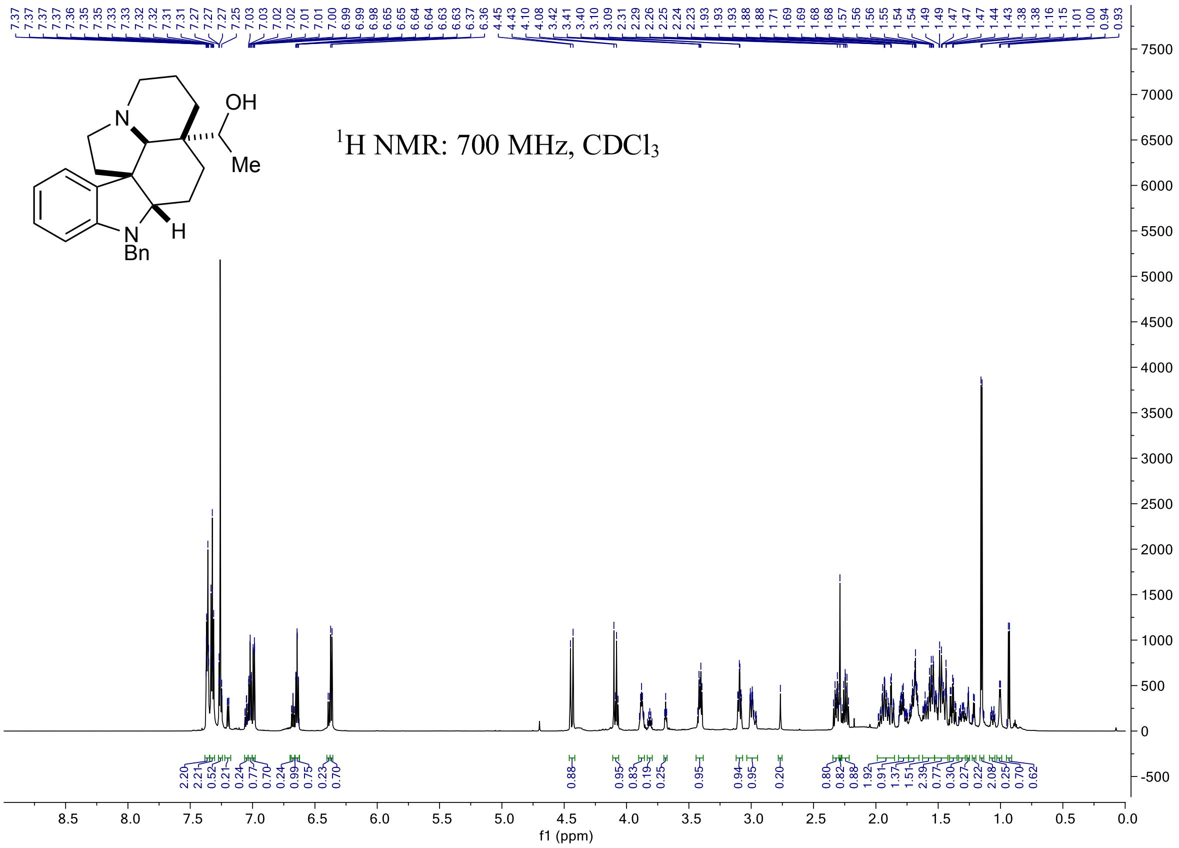

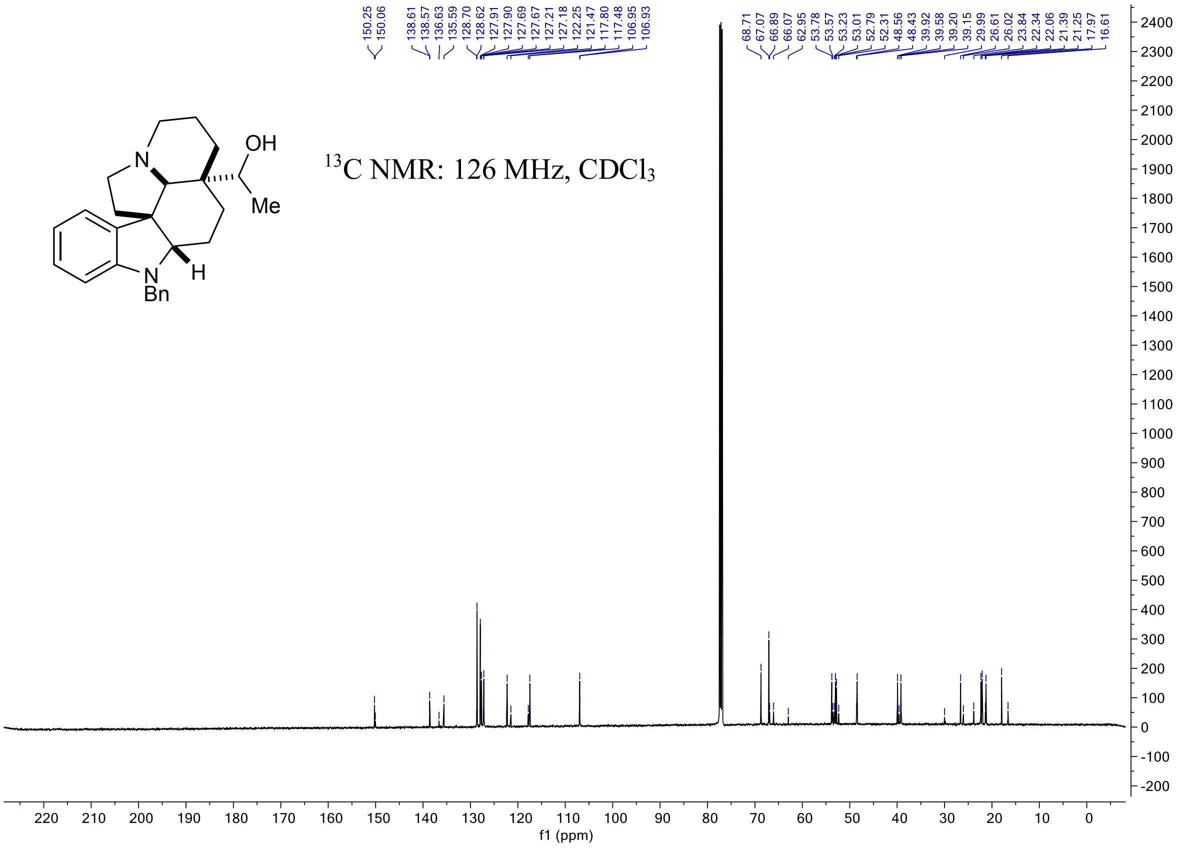

Compound 20

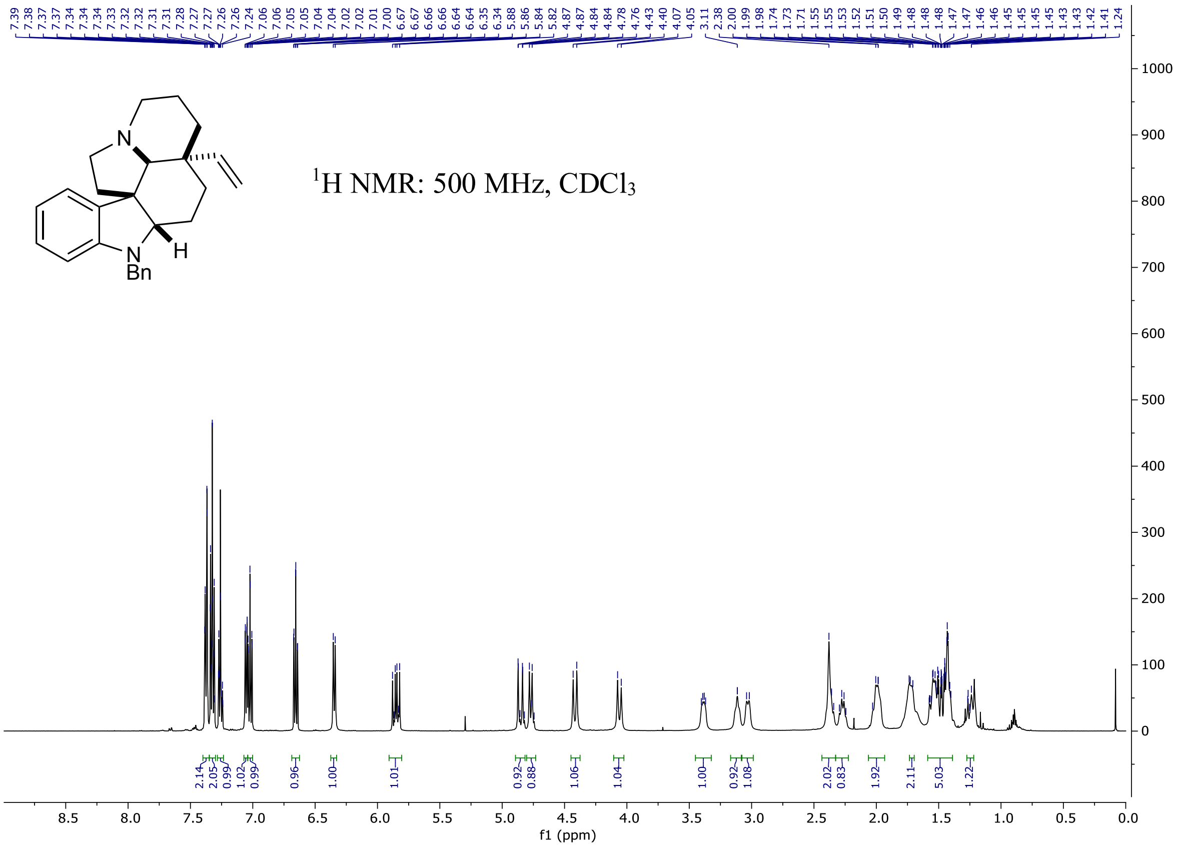
7.39 7.38 7.37 7.37 7.34 7.34 7.33 7.32 7.32 7.31 7.31 7.28 7.27 7.27 7.26 7.26 7.24 7.06 7.05 7.05 7.04 7.04 7.02 7.02 7.01 7.00 6.67 6.67 6.66 6.66 6.64 6.64 6.35 6.34 5.88 5.86 5.84 5.82 4.87 4.87 4.84 4.84 4.78 4.76 4.43 4.40 4.07 4.05 3.11 2.38 2.00 1.99 1.98 1.74 1.73 1.71 1.55 1.55 1.53 1.52 1.51 1.50 1.49 1.48 1.48 1.48 1.47 1.46 1.45 1.45 1.45 1.43 1.42 1.41 1.24
N
H
N
Bn
^{1}H NMR: 500 MHz, CDCl$_3$
2.14 2.05 0.99 1.02 0.99 0.96 1.00 1.01 0.92 0.88 1.06 1.04 1.00 0.92 1.08 2.02 0.83 1.92 2.11 5.03 1.22
f1 (ppm)

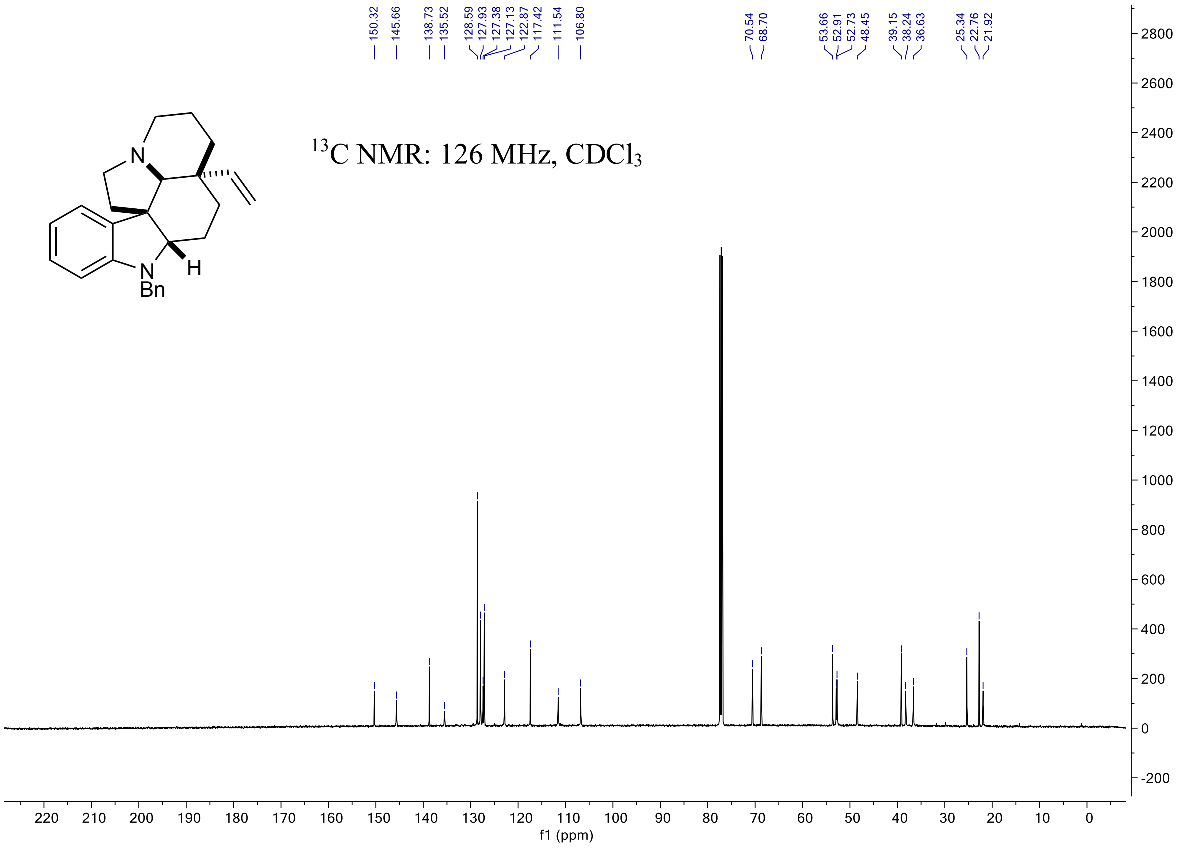
150.32 145.66 138.73 135.52 128.59 127.93 127.38 127.13 122.87 117.42 111.54 106.80 70.54 68.70 53.66 52.91 52.73 48.45 39.15 38.24 36.63 25.34 22.76 21.92
N
H
N
Bn
^{13}C NMR: 126 MHz, CDCl$_3$
f1 (ppm)

Compound 21

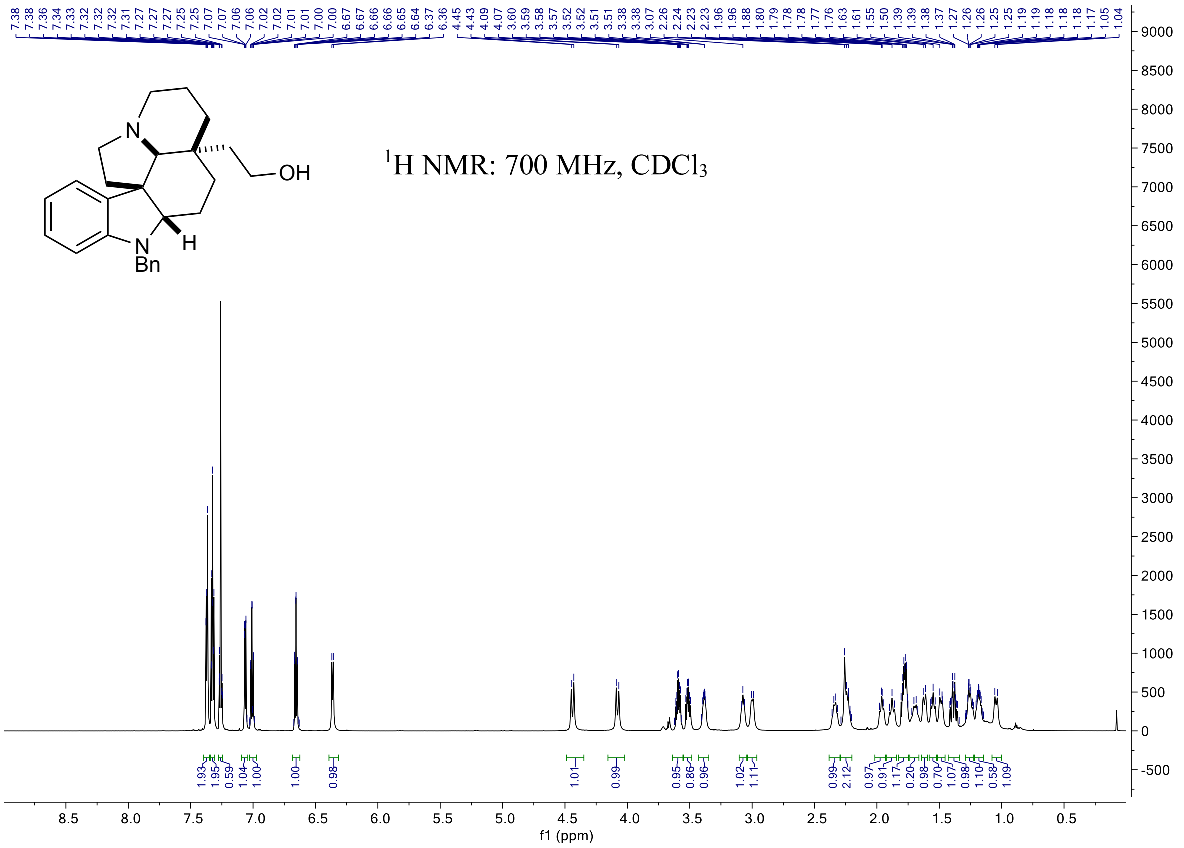

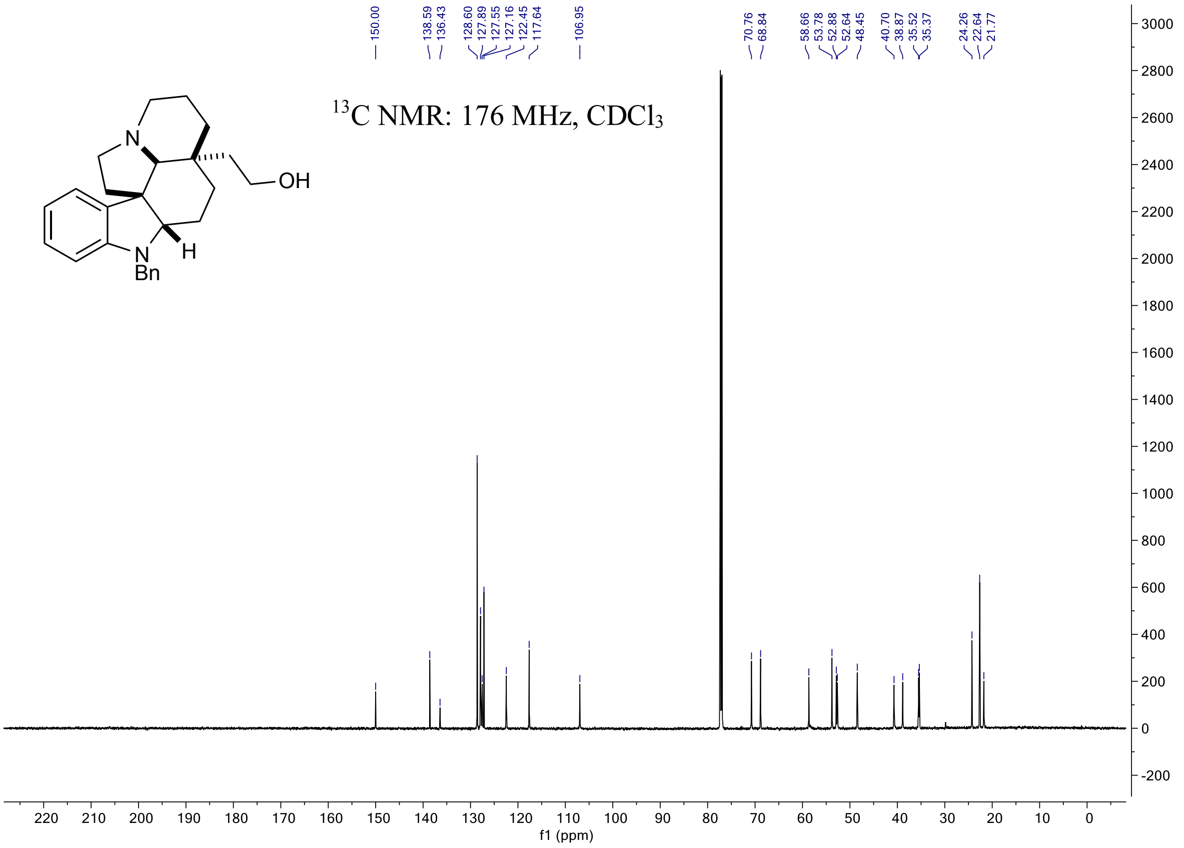

Compound 22

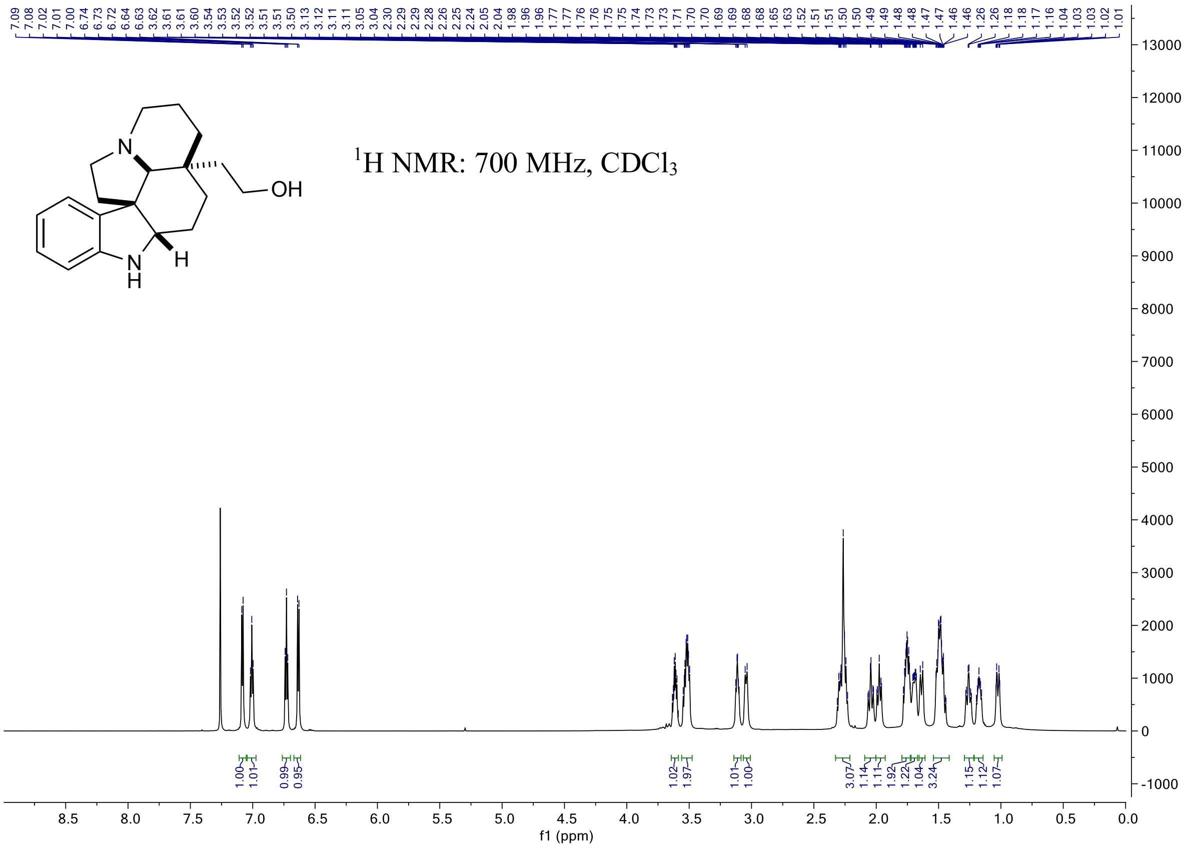

N
OH
N
H
H
^{1}H NMR: 700 MHz, CDCl$_3$
f1 (ppm)

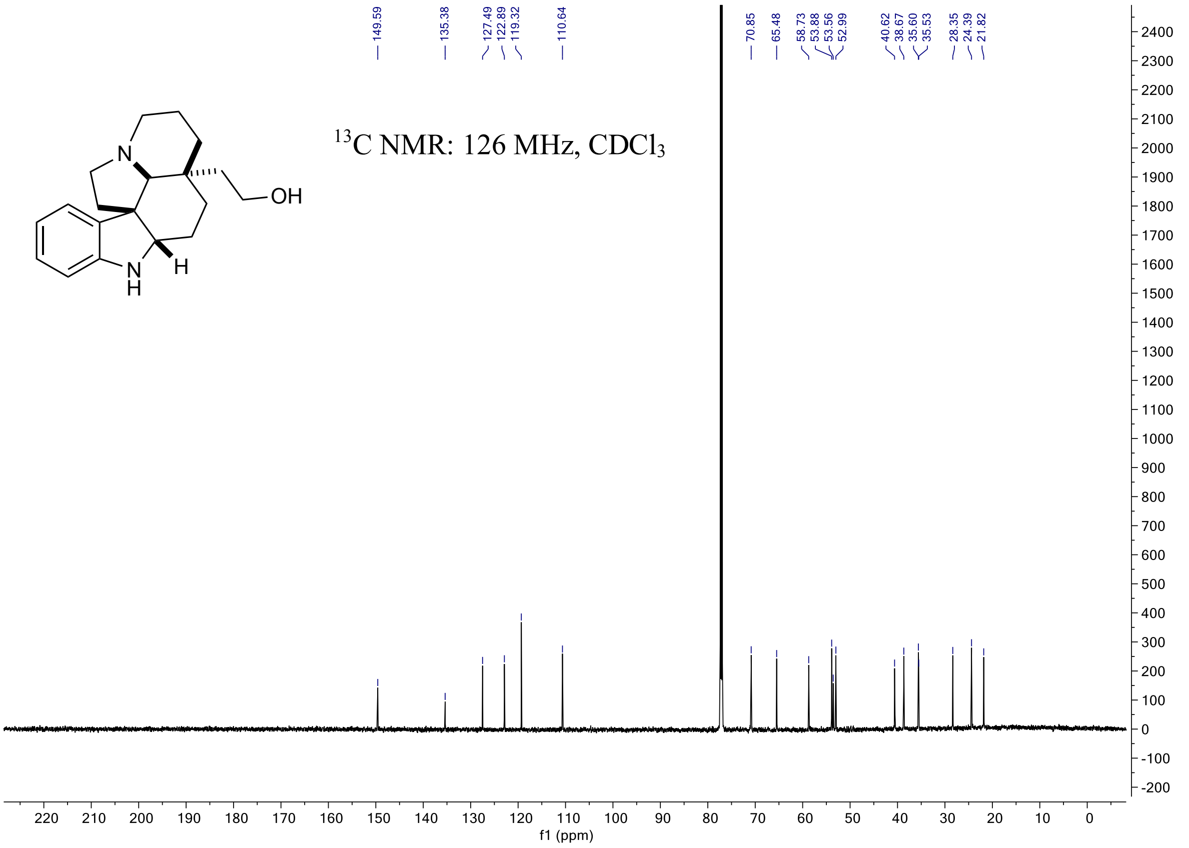

149.59
135.38
127.49
122.89
119.32
110.64
70.85
65.48
58.73
53.88
53.56
52.99
40.62
38.67
35.60
35.53
28.35
24.39
21.82
N
OH
N
H
H
^{13}C NMR: 126 MHz, CDCl$_3$
f1 (ppm)

Compound 23

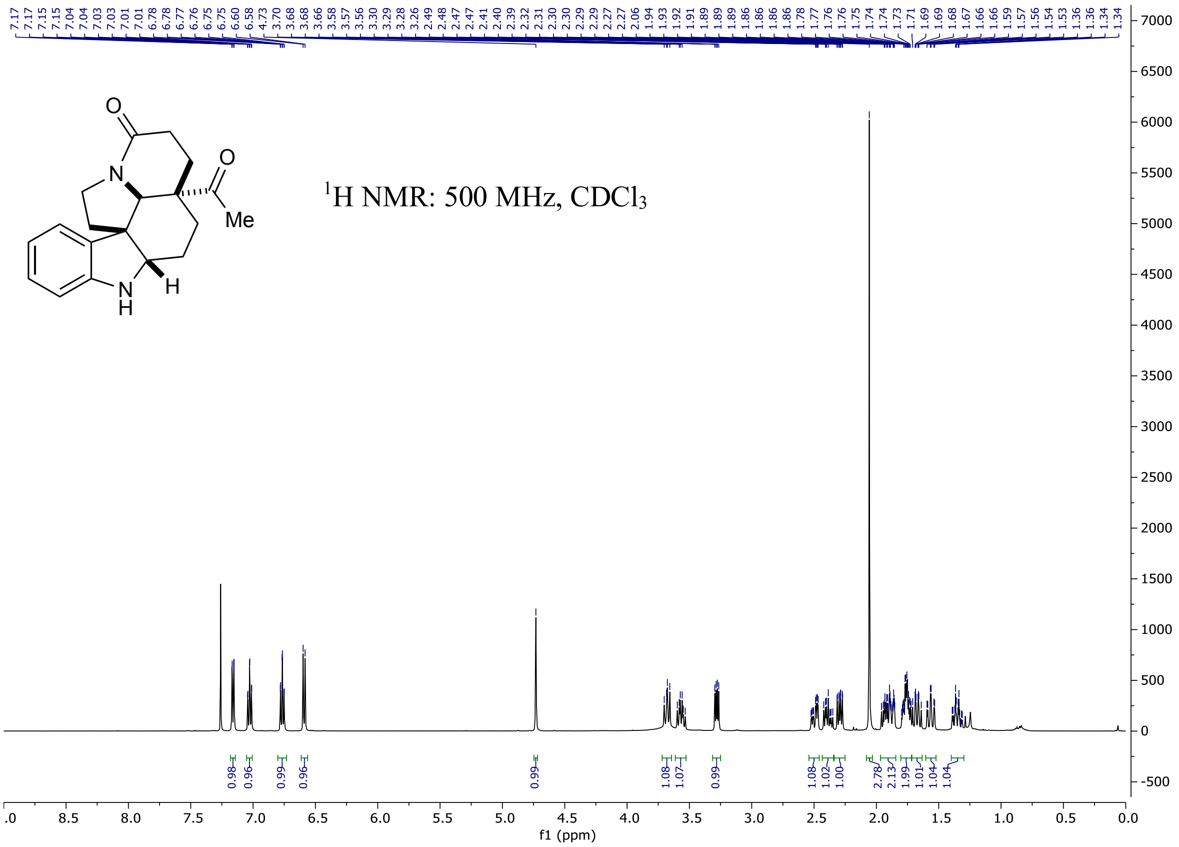

7.17
7.17
7.15
7.15
7.04
7.04
7.03
7.03
7.01
7.01
6.78
6.78
6.77
6.76
6.75
6.75
6.60
6.58
4.73
3.70
3.68
3.68
3.66
3.58
3.57
3.56
3.30
3.29
3.28
3.26
2.49
2.48
2.47
2.47
2.41
2.40
2.39
2.32
2.31
2.30
2.30
2.29
2.29
2.27
2.27
2.06
1.94
1.93
1.92
1.91
1.89
1.89
1.89
1.86
1.86
1.86
1.86
1.78
1.77
1.76
1.76
1.75
1.75
1.74
1.74
1.73
1.71
1.69
1.69
1.68
1.67
1.66
1.66
1.59
1.57
1.56
1.54
1.53
1.36
1.36
1.34
1.34
O
N
O
Me
N
H
H
^{1}H NMR: 500 MHz, CDCl3
0.98
0.96
0.99
0.96
0.99
1.08
1.07
0.99
1.08
1.02
1.00
2.78
2.13
1.99
1.01
1.04
1.04
f1 (ppm)

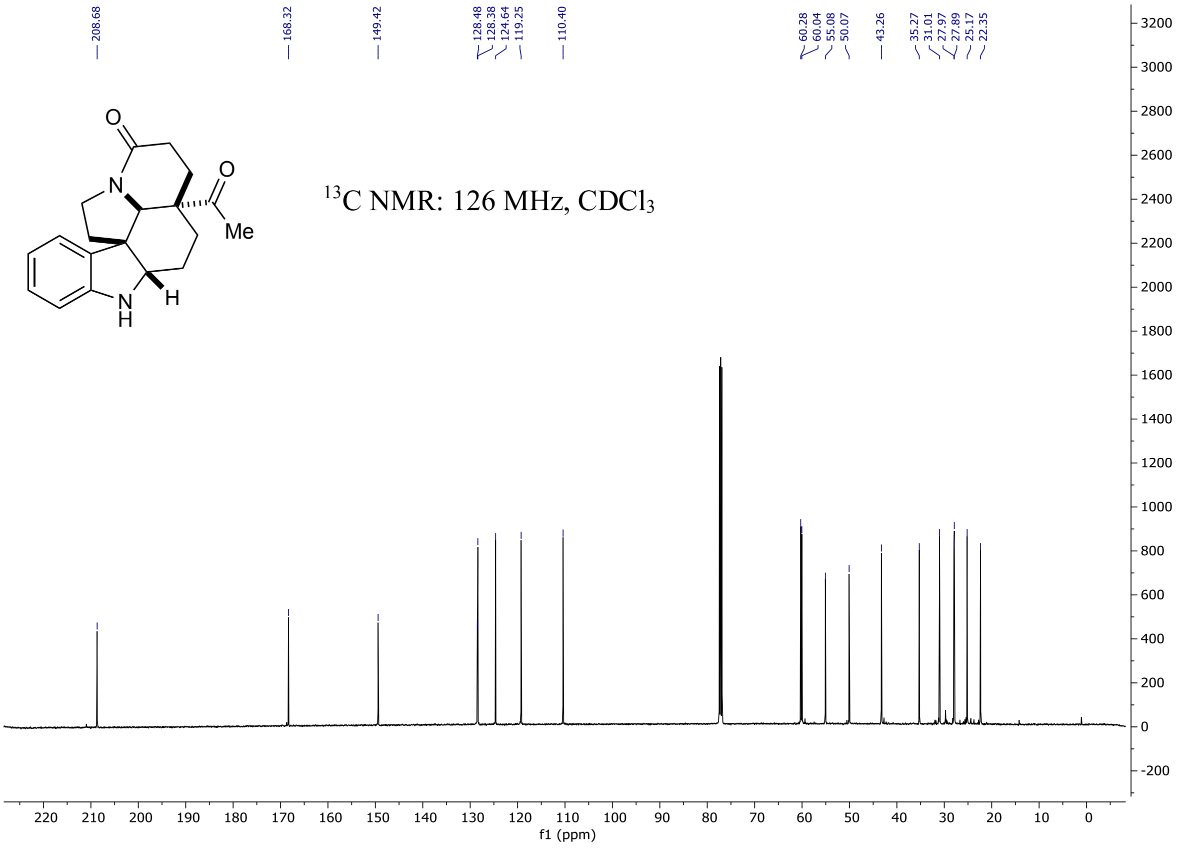

208.68
168.32
149.42
128.48
128.38
124.64
119.25
110.40
60.28
60.04
55.08
50.07
43.26
35.27
31.01
27.97
27.89
25.17
22.35
O
N
O
Me
N
H
H
^{13}C NMR: 126 MHz, CDCl3
f1 (ppm)

Compound 24

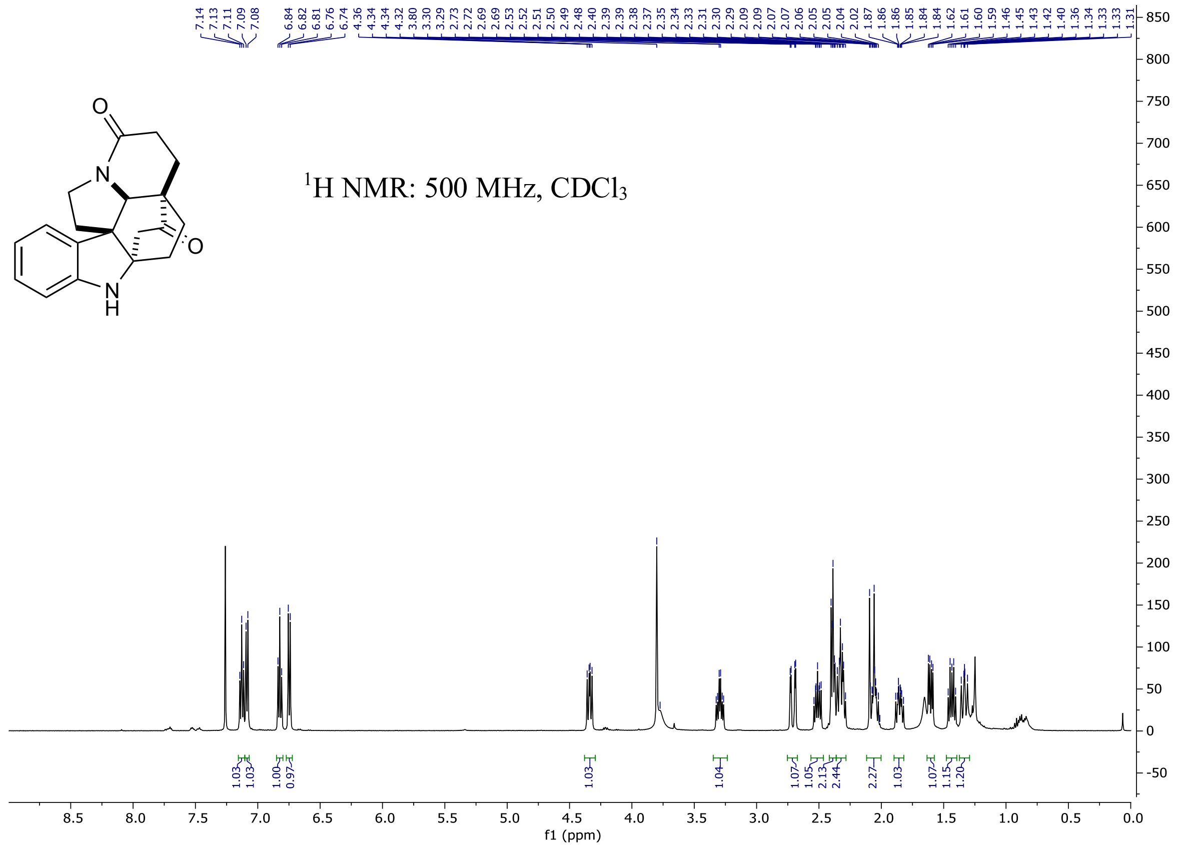

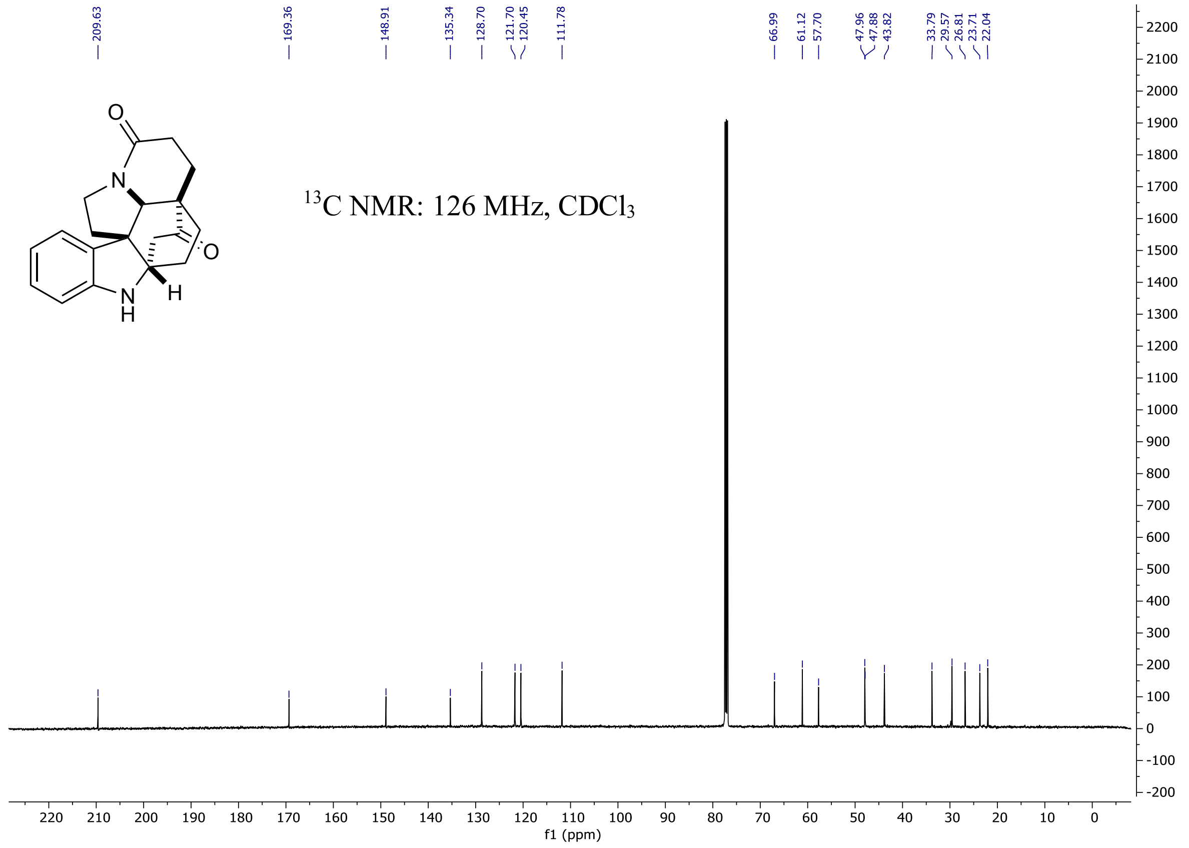

Compound 25

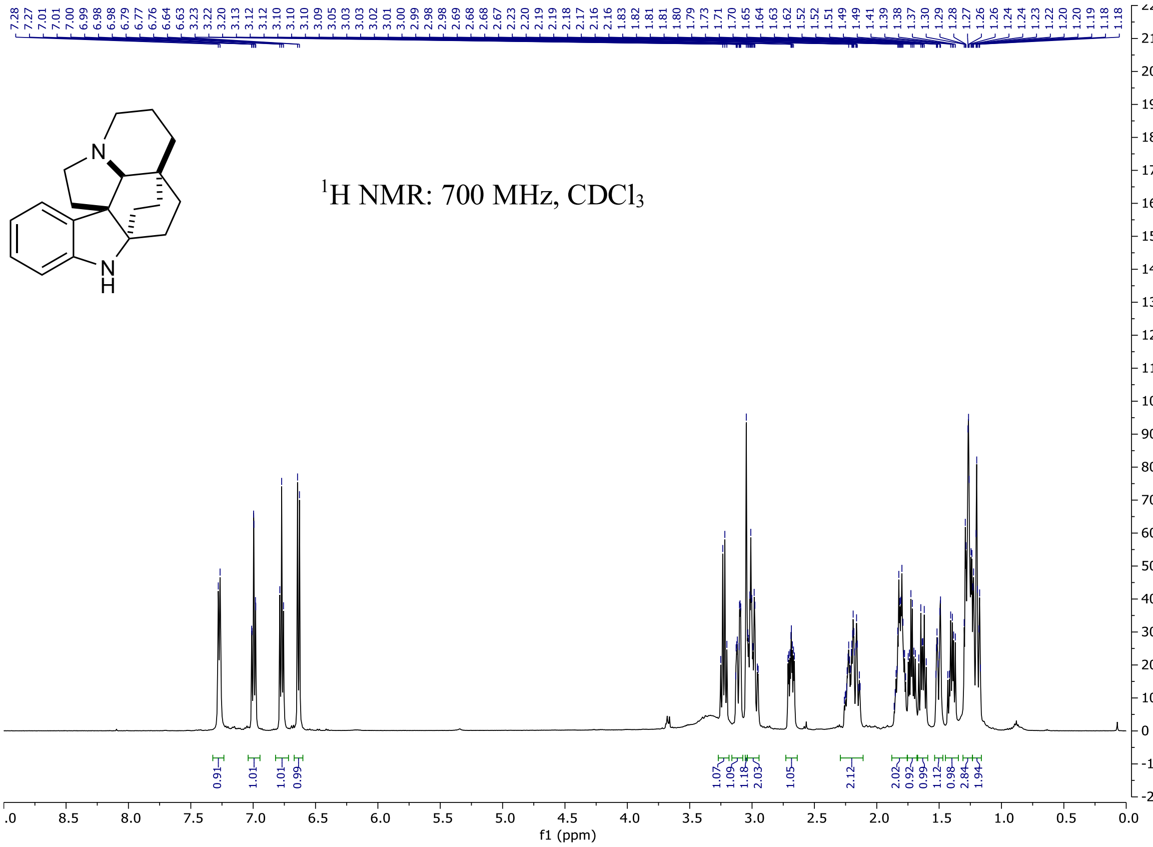

^{1}H NMR: 700 MHz, CDCl$_3$
7.28 7.27 7.01 7.01 7.00 6.99 6.98 6.98 6.79 6.77 6.76 6.64 6.63 3.23 3.22 3.20 3.13 3.12 3.12 3.10 3.10 3.10 3.09 3.05 3.03 3.03 3.02 3.01 3.00 2.99 2.98 2.98 2.69 2.68 2.68 2.67 2.23 2.20 2.19 2.19 2.18 2.17 2.16 2.16 1.83 1.82 1.81 1.81 1.80 1.79 1.73 1.71 1.70 1.65 1.64 1.63 1.62 1.52 1.52 1.51 1.49 1.49 1.41 1.39 1.38 1.37 1.30 1.29 1.28 1.27 1.26 1.24 1.24 1.23 1.22 1.20 1.19 1.18 1.18
0.91 1.01 1.01 0.99 1.07 1.09 1.18 2.03 1.05 2.12 2.02 0.92 0.99 1.12 0.98 2.84 1.94
f1 (ppm)

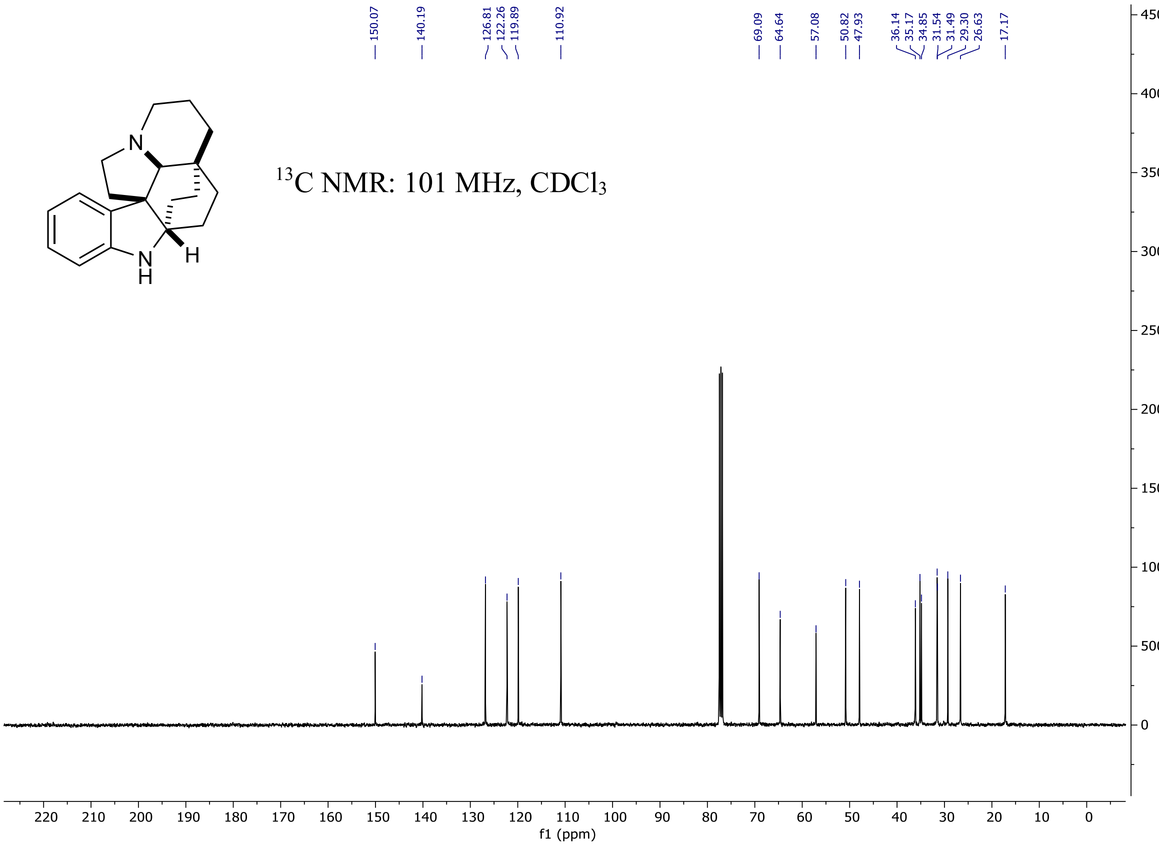

^{13}C NMR: 101 MHz, CDCl$_3$
150.07 140.19 126.81 122.26 119.89 110.92 69.09 64.64 57.08 50.82 47.93 36.14 35.17 34.85 31.54 31.49 29.30 26.63 17.17
f1 (ppm)
